# Flexible Regulation of Optical Properties Based on Structure Size‐Driven Intermolecular Interactions for Phototherapy

**DOI:** 10.1002/advs.202501468

**Published:** 2025-04-24

**Authors:** Zhichao Gong, Guangbo Kang, Yu Cao, Jiachen Pan, Xuejiao Rong, Xiaobing Du, Danping Zhang, He Huang, Shuxian Meng

**Affiliations:** ^1^ School of Chemical Engineering and Technology State Key Laboratory of Synthetic Biology, Tianjin Key Laboratory of Biological and Pharmaceutical Engineering Tianjin University Tianjin 300350 P. R. China; ^2^ College of Chemical Engineering Zhejiang Province Key Laboratory of Biofuel Zhejiang University of Technology Hangzhou 310014 P. R. China

**Keywords:** acq@aie bimolecular system, flexible regulation, optical properties, phototherapy, structure size‐driven intermolecular interactions

## Abstract

The precise control of optical properties in molecular systems remains a challenge for phototherapy. Herein, the strategic combination of aggregation‐caused quenching (ACQ) and aggregation‐induced emission (AIE) molecule creates ACQ@AIE bimolecular systems with tunable optical properties, which are almost unattainable by single‐component materials. Through systematic investigation of three ACQ@AIE bimolecular systems, it is established that molecule structure size differentials dictate their intermolecular interactions and consequent optical behaviors. Crucially, AIE molecule with a smaller structure size promotes ACQ molecule clustering to enhance the photothermal effect, while when the size becomes larger, particularly approaching that of ACQ molecule, facilitating π–π stacking and boosting the photodynamic effect. These distinct assembly modes revealed through combined experimental and theoretical analyses, enable precise regulation of photothermal versus photodynamic effects by simply regulating the structure size and ratio of ACQ and AIE molecules. Building on these mechanistic insights, the optimal molecule combination of ACQ@AIE bimolecular system is engineered into nanoparticles that exhibit mild photothermal effect, strong photodynamic effect, and excellent tumor accumulation and retention, achieving near‐complete tumor eradication with minimal treatment cycles while maintaining good biosafety. This work not only elucidates the fundamental structure size‐interaction‐property relationships in ACQ@AIE bimolecular systems but also provides generalizable strategies for developing intelligent photo theranostic materials through controlled intermolecular interaction.

## Graphical abstract

Herein, ACQ@AIE bimolecular systems are developed by noncovalently combining an aggregated‐caused quenching (ACQ) molecule with three aggregated‐induced emission (AIE) molecules. Differentials in structural size drive different patterns of intermolecular interactions, leading to distinct and tunable optical properties. Utilizing this property, nanoassemblies with balanced photothermal and photodynamic effects are developed to improve the efficacy of phototherapy while reducing side effects.

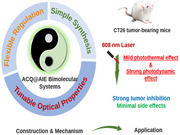

## 1 Introduction

Cancer remains a leading threat to global health, making urgent demand for advanced therapies.^[^ ^1^ ^]^ For tumor therapy, phototherapy has emerged as a promising approach due to its spatiotemporal precision and minimal invasiveness.^[^ ^2^ ^]^ Phototherapy mainly includes photothermal therapy (PTT) and photodynamic therapy (PDT), and the efficacy of these phototherapeutic strategies critically depends on the molecular design of organic photosensitizers,^[^ ^3^ ^]^ where optimized photothermal conversion efficiency (PCE) and reactive oxygen species (ROS) generation capacity are essential for effective tumor ablation.^[^ ^4^ ^]^ Current phototherapeutic development primarily focuses on two classes of chromophores: aggregate‐caused quenching (ACQ) and aggregate‐induced emission (AIE) molecules (ACQs and AIEs).^[^ ^5^ ^]^ Due to their respective properties, the combined use of ACQs and AIEs can endow molecules with a variety of optical properties.^[^ ^6^ ^]^ While their complementary properties offer opportunities for the combination, significant challenges persist:^[^ ^7^ ^]^ 1) unpredictable structure‐property relationships requiring extensive trial‐and‐error synthesis, 2) elevated toxicity risks from complex molecular architectures, and 3) substantial development costs for integrated multimodal agents. These limitations underscore the need for innovative design strategies that balance performance with synthetic accessibility.

An effective method for modifying optical properties is through the regulation of molecular structure interactions. Li et al.^[^ ^8^ ^]^ proposed an innovative strategy based on the concept of “noncovalent conformational lock” to effectively regulate the molecular conformation, providing a delicate balance between radiative and non‐radiative energy dissipation and constructing molecules with high molar extinction coefficients, excellent PCE and second near‐infrared (NIR‐II) fluorescence emission. Yu et al.^[^ ^9^ ^]^ developed temperature‐responsive systems where intramolecular rotation modulates ROS generation. The restricted motion enhanced ROS at lower temperatures while elevated temperatures promoted nonradiative decay.

However, the above methods still have difficulties and high costs for the design and synthesis. Notably, the utilization of intermolecular interactions can also impart materials with satisfactory optical properties,^[^ ^10^ ^]^ enabling flexible regulation of the phototherapy performance. In recent years, our team has reported some examples of intermolecular interactions in modulating optical properties for phototherapy.^[^ ^11^ ^]^ First, we stumbled upon the unique synergistic effect of PDT and PTT brought about by the combination of borondipyrromethene (BODIPY) and tetraphenylethylene (TPE).^[^ ^11a,b^ ^]^ Then, it was found that the intriguing optical properties due to intermolecular interactions are present not only in BODIPY and TPE but also between more ACQs and AIEs.^[^ ^11c^ ^]^ Furthermore, it was revealed that intermolecular interactions are the core mechanism determining the optical properties and photodynamic efficacy of metal–organic frameworks. In other teams, researchers have achieved good results.^[^ ^11d^ ^]^ Hu et al.^[^ ^12^ ^]^ combined two organic semiconducting molecules to promote the separation of electron‐hole pairs, which in turn facilitated the type I PDT pathway. However, the relationship between the mechanism of intermolecular interactions and molecular‐specific molecular structural features needs to be further explored.

In the field of phototherapy, the use of photothermal/photodynamic synergistic therapy has been demonstrated to yield favorable outcomes.^[^ ^13^ ^]^ However, in the case of PTT, high temperature will easily damage normal tissues and induce unnecessary side effects.^[^ ^14^ ^]^ Conventional mitigation strategies involving dose reduction or laser power attenuation often compromise therapeutic efficacy by simultaneously diminishing fluorescence intensity and photodynamic effects. Consequently, the development of photo theranostic materials with flexibly tunable properties based on intermolecular interactions for tumor therapeutic purposes remains a significant challenge.

In this study, a novel class of ACQ@AIE bimolecular systems with flexibly tunable optical properties were introduced for tumor phototherapy. By strategically pairing an ACQ core (named BBTD) with AIE molecules (named TPA3OMe, KZTPA, TKZTPA, AIEs) of different structure sizes, we established two key design principles. First, complementary electrostatic interactions separate the highest occupied molecular orbitals (HOMOs) and lowest unoccupied molecular orbitals (LUMOs), reducing Δ *E st* for enhanced energy utilization for heat or ROS generation. Second, AIEs structure size drives assembly morphology. AIEs with smaller structure sizes promote J‐aggregation of BBTD, amplifying NIR absorption/emission and nonradiative decay (photothermal‐dominant). Larger AIEs disrupt J‐aggregation via strong π‐stacking, suppressing heat release while activating ROS production (photodynamic‐dominant). Optimal regulation occurs at matched molecular sizes (BBTD@KZTPA), where optimal intermolecular interactions enable predictable property regulation. The optimized BBTD@KZTPA system, exhibiting balanced photothermal/photodynamic performance, was selected for the preparation of nanoparticles (NPs) for tumor phototherapy through the encapsulation of DSPE‐mPEG_2000_. It was found that the optical properties could be regulated not only by the molar ratio of molecules but also by the spatially confined domains. The NPs achieved complete ablation after two treatments during 12 days with no systemic toxicity, realizing the goal of achieving flexible regulation, lower cost, and high tumor therapeutic efficacy with intermolecular interactions, which is difficult to achieve by the single‐molecule systems reported in the literature above.^[^ ^3^, ^4^, ^5^, ^8^, ^13^, ^14^ ^]^

## 2 Result and Discussion

### 2.1 Construction and Properties of Bimolecular Systems

First, We synthesized the ACQ molecule BBTD based on a BODIPY core (Figure S1, Supporting Information) and three AIE molecules with progressively larger structures by incorporating rotatable groups following the restriction of intramolecular motions (RIM) theory^[^ ^15^ ^]^ (Figure S2–S4, Supporting Information), with their ACQ/AIE properties confirmed through fluorescence studies in mixed solvents (Figure S20, Supporting Information). Subsequently, as shown in **Scheme** **1**a, ACQ@AIE bimolecular systems, i.e., BBTD@AIEs, were constructed via D‐A interactions under the environment of DMF/water, and the changes in optical properties of BBTD@AIEs compared to BBTD were investigated.

Scheme 1Schematic illustration for this study: a) construction, b) mechanism, and c) application.

**Figure:**
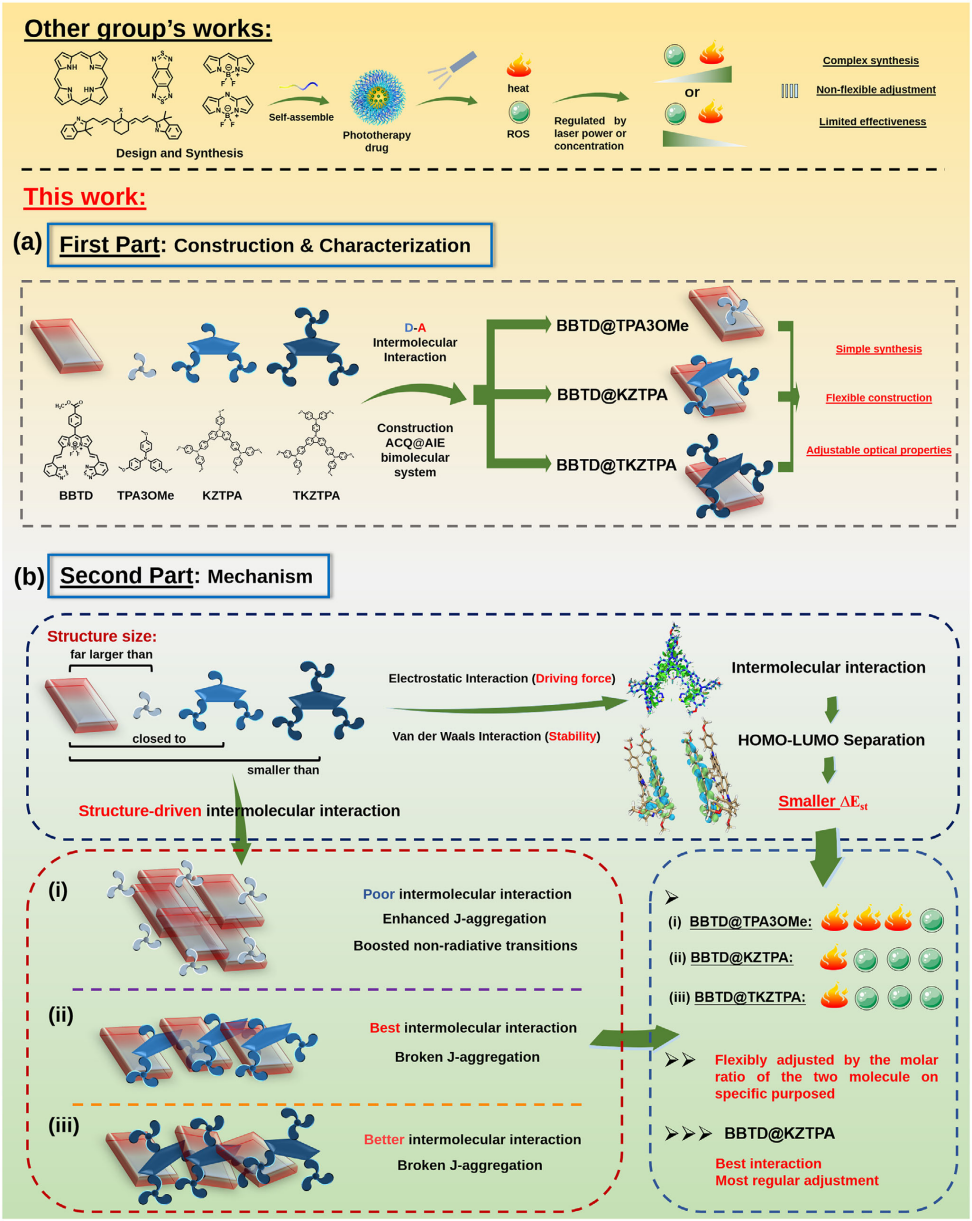

**Figure:**
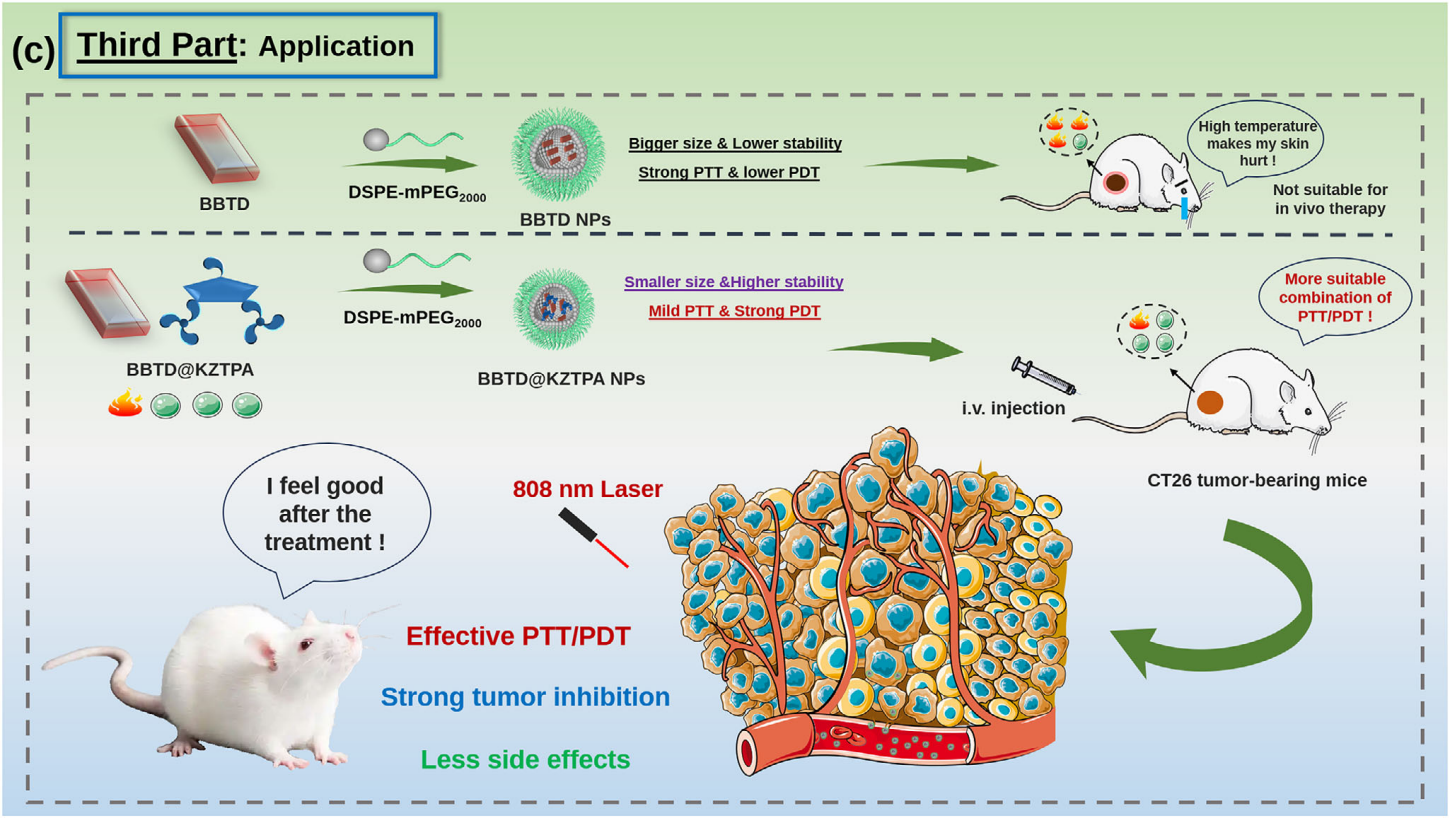

**Figure 1:**
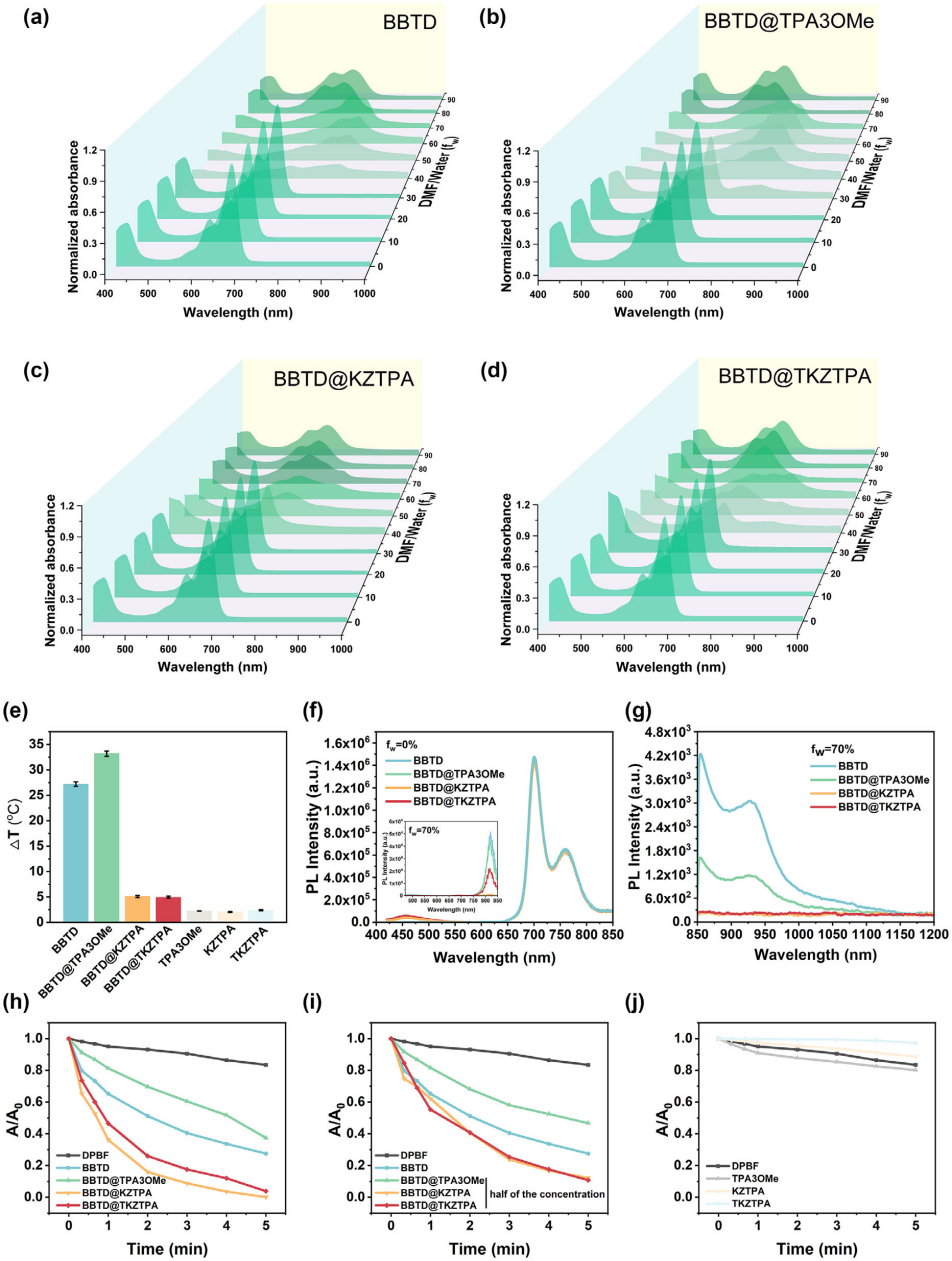
Normalized UV–vis absorbance of a) BBTD, b) BBTD@TPA3OMe, c) BBTD@KZTPA, d) BBTD@TKZTPA under DMF/water with different fractions of water. e) Photothermal effect of BBTD, BBTD@TPA3OMe, BBTD@KZTPA, BBTD@TKZTPA, TPA3OMe, KZTPA and TKZTPA. f) Fluorescence emission of BBTD, BBTD@TPA3OMe, BBTD@KZTPA and BBTD@TKZTPA under DMF/water (f_w_ = 0% and 70%). g) NIR fluorescence emission of BBTD, BBTD@TPA3OMe, BBTD@KZTPA and BBTD@TKZTPA under DMF/water (f_w_ = 70%). h–j) Photodynamic effect of BBTD, BBTD@TPA3OMe, BBTD@KZTPA, BBTD@TKZTPA, TPA3OMe, KZTPA and TKZTPA. (For Figure 1i, the concentration of BBTD@AIEs is 12.5@12.5 µm.).

The results of UV–vis absorbance variation with water fraction (f_w_) revealed the BBTD alone exhibited J‐aggregation at f_w_ = 40% and transitioned to H‐aggregation at f_w_ = 80% (**Figure** **1**a; Figure S19, Supporting Information), while BBTD@TPA3OMe showed enhanced J‐aggregation (f_w_ = 30–60%) with stronger absorption at 808 nm (A_808_) and NIR fluorescence (Figure 1b; Figure S22a,b, Supporting Information), which was disrupted at f_w_ = 70% due to spatial constraints. In contrast, BBTD@KZTPA and BBTD@TKZTPA displayed significantly weaker absorbance redshifts (Figure 1c,d), indicating their disruptive effect on J‐aggregation, particularly for the more structurally close KZTPA system. Fluorescence emission studies demonstrated quenching through intermolecular π–π stacking, with the extent of quenching correlating with AIE molecule structure size (Figure 1f,g), suggesting that smaller TPA3OMe weakly interacts with BBTD but larger KZTPA/TKZTPA engage in stronger interactions that inversely correlate with J‐aggregation promotion.

Further, photothermal and photodynamic effects characterization revealed distinct structure size‐property relationships (Figure 1e,h–j). While BBTD@TPA3OMe exhibited photothermal synergy coupled with photodynamic antagonism, both BBTD@KZTPA and BBTD@TKZTPA demonstrated the inverse behavior of photothermal antagonism with photodynamic synergy. Electron paramagnetic resonance (EPR) spectroscopy and singlet oxygen  (^1^O_2_) probe experiments confirmed the generation of ^1^O_2_ as the primary reactive oxygen species (Figure S23 and S24, Supporting Information), and the results were fully consistent with the observed photodynamic effect trends.

### 2.2 Flexible Regulation of Optical Properties in Bimolecular Systems

**Figure 2:**
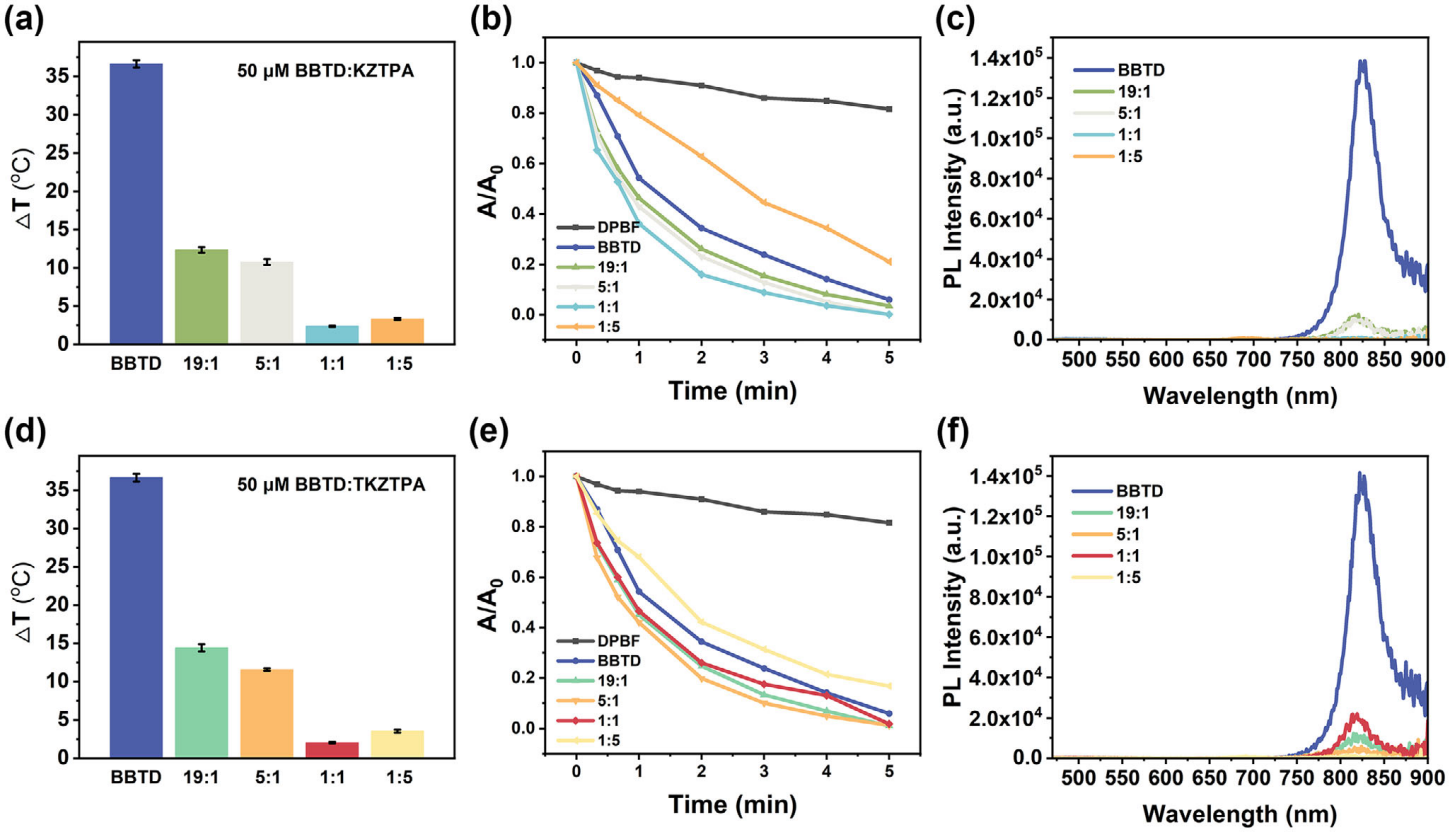
a–c). Photothermal effect, photodynamic effect, and fluorescence emission of BBTD@KZTPA with different molar ratios. d–f) Photothermal effect, photodynamic effect, and fluorescence emission of BBTD@TKZTPA with different molar ratios.

Conventional PTT often risks damaging healthy tissues due to excessive local heating. To address this, an effort was made to leverage the flexible and tunable optical properties of ACQ@AIE bimolecular systems to achieve an optimal balance between photothermal and photodynamic effects, specifically, by reducing the required temperature while enhancing ^1^O_2_ generation. Because BBTD@TPA3OMe exhibited photothermal synergy but photodynamic antagonism (contrary to the goal), we successfully achieved precise regulation of these properties by modulating the molar ratios of BBTD to AIEs (KZTPA and TKZTPA) in BBTD@KZTPA and BBTD@TKZTPA. As shown in **Figure** **2**, increasing the AIEs ratio progressively decreased fluorescence intensity and temperature (Figure 2a,c,e,f) while enhancing ^1^O_2_ production (Figure 2b,d). Notably, BBTD@KZTPA demonstrated a more consistent and predictable modulation trend than BBTD@TKZTPA, likely due to the closer structural match between KZTPA and BBTD, which facilitated stronger and more uniform intermolecular interactions. However, at a 1:5 molar ratio, this trend deviated, possibly due to the reduced A_808_ (Figure S21, Supporting Information).

### 2.3 Exploration of Mechanism

**Figure 3:**
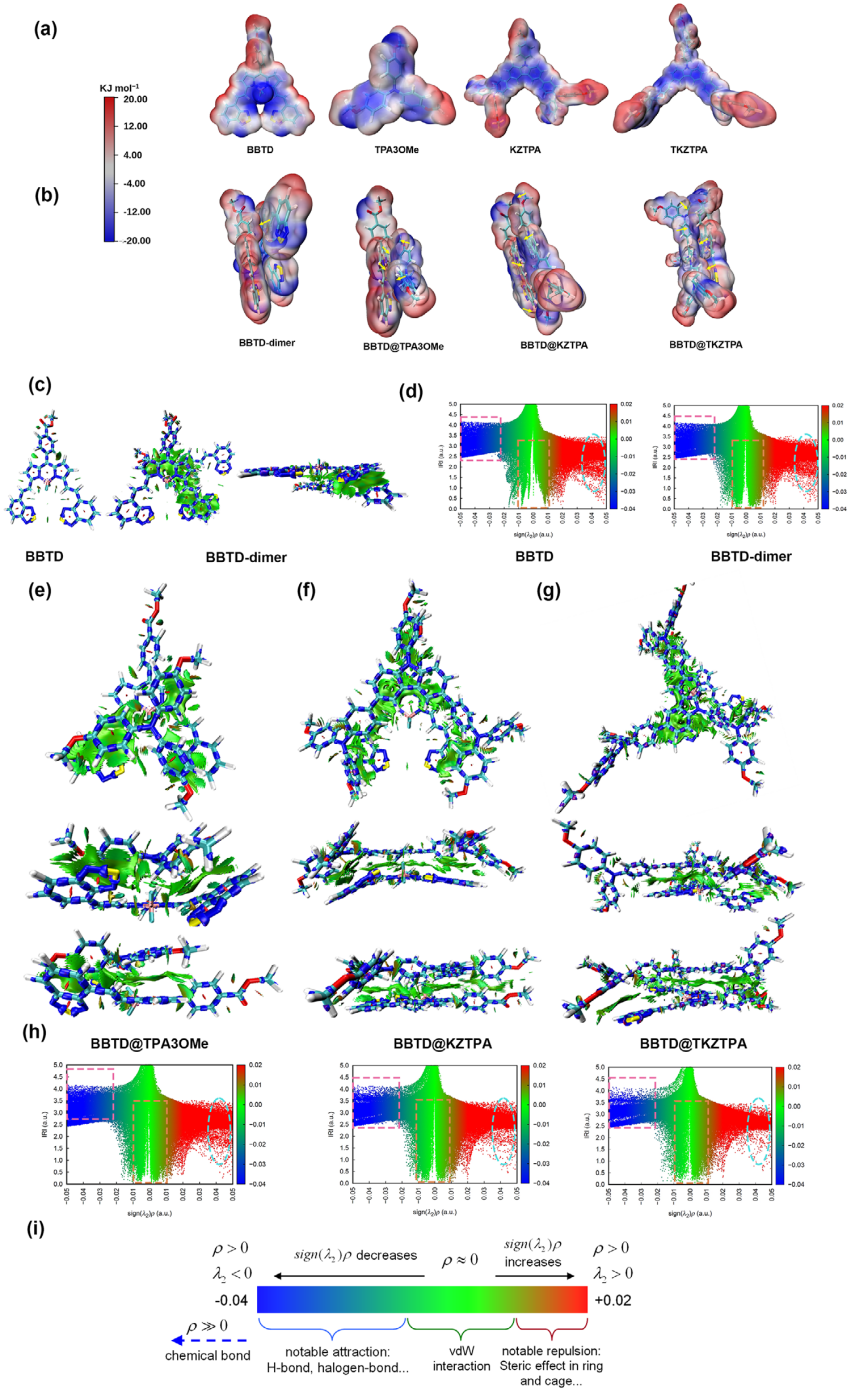
a) ESP distribution map of BBTD, TPA3OMe, KZTPA, and TKZPTA. b) ESP penetration map of BBTD‐dimer, BBTD@TPA3OMe, BBTD@KZTPA and BBTD@TKZTPA. c) Isosurface map of BBTD and BBTD‐dimer (IRI = 1.1). d) Scatter map between IRI and *sign*(λ_2_)*ρ* of BBTD and BBTD‐dimer. e–g) Isosurface map of BBTD@TPA3OMe, BBTD@KZTPA and BBTD@TKZTPA (IRI = 1.1). h) Scatter map between IRI and *sign*(λ_2_)*ρ* of BBTD@TPA3OMe, BBTD@KZTPA and BBTD@TKZTPA. i) Standard coloring method and chemical explanation of *sign*(λ_2_)*ρ* on IRI isosurfaces.

To elucidate the underlying mechanisms, we performed systematic theoretical calculations. Molecular conformations were initially screened using xtb,^[^ ^16^ ^]^ followed by optimization with Molclus^[^ ^17^ ^]^ and Gaussian 09.^[^ ^18^ ^]^ Through Multiwfn^[^ ^19^ ^]^ and VMD,^[^ ^20^ ^]^ Electrostatic potential (ESP)^[^ ^21^ ^]^ surface analysis revealed distinct charge distributions: BBTD exhibited an electron‐deficient character, while TPA3OMe, KZTPA and TKZTPA showed electron‐rich surfaces, with KZTPA and TKZTPA displaying higher charge density (**Figure** **3**a). This complementary charge distribution facilitates the formation of BBTD@AIEs through electrostatic interactions. Comparative analysis of the BBTD‐dimer extracted from crystal structure and BBTD@AIE systems through ESP penetration mapping (Figure 3b) indicated significantly weaker charge overlap (red‐blue regions) in the BBTD‐dimer, consistent with its predominant reliance on van der Waals forces. In contrast, BBTD@AIEs showed markedly enhanced electrostatic adsorption that correlated directly with the increasing surface charge density of the AIEs, providing quantitative evidence for the stronger intermolecular interactions in these systems.

To further elucidate the intermolecular interactions, we employed the Interaction Region Indicator (IRI) method.^[^ ^22^ ^]^ The analysis revealed that both BBTD‐dimer and BBTD@AIEs are primarily governed by weak van der Waals interactions (Figure 3c,f,g). More quantitatively, the scatter plots of IRI versus *sign*(λ_2_)*ρ* revealed a striking size‐dependent trend: increasing the AIEs molecular size led to attenuated steric repulsion but simultaneously enhanced noncovalent attractive interactions (Figure 3d,h). Particularly noteworthy was the BBTD@KZTPA system, where the spatial distribution of green IRI regions exhibited remarkable consistency with the molecular structure contours (Figure 3f), reflecting exceptional intermolecular complementarity arising from their closely matched structural dimensions. This precise geometric alignment, combined with optimal charge distribution, accounts for the superior binding characteristics of BBTD@KZTPA compared to other bimolecular systems, as evidenced by both experimental measurements and theoretical calculations.

To clearly present the intermolecular interaction, the energy decomposition analysis based on the sobEDAw method was conducted (**Table** **1**).^[^ ^23^ ^]^ The energy decomposition analysis quantitatively revealed that electrostatic interactions dominated the BBTD@AIEs binding (−20.42 to −34.54 kJ mol^−1^), contributing 82.13 –85.57% of the total interaction energy–a stark contrast to the BBTD‐dimer's weaker van der Waals‐driven cohesion. It was noteworthy that that the dispersion and exchange‐repulsion interaction were also significant contributors to intermolecular interactions, and these interactions grew progressively stronger with AIEs structure size, which was consistent with the trend presented in the scatter map (Figure 3d,h). The pinnacle of stabilization (−40.63 kJ mol^−1^) occurred in size‐close BBTD@KZTPA, where the rotatable moieties of KZTPA enabled tighter orbital overlap than the rigid and planar BBTD‐dimer. This energy gap underscores how the structural flexibility of KZTPA outperforms static packing of BBTD‐dimer in optimizing intermolecular compatibility. Crucially, the inverse relationship between steric bulk and interaction strength confirms that balanced molecular dimensions are essential for maximizing intermolecular interaction synergies.

**Table 1:** Energy decomposition of intermolecular interactions (kJ mol^−1^) of BBTD, BBTD@TPA3OMe, BBTD@KZTPA, and BBTD@TKZTPA based on the sobEDAw method.

	E_ele_ ^a)^	E_x‐rep_ ^b)^	E_orb_ ^c)^	E_disp_ ^d)^	E_int_ ^e)^	E_ele_/E_int_
BBTD‐dimer	−23.70	59.03	−8.86	−57.06	−30.59	77.48%
BBTD@TPA3OMe	−20.42	56.43	−8.07	−52.81	−24.87	82.13%
BBTD@KZTPA	−34.10	88.31	−12.01	−82.83	−40.63	83.92%
BBTD@TKZTPA	−34.54	89.76	−12.32	−83.27	−40.37	85.57%

^a)^ Average electrostatic interaction energy;

^b)^ Average exchange‐repulsion interaction energy;

^c)^ Average orbital interaction energy;

^d)^ Average dispersion interaction energy;

^e)^ Average total interaction energy.

**Figure 4:**
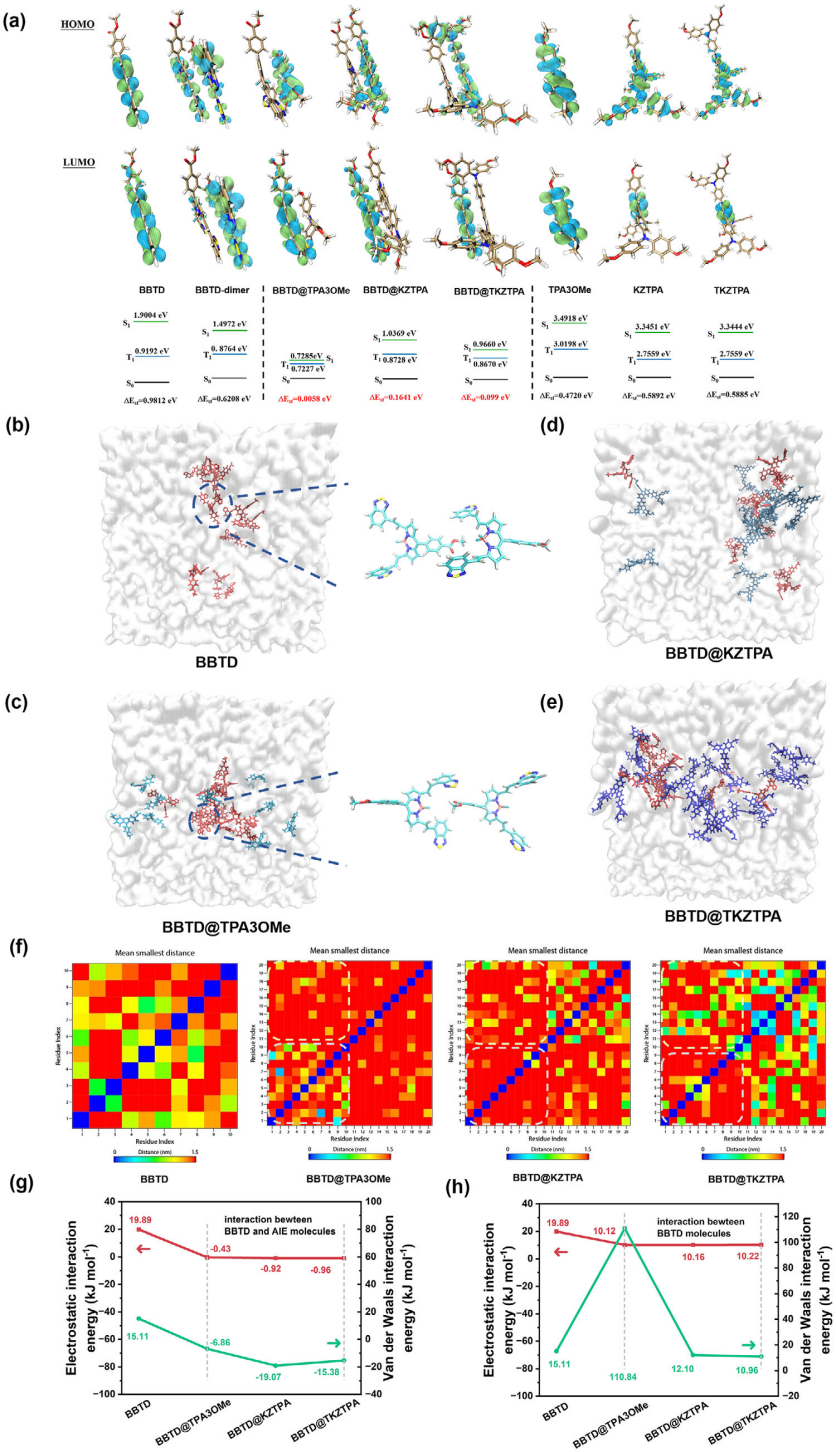
a) Distribution of HOMOs and LUMOs and the calculation results of excited state energy for BBTD, BBTD‐dimer, BBTD@TPA3Me, BBTD@KZTPA, BBTD@TKZTPA, TPA3OMe, KZTPA and TKZTPA. Results of MD simulation of b) BBTD, c) BBTD@TPA3Me, d) BBTD@KZTPA, and e) BBTD@TKZTPA under DMF/water (f_w_ = 70%) (BBTD molecules: red color; TPA3OMe molecules: cyan color; KZTPA molecules: gray‐blue color; TKZTPA molecules: blue color). f) Color‐filled map of the mean smallest intermolecular distances in BBTD, BBTD@TPA3Me, BBTD@KZTPA, and BBTD@TKZTPA (Residue index 1–10 for BBTD and 11–20 for AIEs). Comparison of *E_ele_* and *E_vdW_* for g) two kinds of molecules and h) BBTD molecules in bimolecular systems.

The redistribution of HOMOs and LUMOs in BBTD@AIE systems was investigated to elucidate their optical property changes. Compared to single components (BBTD, BBTD‐dimer, TPA3OMe, KZTPA, TKZTPA), the HOMOs became predominantly localized on the AIEs units (TPA3OMe/KZTPA/TKZTPA), while the LUMOs remained centered on BBTD (**Figure** **4**a), inducing significant excited‐state energy changes. The reduced Δ*E*_st_ enabled more concentrated energy utilization: 1) For BBTD@TPA3OMe (Δ*E*_st_ = 0.0058 eV), the reduced gap facilitated reverse intersystem crossing (RISC), promoting nonradiative decay and heat release, and the S_1_‐S_0_ energy reduction further enhanced non‐radiative transition (photothermal synergy); 2) In contrast, BBTD@KZTPA and BBTD@TKZTPA exhibited larger Δ*E*_st_ values (0.098 and 0.1641 eV, respectively), favoring intersystem crossing (ISC) to triplets and subsequent ^1^O_2_ generation (photodynamic synergy). These distinct energy differences rationalize the opposing photothermal/photodynamic behaviors observed experimentally.

In order to explore the differences in optical properties between BBTD and BBTD@AIEs more deeply, molecular dynamics (MD) simulations under DMF/water (f_w_ = 70%) environment were carried out by Gromacs software.^[^ ^24^ ^]^ The results revealed distinct assembly behaviors between the systems. For BBTD alone and BBTD@TPA3OMe, J‐aggregation dominated (Figure 4b,c), consistent with UV–vis absorbance trends (Figure 1a–d). In contrast, BBTD@KZTPA and BBTD@TKZTPA showed entirely different configurations (Figure 4d,e). Specifically, KZTPA and TKZTPA molecules engaged in “face‐to‐face” π–π stacking with BBTD molecules, while TPA3OMe molecules preferentially crowded BBTD molecules together without direct stacking. Quantitative analysis of intermolecular distances (Figure 4f) further demonstrated that increasing AIEs structure size progressively enhanced the binding of BBTD and AIEs and increased BBTD molecule distances, revealing a size‐dependent binding preference. Notably, the larger KZTPA and TKZTPA molecules (with higher charge density) formed stable electrostatic complexes with BBTD molecules at closer distances, whereas the smaller TPA3OMe molecules (with lower charge density) predominantly induced steric crowding of BBTD molecules in the confined environment. These findings establish that subtle variations in AIEs structure features, particularly size and charge distribution, govern the fundamental intermolecular interaction modes and ultimately determine the properties of these bimolecular systems.

MD with force field calculations quantitatively elucidated the structure‐energy‐property relationships in these systems. The electrostatic (*E*_ele_) and van der Waals (*E*_vdW_) interaction energy analysis revealed three distinct regimes: 1) For BBTD system, strong positive repulsion dominated due to its electron‐deficient nature (*E*_ele_ to 19.89 kJ mol^−1^; *E*_vdW_ to 15.11 kJ mol^−1^, Figure 4g); 2) In BBTD@AIEs, the interactions transitioned to attractive (*E*_ele_ down to −0.96 kJ mol^−1^; *E*_vdW_ down to −19.07 kJ mol^−1^, Figure 4g), and BBTD@KZTPA showed optimal intermolecular interactions; 3) Notably, BBTD@TPA3OMe exhibited unique behavior, and its small size and low charge density caused elevated BBTD‐BBTD repulsion (*E*_vdW_ = 110.84 kJ mol^−1^, Figure 4h), forcing molecular crowding that enhanced nonradiative decay. The quantitative energy differences between these systems indicate how subtle structural variations in AIEs (size and charge distribution) dramatically alter both molecular packing and optical properties outcomes.

To further investigate the role of TPA3OMe in facilitating J‐aggregation, we conducted comparative MD simulations in DMF/water (f_w_ = 60%). The analysis revealed that BBTD@TPA3OMe systems exhibited significantly more J‐aggregation conformations than BBTD alone (Figure S29a,c, Supporting Information). This enhancement could be attributed to the expected increase in intermolecular distances at reduced water content and the specific ability of TPA3OMe molecules to promote BBTD molecules cluster through intermolecular interactions. It was noteworthy that the distance‐dependent analysis (Figure S29b,d, Supporting Information) demonstrated that TPA3OMe molecules actively mediate BBTD's self‐assembly into J‐aggregation rather than simply following concentration‐driven aggregation behavior. These simulations provide direct evidence for the unique capacity of TPA3OMe molecules to template J‐aggregation formation under conditions.

### 2.4 Construction and Properties of Bimolecular Nanoparticles

Based on the evaluation of intermolecular interactions, BBTD and BBTD@KZTPA were self‐assembled with DSPE‐mPEG_2000_ to form nanoparticles (BBTD NPs and BBTD@KZTPA NPs). By varying the amount of DSPE‐mPEG_2000_, we prepared two formulations: BBTD@KZTPA NPs A (higher DSPE‐mPEG_2000_ content) and BBTD@KZTPA NPs B (lower DSPE‐mPEG2000 content). The encapsulation efficiencies, determined via absorption standard curves (Figure S25, Supporting Information), were ≈48.10% for BBTD NPs, 47.89% for BBTD@KZTPA NPs A, and 46.08% for BBTD@KZTPA NPs B

**Figure 5:**
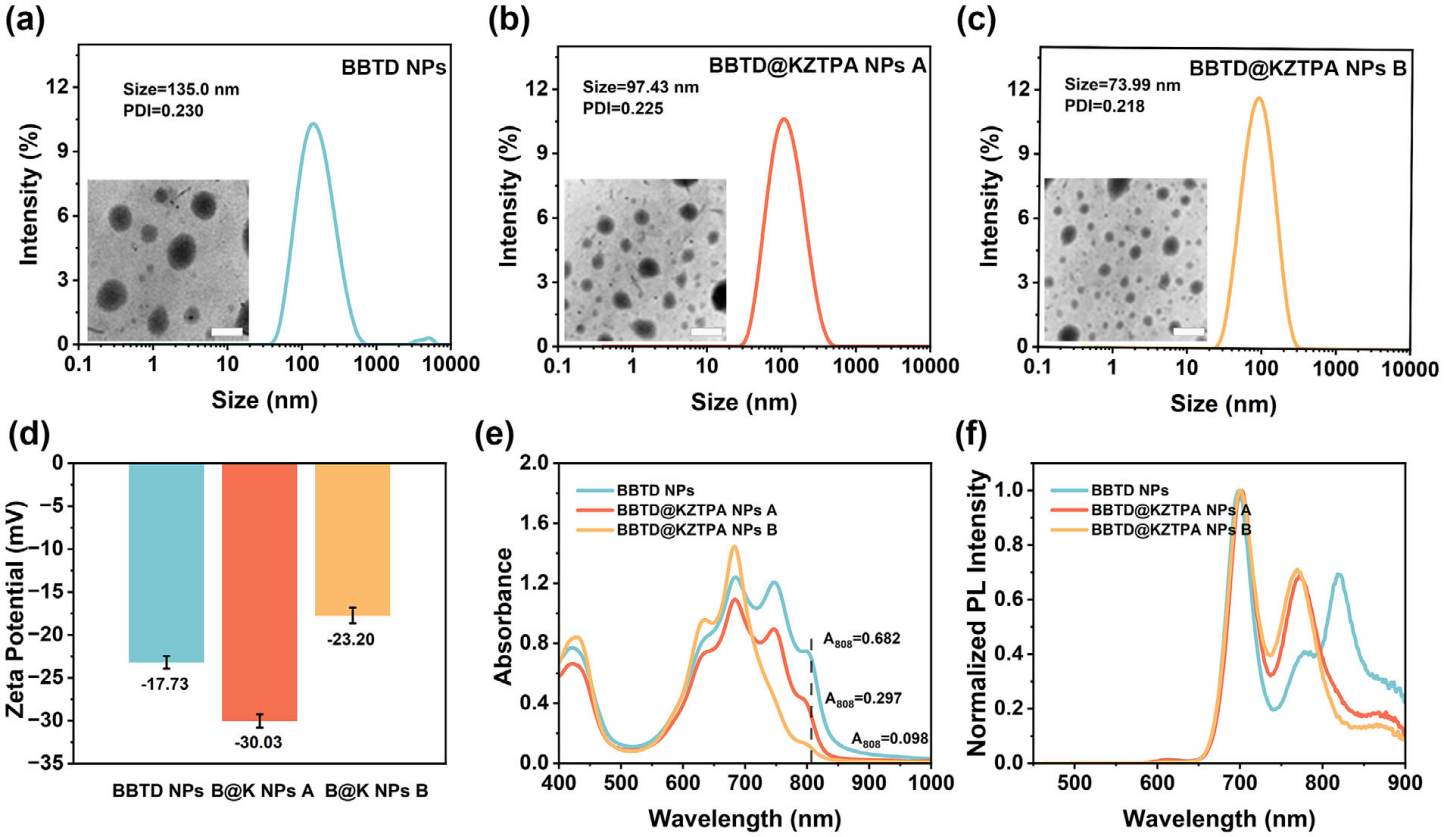
Size distribution and TEM graph of a) BBTD NPs, b) BBTD@KZTPA NPs A, and c) BBTD@KZTPA NPs B (Scale bar: 200 µm). Comparison of d) zeta potentials, e) UV–vis absorbance, and f) normalized fluorescence emission of BBTD NPs, BBTD@KZTPA NPs A and BBTD@KZTPA NPs B.

DLS analysis showed distinct hydrodynamic diameters for the three NPs: BBTD NPs (135.0 nm), BBTD@KZTPA NPs A (97.43 nm), and BBTD@KZTPA NPs B (73.99 nm) (**Figure** **5**a–c). This size progression revealed two key trends: 1) the intermolecular interaction of BBTD@KZTPA promoted more compact molecular packing, and 2) reducing the DSPE‐mPEG2000 content further decreased nanoparticle dimensions. Transmission electron microscopy (TEM) analysis confirmed these findings. It was noteworthy that BBTD@KZTPA NPs A exhibited distinctive shuttle‐shaped morphologies, suggesting that enhanced intermolecular interactions facilitate the formation of ordered nanoassemblies rather than amorphous structures.^[^ ^25^ ^]^ Zeta potential measurements revealed that BBTD@KZTPA NPs A possessed greater negative surface charge than BBTD NPs (Figure 5d), indicating better stability. Conversely, the reduced DSPE‐mPEG_2000_ content in formulation B led to molecular crowding in the hydrophobic core, reducing stability. These results collectively demonstrate that well‐ordered nanostructures achieved through optimized intermolecular interactions exhibit enhanced thermodynamic stability compared to their disordered counterparts.

The optical properties of BBTD, BBTD@KZTPA NPs A, and BBTD@KZTPA NPs B were systematically investigated through UV–vis absorption, fluorescence emission, and lifetime measurements (Figure 5e,f; Figure S26, Supporting Information). BBTD aggregation induced characteristic redshifts in both absorption and emission spectra; however, these shifts were less pronounced than those observed in DMF/water mixtures (f_w_ = 70%, Figure 1a). This result suggests that the confined hydrophobic environment within the nanoparticles partially restricts the formation of J‐aggregation. The incorporation of KZTPA further diminished these spectral shifts, indicating the disruption of J‐aggregation, which was consistent with the solution‐phase behavior shown in Figure 1c. Notably, reducing the hydrophobic core volume (from BBTD@KZTPA NPs A to B) enhanced this disruptive effect, as evidenced by the progressive decrease in A_808_ from 0.297 to 0.098 (Figure 5e). This trend indicates a strong correlation between nanoparticle architecture and molecular packing behavior.

**Figure 6:**
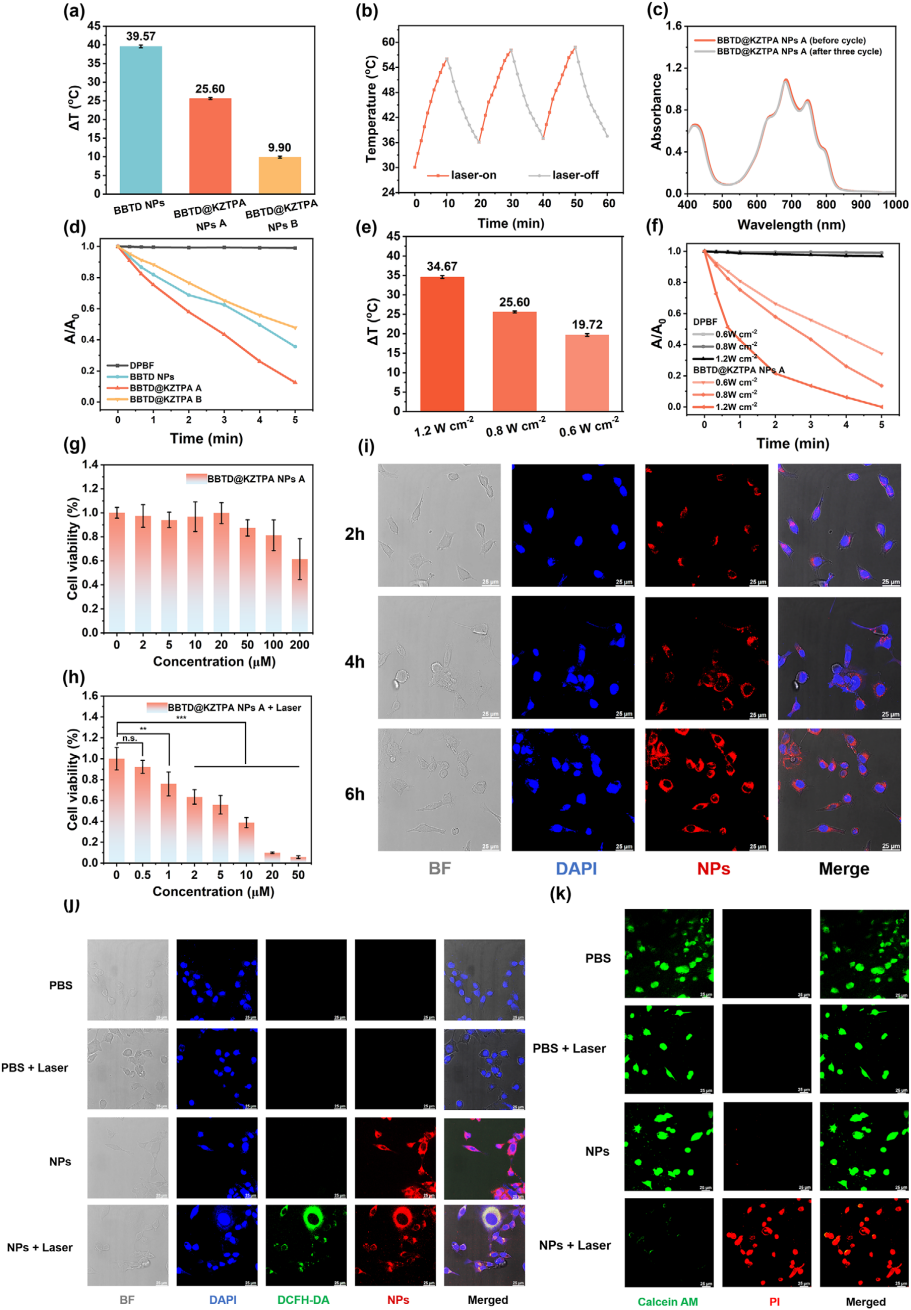
a) Photothermal performance comparison of BBTD NPs, BBTD@KZTPA NPs A, and BBTD@KZTPA NPs B under laser irradiation. b) Photothermal stability assessment of BBTD@KZTPA NPs A over three lasers on/off cycles. c) UV–vis absorption spectra of BBTD@KZTPA NPs A before and after photothermal cycling. d) Relative ROS generation efficiency of different nanoparticle formulations e) Laser power‐dependent photothermal and f) photodynamic effects of BBTD@KZTPA NPs A. Concentration‐dependent viability of CT26 cells treated with BBTD@KZTPA NPs A g) without and h) with laser irradiation (The statistical significance was assessed by Student's *t*‐test; *n* = 6; Data were presented as means ± SD; n.s.: not significant, ^**^ *p* < 0.01, ^***^ *p* < 0.001). i) Time‐dependent cellular uptake profiles. j) Intracellular ROS generation. k) Live/dead staining of CT26 cells after various treatments (Scale bar: 25 µm).

Comparative evaluation of photothermal and photodynamic performance revealed obvious differences between these NPs (**Figure** **6**a,d). Although BBTD@KZTPA NPs A showed reduced photothermal conversion relative to BBTD NPs, it exhibited significantly enhanced photodynamic effect, which were analogous to the behavior observed for the 5:1 molar ratio BBTD@KZTPA complex in solution (Figure 2a,b). The diminished A_808_ absorption of BBTD@KZTPA NPs B (Figure 5e) correlated with weaker photothermal and photodynamic effects. Crucially, despite these differences, BBTD NPs and BBTD@KZTPA NPs A maintained comparable photothermal conversion efficiencies (PCE) of 62.82% and 60.12%, respectively (Figure S27, Supporting Information). This similarity suggests that the BBTD@KZTPA system could effectively harness residual energy through optimized intermolecular interactions for ROS generation. These results demonstrate two complementary strategies for optical property modulation in the bimolecular system: molar ratio regulation and spatial confinement control. The consistent trends observed across both solution‐phase and nanoparticle systems underscore the remarkable flexibility of this platform for tailored phototherapeutic applications.

### 2.5 In Vitro Experiment

Given the superior performance profile, BBTD@KZTPA NPs A emerged as the most promising candidate for therapeutic applications. Therefore, more measurements were conducted to comprehensively evaluate its properties. The photothermal cycling curves and changes in absorbance revealed that BBTD@KZTPA NPs A and the intermolecular interaction characteristics exhibited excellent stability. (Figure 6b,c). As shown in Figure 6e,f, the photothermal and photodynamic effects exhibited a clear power‐dependent relationship, confirming the precise controllability of therapeutic outcomes.

Furthermore, BBTD@KZTPA NPs A was stored at room temperature for 7 days, and no significant changes were observed in the size, zeta potential, particle distribution index (PDI), and A_808_ (Figure S28, Supporting Information), which further confirmed the excellent therapeutic potentials of BBTD@KZTPA NPs A.

Subsequently, the phototherapeutic efficacy of BBTD@KZTPA NPs A was systematically evaluated in CT26 cells. Cytotoxicity assessment revealed excellent biocompatibility, with cell viability remaining >90% across concentrations up to 100 µm without laser irradiation (Figure 6g). However, upon 808 nm laser exposure (0.8 W cm^−2^, 5 min), the concentration‐dependent phototoxicity with 60% and 90% growth inhibition at 10 and 25 µm, respectively were observed (The half maximal inhibitory concentration was ≈7.5 µm). Furthermore, confocal laser scanning microscopy (CLSM) and quantitative intensity revealed time‐dependent cellular uptake (Figure 6i; Figure S30, Supporting Information). Additionally, it was noteworthy that under the 4',6‐diamidino‐2‐phenylindole (DAPI) channel, blue fluorescence was observed around the nucleus of the cells, which corresponded well with the red color. This phenomenon could be attributed to the fact that the test condition of the DAPI channel excited BBTD@KZPTA NPs A, resulting in blue fluorescence. Meanwhile, laser activation triggered significant ROS production and apoptosis induction (Figure 6j,k). These results, combining high biocompatibility, efficient cellular internalization, and potent photoactivatable cytotoxicity, confirm BBTD@KZTPA NPs A as a promising candidate for in vivo applications.

### 2.6 In Vivo Experiment

**Figure 7:**
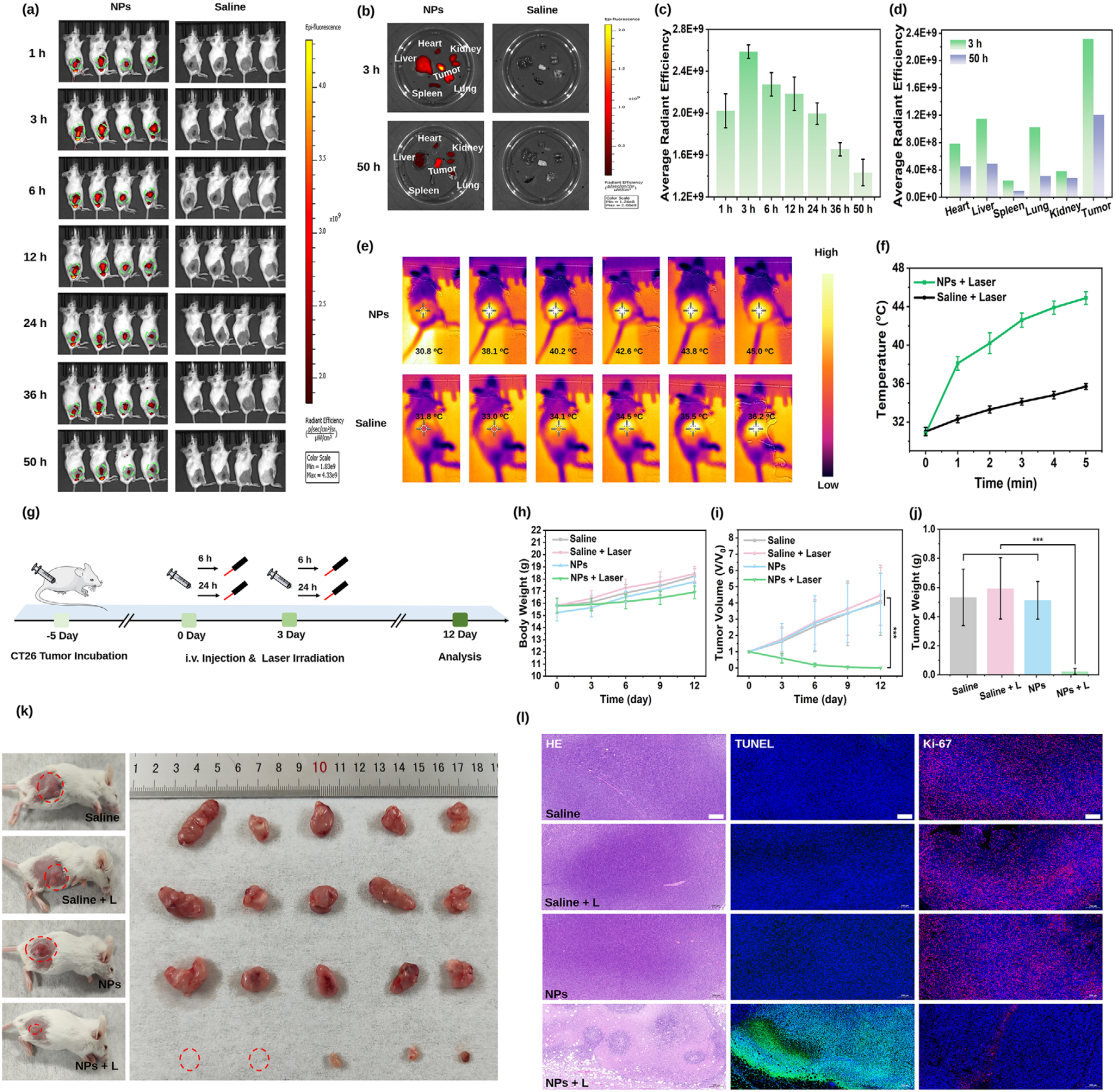
a) In vivo fluorescence images at various time points post‐injection of BBTD@KZTPA NPs‐A and saline. b) Ex vivo fluorescence images of major organs and tumors at 3 and 50 h post‐injection c) Average fluorescence intensity of tumor sites at various time periods (*n *= 4; Data were presented as means ± SD). d) Average fluorescence intensity of main organ and tumor at 3 and 50 h postinjection. e) Infrared thermal images of tumors at 6 h postinjection with BBTD@KZTPA NPs A and saline during 808 nm laser irradiation (0.8 W cm^−2^, 5 min). f) I Real‐time tumor temperature profiles during irradiation (*n* = 3; Data were presented as means ± SD). g) Schematic of the phototherapy treatment process. h) Body weight changes and i) tumor growth curves during treatment. j) Tumor weights collected from different groups after 12 days of treatments. (As for Figure 7h–j, the statistical significance was assessed by Student's *t*‐test; *n* = 5; Data were presented as means ± SD, ^***^ *p* < 0.001). k) Representative photographs of mice in different treatment groups and the corresponding tumor images. l) Images of H&E, TUNEL, and Ki‐67 staining of tumor sections (Scale bar: 200 µm).

To determine the optimal treatment window, we monitored the tumor accumulation of BBTD@KZTPA NPs A in CT26 tumor‐bearing mice using an in vivo imaging system (**Figure** **7**a). Fluorescence intensity at the tumor site peaked at 3 h post‐injection before gradually declining (Figure 7e). Ex vivo imaging of organs and tumors at 3 and 50 h confirmed preferential nanoparticle accumulation in tumors (Figure 7b,d), demonstrating effective targeting. Importantly, it was found that the signal intensity was significantly stronger under the 760 nm wavelength condition (Figures S31 and S32, Supporting Information). Since BBTD NPs had a characteristic fluorescence emission peak at 820 nm (Figure 5f), this result suggests that the interaction between BBTD and KZTPA is stable under the tumor microenvironment.

Based on these findings and accounting for cellular internalization kinetics, we selected 6 and 24 h postinjection for phototherapy. Upon 808 nm laser irradiation (0.8 W cm^−2^, 5 min), tumors treated with BBTD@KZTPA NPs A at 6 h exhibited a rapid temperature rise (Δ*T* = 14.2 °C; Figure 7e,f), while the 24 h group showed a milder increase (Δ*T* = 9.5 °C; Figure S33, Supporting Information). Saline groups displayed minimal photothermal effect (Δ*T* = 4.4 °C).

Over the 12‐day treatment period, the body weight and tumor volume of all groups were measured every 3 days for 12 days, and at the end of the therapy, the tumors collected from each group were weighed and photographed. The growth inhibition of the tumors did not differ among the Saline, Saline + L, and BBTD@KZTPA NPs A groups. In contrast, the group of BBTD@KZTPA NPs A + L achieved near‐complete tumor suppression by only two injections (Figure 7i–k). This result could be attributed to the suitable combination of photodynamic and photothermal effects of BBTD@KZTPA NPs A and its longer retention in tumors, which not only improved the therapeutic efficiency but also reduced the harm to mice. Furthermore, the body weight increased stably among the groups during the 12 days treatment period (Figure 7h), indicating the negligible toxicity of BBTD@KZTPA NPs A treatment.

To underscore the therapeutic advantage of combining mild photothermal and strong photodynamic effects, we compared BBTD NPs under identical conditions. The results showed that the temperature at the tumor site reached 62.9 °C after irradiation for 5 min, and significant skin damage was observed after one round of treatment (Figure S34, Supporting Information). In contrast, BBTD@KZTPA NPs A maintained efficacy without adverse effects (Figure 7k), highlighting its balanced PTT/PDT synergy for safer therapy.

Hematoxylin and eosin (H&E) staining, terminal deoxynucleotidyl transferase‐mediated nick end labeling (TUNEL) assay, and Ki‐67 staining of tumor tissues revealed significant necrosis and apoptosis in the BBTD@KZTPA NPs A + L group, with minimal effects observed in control groups (Figure 7l). Additionally, H&E staining of major organs confirmed the absence of pathological damage or inflammatory lesions (Figure S35, Supporting Information), demonstrating both the potent antitumor efficacy and excellent biosafety of BBTD@KZTPA NPs A.

## 3 Conclusion

In conclusion, this work constructed three ACQ@AIE bimolecular systems and systematically elucidated how intermolecular interactions govern their optical properties. The bimolecular complexes were stabilized through electrostatic adsorption between conjugated structures, with reduced Δ*E_st_* arising from HOMO‐LUMO orbital separation that facilitated energy flow between S_1_ and T_1_. Crucially, structure size disparity dictated the aggregation behavior: smaller AIE molecules enhanced ACQ aggregation to promote non‐radiative transitions and heat generation, whereas larger AIE molecules stacked with ACQ molecules to suppress non‐radiative pathways while boosting ROS production. Optimal optical regulation occurred with matched molecular sizes due to favorable intermolecular interactions. By strategically regulating molar ratios, we achieved precise property control. The BBTD@KZTPA system – combining mild photothermal and strong photodynamic effects–was engineered into nanoparticles that demonstrated effective tumor accumulation, prolonged retention, and near‐complete tumor eradication after just two treatments within 12 days, alongside minimal systemic toxicity. Beyond advancing the mechanistic understanding of the structure size‐interaction‐property relationships, this work establishes a rational design framework for developing low‐toxicity and tunable high‐performance tumor phototherapy materials: by strategically harnessing intermolecular interactions, material properties could be precisely tuned by versatile and cost‐effective approach, which is hard to be achieved by single molecule materials.

## 4 Experimental Section

### Preparation of ACQ@AIE Bimolecular Systems

The DMF solution (3 mL) that contains molecules (0.5 µmol, with different ratios of ACQ and AIE molecule) was be added to water (7 mL), and the ACQ@AIE bimolecular systems were formed.

### Preparation of Nano Particles

BBTD‐loaded nanoparticles (BBTD NPs) and co‐assembled nanoparticles (BBTD@KZTPA NPs) were prepared via ultrasonication‐assisted nanoprecipitation. A THF solution (1 mL) containing BBTD (0.5 µmol) and BBTD@KZTPA (molar ratio 5:1) was injected dropwise (2 min) into aqueous phase (9 mL) containing DSPE‐mPEG_2000_ (30 mg) under continuous sonication. The mixture was further sonicated for 5 min followed by high‐intensity probe disruption (100 W, 6 mm tip, 2 min). Free components were removed by dialysis (MWCO 1 kDa, 24 h, RT) to obtain BBTD NPs and BBTD@KZTPA NPs A. For BBTD@KZTPA NPs B, the protocol was identical except for using 10 mg DSPE‐mPEG_2000_.

### Photothermal Effect of BBTD and BBTD@AIEs Bimolecular Systems

Solutions (2 mL) of BBTD (25 µm), BBTD@TPA3OMe (25 µM@25 µM), BBTD@KZTPA (25 µm@25 µm), BBTD@TKZTPA (25 µm@25 µm), and their individual components (TPA3OMe, KZTPA, TKZTPA; 25 µm each) in DMF/H₂O (f_w_ = 70%) were irradiated with 808 nm laser (0.6 W cm^−2^) respectively. As for the evaluation of the flexible adjustment, BBTD (1 mL, 50 µm), BBTD@KZTPA (1 mL, 50 µm, with a different molar ration of molecules), and BBTD@TKZTPA (1 mL, 50 µm, with a different molar ration of molecules) solutions were irradiated with 808 nm laser (0.6 W cm^−2^) respectively. Temperature was monitored at 1‐min intervals over 10 min using an infrared thermal camera. Blank control as DMF/H_2_O (f_w_ = 70%) solution was set. Data were presented as means ± SD (*n *= 3).

### Photodynamic Effect of BBTD and BBTD@AIEs Bimolecular Systems

The 1,3‐diphenylisobenzofuran (DPBF) probe assay was employed to quantify ROS production. To solutions of BBTD (2 mL, 25 µm), BBTD@TPA3OMe (2 mL, 25 µm@25 µm), BBTD@KZTPA (2 mL, 25 µm@25 µm), BBTD@TKZTPA (2 mL, 25 µm@25 µm)and their individual components (TPA3OMe, KZTPA, TKZTPA; 2 mL, 25 µm each) in DMF/H₂O (f_w_ = 70%) were irradiated with 808 nm laser (0.6 W cm^−2^) respectively, DPBF solution (30 µL, 1.0 mg mL^−1^, DMF) was added. Samples were irradiated with an 808 nm laser (0.6 W cm^−2^). The degradation kinetics of DPBF were monitored by measuring the UV–vis absorbance at 415 nm. A solvent control (DMF/H₂O, f_w_ = 70%) was analyzed in parallel to account for background interference.

### EPR Tests of BBTD and BBTD@AIEs Bimolecular Systems

ROS generated by BBTD and BBTD@AIEs were detected by EPR. The free radical trapping agents, 2,2,6,6‐tetramethylpiperidin (TEMP) and 5,5‐dimethyl‐1‐pyrroline N‐oxide (DMPO), were used to detect the signal of ROS. TEMP/DMPO were added to BBTD (2 mL, 25 µm), BBTD@TPA3OMe (2 mL, 25 µm@25 µm), BBTD@KZTPA (2 mL, 25 µm@25 µm), BBTD@TKZTPA (2 mL, 25 µm@25 µm) solutions, and after mixed up, samples (100 µL) were detected.

### Photothermal Effect of BBTD NPs and BBTD@KZTPA NPs

BBTD NPs (1 mL, 20 µm), BBTD@KZTPA NPs A (1 mL, 20 µm), and BBTD@KZTPA NPs B (1 mL, 20 µm) were irradiated with an 808 nm laser (0.8 W cm^−2^) for 10 min. Temperature was monitored at 1‐min intervals using an infrared thermal camera. Data were presented as means ± SD (*n* = 3).

### Photodynamic Effect of BBTD NPs and BBTD@KZTPA NPs

To water solutions of BBTD NPs (2 mL, 20 µm), BBTD@KZTPA NPs A (2 mL, 20 µm) and BBTD@KZTPA NPs B (2 mL, 20 µm), of DPBF solution (30 µL, 1.0 mg mL^−1^, DMF) was added. Samples were irradiated with an 808 nm laser (0.6 W, 0.8 and 1.2 W cm^−2^). The degradation kinetics of DPBF were monitored by measuring the UV–vis absorbance at 415 nm. A solvent control (water) was analyzed in parallel to account for background interference.

### Theoretical Calculation

DFT calculations were conducted using the Gaussian 09 program. MD simulations were carried out using xtb and GROMACS 2019.6. Energy decomposition analysis was carried out by utilizing the sobEDAw method to assess the intermolecular interaction.

### Cell Culture

CT26 cells were maintained in Roswell Park Memorial Institute (RPMI)‐1640 medium supplemented with 10% fetal bovine serum (FBS) and 1% penicillin‐streptomycin (P/S) under standard culture conditions (37 °C, 5% CO_2_). Cells were passaged at 80–90% confluence using 0.25% trypsin‐Ethtlene Diamine Tetraacetic Acid (EDTA) and subcultured every 48–72 h.

### Cytotoxicity Test

Cell Counting Kit‐8 (CCK‐8) was used to evaluate the vitro toxicity of BBTD@KZTPA NPs A for CT26 cells. For dark toxicity evaluation, CT26 cells were seeded in 96‐well plates at 1 × 10⁵ cells per well and allowed to adhere until the cell density reached ≈70%. Cells were then treated with BBTD@KZTPA NPs A (varying concentrations) in a complete medium for 12 h. After incubation, the medium was replaced with fresh medium containing CCK‐8 reagent (10 µL) and incubated for 1 h. Absorbance was measured at 450 nm using a microplate reader. As for light toxicity evaluation, following the same seeding protocol, CT26 cells were treated with BBTD@KZTPA NPs A (varying concentrations) for 12 h, then irradiated with an 808 nm laser (0.8 W cm^−2^, 5 min). Postirradiation, the medium was replaced with CCK‐8‐containing medium (10 µL), incubated for 1 h, and absorbance was measured at 450 nm using a microplate reader.

### Cell Uptake Assay

CT26 cells were seeded into 48‐well plates with a density of 2 × 10^5^ cells per well. When the cell density reached ≈70%, cells were incubated with BBTD@KZTPA NPs‐A (10 µm) for 2, 4, and 6 h. Then, the CT26 cells were washed with PBS, fixed with 4% paraformaldehyde (100 µL, 20 min, rt), and stained DAPI (100 µL, 2 µg mL^−1^, 10 min, dark). Finally, the coverslip was inverted on the slide dripped with antifade mounting medium, and observed with CLSM. (BBTD@KZTPA NPs A: E_x_ 670 nm; E_m_ 700–800 nm)

### Intracellular ROS Detection

2'7‐dichlorodihydrofluorescein diacetate (DCFH‐DA) probe was used to detect the ROS production of BBTD@KZTPA NPs A. The cells were subjected to four distinct treatment regimens to evaluate ROS production: 1) PBS control: Cells were incubated with PBS for 30 min followed by replacement with RPMI‐1640 medium (100 µL) containing 10 µM DCFH‐DA probe; 2) Laser‐only treatment: Following PBS incubation (30 min), cells were loaded with DCFH‐DA (10 µm in RPMI‐1640) and subsequently irradiated with an 808 nm laser (0.8 W cm⁻^2^) for 5 min; 3) BBTD@KZTPA NPs A‐only treatment: Cells were incubated with BBTD@KZTPA NPs A (10 µm) for 4 h before DCFH‐DA (10 µm) loading; 4) BBTD@KZTPA NPs A with Laser treatment: After 4 h incubation with BBTD@KZTPA NPs A (10 µm), cells were loaded with DCFH‐DA (10 µm) and immediately subjected to laser irradiation (808 nm, 0.8 W cm⁻^2^, 5 min). Following respective treatments, all groups were washed with PBS, and the coverslip was inverted on the slide dripped with antifade mounting medium to be observed by CLSM.

### Live‐Dead Cell Staining

CT26 cells were seeded into 48‐well plates with a density of 2 × 10^5^ cells per well. When the cell density reached ≈70%, the cells were divided into four treatment groups: 1) PBS; 2) PBS with 808 nm laser irradiation (0.8 W cm^−2^, 5 min); 3) BBTD@KZTPA NPs A (10 µm) incubation for 4 h; 4) BBTD@KZTPA NPs A incubation (10 µm) for 4 h with 808 nm laser irradiation (0.8 W cm^−2^, 5 min). Following treatments, all groups were maintained at 37 °C for an additional 1 h incubation period. Subsequently, cells were incubated with PBS (100 µL) containing propidium iodide (PI, 2 µm) and calcein‐AM (4.5 µm) for 20 min at 37 °C. After careful PBS washing, cellular fluorescence was immediately imaged by CLSM.

### Establishment of the CT26 Tumor‐Bearing Mice Model and In Vivo Phototherapy

Following acclimatization, mice were subcutaneously inoculated in the left hind limb with 100 µL of CT26 cell suspension (1 × 10^8^ cells mL^−1^). 5 days later, tumor growth was monitored until reaching ≈150 mm^3^ in volume, at which point 20 mice were randomly allocated into four treatment groups (*n* = 5 per group): 1) Control Group 1: Intravenous administration of saline (0.3 mL) on days 0 and 3; 2) Control Group 2: Saline injection (0.3 mL) followed by 808 nm laser irradiation (0.8 W cm^−2^) at 6 and 24 h postinjection, repeated on day 3; 3) Control Group 3: Intravenous injection of BBTD@KZTPA NPs A (100 µm, 0.3 mL) on days 0 and 3. 4) Experimental Group 4: BBTD@KZTPA NPs A injection (100 µm, 0.3 mL) followed by dual laser irradiation (808 nm, 0.8 W cm^−2^, 6 and 24 h postinjection), repeated on day 3. Every 3 days, the body weight and tumor volume of mice were recorded. Tumor volume was measured and calculated longitudinally with a vernier caliper for up to 12 days. The tumor volume was calculated according to the formula tumor volume = (tumor length) × (tumor width)^2^/2. All experiments requiring anesthesia for mice used isoflurane as an anesthetic drug.

### In Vivo Evaluation of Therapeutical Outcomes and Biosafety

On day 12, the mice were euthanized, and the tumors along with primary organs (heart, liver, spleen, lungs, and kidneys) were excised for histopathological evaluation. All tissues were fixed in 4% paraformaldehyde, embedded in paraffin, and sectioned into 5 µm slices. For tumor analysis, H&E staining was performed to assess overall tissue morphology, necrosis, and inflammatory infiltration, while TUNEL staining was used to detect apoptotic cells, and Ki‐67 immunohistochemical staining was conducted to evaluate cellular proliferation. For major organs, H&E staining was performed to examine potential treatment‐related pathological changes, including inflammation, degeneration, or necrosis, ensuring a comprehensive assessment of both therapeutic efficacy and systemic safety.

### Statistical Analysis

Data analyses were conducted using the Origin 2024b software. Data were reported as the mean ± standard deviation (SD). Student's *t*‐test was used to compare two independent samples. ^*^ *p* < 0.05, ^**^ *p* < 0.01, ^***^ *p* < 0.001. In all cases, *p* < 0.05 was considered statistically significant.

## Conflict of Interest

The authors declare no conflict of interest.

## Supporting information



Supporting Information

## Data Availability

Data are available from the corresponding author upon reasonable request. 2411165 (BBTD) contains the supplementary crystallographic data for this paper. These data can be obtained free of charge from The Cambridge Crystallographic Data Centre via www.ccdc.cam.ac.uk/structures/
