# Supplementary material for: Flexible Regulation of Optical Properties Based on Structure Size‐Driven Intermolecular Interactions for Phototherapy
Source: Adv Sci (Weinh). 2025 Apr 24;12(27):2501468. doi: 10.1002/advs.202501468 (PMC12279202; doi:10.1002/advs.202501468)
Supplement: Supplementary file 1 — Supporting Information [file ADVS-12-2501468-s001.docx]

Supporting Information

**Flexible Regulation of Optical Properties Based on Structure Size-Driven Intermolecular Interactions for Phototherapy**

*Zhichao Gong, Guangbo Kang, Yu Cao, Jiachen Pan, Xuejiao Rong, Xiaobing Du, Danping Zhang, He Huang,* and Shuxian Meng**

**Table of Contents**

S1. Materials and InstrumentationS2

S2. Synthesis and Preparation ProceduresS3

S3. X-ray Crystallographic StructureS17

S4. CharacterizationsS19

S5. Theoretical CalculationsS26

S6. In Vitro StudyS29

S7. In Vivo StudyS30

S8. ReferenceS34

S9. Atomic CoordinatesS35

**S1. Materials and** **Instrumentation**

All chemical reagents and solvents were procured from Aladdin, Greagent, Heowns, Meryer, and Tianjin Jiangtian Chemical Technology Co., Ltd., and used as received. Roswell Park Memorial Institute (RPMI) 1640 medium and penicillin-streptomycin solution (P/S) were supplied by Thermo Fisher Scientific. Phosphate-buffered saline (PBS), 0.25% trypsin-EDTA solution, Cell Counting Kit-8 (CCK-8), calcein/PI cell viability/cytotoxicity assay kit, fetal bovine serum (FBS), 4',6-diamidino-2-phenylindole (DAPI), 2',7'-dichlorodihydrofluorescein diacetate (DCFH-DA), and anti-fade mounting medium were obtained from Beijing Solarbio Science & Technology Co., Ltd.

Nuclear magnetic resonance (NMR) spectra (^1^H: 400 MHz and 600 MHZ; ^13^C: 150 MHz) were acquired using Bruker AVANCE III HD (Germany) and JEOL JNM-ECZ600R (Japan) spectrometers. Matrix-assisted laser desorption/ionization time-of-flight mass spectrometry (MALDI-TOF MS) was performed on a Bruker Autoflex TOF/TOF III (USA). Single-crystal X-ray diffraction (SC-XRD) data were collected on a Bruker D8 VENTURE TXS PHOTON 100 CMOS diffractometer (Germany) with CuKα (λ = 1.54178) at 193 K. Structure determination was carried out using SHELXT and refined with SHELXL via full-matrix least-squares minimization in OLEX2 v1.2. UV-Vis absorption spectra were recorded on a Shimadzu UV-1800 spectrophotometer (Japan), while fluorescence spectra were obtained using an Edinburgh Instruments FLS1000 spectrometer (UK). Photothermal effect was recorded by FLIR E6 thermal imaging camera (USA). Electron paramagnetic resonance (EPR) spectra were acquired on a JEOL JES-FA200 spectrometer (Japan). Transmission electron microscopy (TEM) was conducted on a Philips-FEI Tecnai 12 microscope (Netherlands) operating at 120 kV. Hydrodynamic diameter and zeta potential were determined by dynamic light scattering (DLS) by using a Malvern Zetasizer Nano ZS90 (UK). Confocal laser scanning microscopy (CLSM) imaging was performed on a Leica Stellaris 8 FALCON system (Germany). In vivo and ex vivo fluorescence imaging was captured using a PerkinElmer IVIS Spectrum system (USA). Quantitative fluorescence analysis was conducted with ImageJ software.

**S2. Synthesis and Preparation Procedures**

**S2.1. Synthesis of BBTD**

**Figure S1.** Synthetic route of BBTD.

**Step one:** 4-Methylbenzaldehyde (246.2 mg, 1.5 mmol) was dissolved in anhydrous dichloromethane (DCM, 100 mL) under an ice bath and degassed via argon purging for 30 min. 2-Methylpyrrole (267.7 mg, 3.3 mmol) and trifluoroacetic acid (0.2 mL, 2.60 mmol) were sequentially injected into the solution. The ice bath was removed, and the reaction proceeded under argon protection with light exclusion for 6 h with vigorous stirring. A solution of 2,3-dichloro-5,6-dicyanoquinone (DDQ, 340.5 mg, 1.5 mmol) in tetrahydrofuran (THF)/DCM (1:1 v/v, 16 mL) was slowly added via a pressure-equalizing dropping funnel. After 2 h of stirring, the mixture was re-cooled to 0 ^o^C. Ethyldiisopropylamine (DIPEA, 12.0 mL) was introduced, followed by dropwise addition of BF_3_·Et_2_O (12.0 mL) over 15 min. The reaction was stirred at 0°C for 30 min and then warmed to room temperature for 12 h. The crude product was washed with saturated NaHCO_3_ (3 × 50 mL) and brine (2 × 30 mL). The organic layer was dried over anhydrous Na_2_SO_4_, filtered, and concentrated under reduced pressure. Purification by flash column chromatography (silica gel, DCM/petroleum ether=1:3 v/v) yielded the target compound **BOD** (R_f_ = 0.2 ) as a red solid (60.1 mg, 20.0% yield). ^1^H NMR (400 MHz, CDCl_3_) δ 8.08 (d, *J* = 8.0 Hz, 2H, Ar-H), 7.50 (d, *J* = 8.0 Hz, 2H, Ar-H), 6.58 (s, 2H, pyrrole-H), 6.58 (d, 2H, pyrrole-H), 6.22 (d, 2H, pyrrole-H), 3.94 (s, 3H, pyrrole-CH_3_), 2.62 (s, 3H, -OCH_3_).

**Step two:** In a 50 mL argon-purged Schlenk tube, BOD (354.0 mg, 1.0 mmol) and 2,1,3-benzothiadiazole-4-carbaldehyde (492.0 mg, 3.0 mmol) were charged under inert atmosphere. The system was evacuated and backfilled with argon three times, followed by injection of anhydrous acetonitrile (13.0 mL) and DCM (2.5 mL). The mixture was stirred at room temperature until complete dissolution, after which acetic acid (0.2 mL) and piperidine (0.6 mL) were sequentially added as catalytic additives. The reaction was heated to 70 ^o^C under reflux for 12 h. Upon completion, the darkened reaction mixture was quenched by dilution with DCM (30 mL) and transferred to a 100 mL round-bottom flask. Solvent removal was performed via rotary evaporation until concentrated to ~10 mL. The residue was adsorbed onto silica gel (200-300 mesh) through controlled solvent evaporation and subjected to column chromatography using DCM as eluent. A target purple-black band (R_f_ = 0.2) was isolated and concentrated to dryness. Recrystallization from DCM/MeOH (3:1 v/v) by slow vapor diffusion afforded **BBTD** as needle-like crystals (278.3 mg, 42.9% yield).

^1^H NMR (600 MHz, CDCl_3_): δ 8.47 (d, *J* = 16.2 Hz, 2H, -CH=CH-), 8.17 (d, *J* = 8.3 Hz, 2H, Ar-H), 8.02 (d, *J* = 16.2 Hz, 2H, -CH=CH-), 7.98 (d, *J* = 8.8 Hz, 2H, Ar-H), 7.95 (d, *J* = 7.0 Hz, 2H, Ar-H), 7.95 (d, *J* = 7.0 Hz, 2H, Ar-H), 7.68 (t, *J* = 19.0 Hz, 2H, Ar-H), 7.63 (d, *J* = 8.2 Hz, 2H, Ar-H), 7.12 (d, 2H, pyrrole-H), 6.82 (d, 2H, pyrrole-H), 3.99 (s, 3H, -OCH_3_). ^13^C NMR (151 MHz, CDCl_3_): δ 166.40, 155.37, 155.31, 153.52, 138.62, 138.40, 136.60, 131.92, 131.52, 130.50, 129.91, 129.69, 129.64, 129.59, 127.16, 123.06, 121.73, 117.51, 52.50. MALDI-TOF-MS: *m*/*z* calcd for C_33_H_21_BF_2_N_6_O_2_S_2_ 646.12 [M]^+^; found: 646.34.

**S2.2. Synthesis of TPA3OMe**

**Figure S2.** Synthetic route of **TPA3OMe**.

In an argon-purged 50 mL Schlenk tube, para-iodoanisole (2.106 g, 9.0 mmol), para-aminoanisole (369.4 mg, 3.0 mmol), Cs_2_CO_3_ (1.956 g, 6.0 mmol) and CuI (114.0 mg, 0.6 mmol) were suspended in anhydrous tetraethyl orthosilicate (TEOS, 15.0 mL). The system was subjected to argon purging for 30 min and heated to 145 ^o^C under vigorous for 32 h under strict argon protection. The cooled reaction slurry was quenched with ethyl acetate (EA, 10 mL) and 95% aqueous ethanol (20 mL). A pre-adsorbent mixture [prepared by combining NH_4_F (10 g) with deionized water (150 mL), then adding silica gel (100-200 mesh, 50 g)] was introduced portion-wise. After 5 h, the suspension was filtered and washed with EA (3 × 15 mL). The combined filtrates were concentrated by rotary evaporation. Purification was performed via column chromatography (silica gel 200-300 mesh) with PE/DCM (1:2 v/v) as eluent, monitoring by TLC. The target band (R_f_=0.3) was isolated and recrystallized from DCM/MeOH (1:3 v/v), yielding **TPA3OMe** as white needle crystals (455.7 mg, 45.3% yield).

^1^H NMR (600 MHz, CDCl_3_): δ 7.88 (d, *J* = 13.4 Hz, 6H, Ar-H), 7.01 (d, *J* = 13.4 Hz, 6H, Ar-H), 3.89 (s, 9H, -OCH_3_).

**S2.3. Synthesis of KZTPA**

**Figure S3.** Synthetic route of **KZTPA**.

**Step one:** In a 250 mL argon-purged round-bottom flask, bis(pinacolato)diboron (3.047 g, 12.0 mmol), Pd(dppf)Cl_2_ (377.7 mg, 0.52 mmol), potassium acetate (1.766 g, 18.0 mmol), and 4-bromo-N,N-bis(4-methoxyphenyl)aniline (2.306 g, 6.0 mmol) were dissolved in anhydrous 1,4-dioxane (130 mL). The solution was argon-purged for 30 min prior to refluxing at 100 ^o^C under static argon for 16 h. The cooled mixture was concentrated via rotary evaporation and purified by column chromatography (silica gel, 200-300 mesh) using petroleum ether/ethyl acetate (10:1 v/v). The target fraction (R_f_ = 0.3) was isolated and dried in vacuo, yielding **TPABpin** as a white powder (1.960 g, 75.7%).

^1^H NMR (600 MHz, CDCl_3_): δ 7.59 (d, *J* = 13.0 Hz, 2H, Ar-H), 7.06 (d, *J* = 13.6 Hz, 4H, Ar-H), 6.86 (d, *J* = 13.0 Hz, 2H, Ar-H), 6.83 (d, *J* = 13.6 Hz, 4H, Ar-H), 1.35 (s, 12H).

**Step two:** In a 50 mL Schlenk tube, 3,6-dibromo-9-(4-methoxyphenyl)-9H-carbazole (431.1 mg, 1.0 mmol), TPABpin (905.8 mg, 2.7 mmol), K₂CO₃ (276.4 mg, 2.0 mmol), and Pd(PPh_3_)_4_ (173.5 mg, 0.15 mmol) were dissolved in degassed 1,4-dioxane/H_2_O (5:1 v/v, 18 mL). The mixture was argon- purged for 30 min and heated at 90 ^o^C under argon for 12 h. After solvent removal by rotary evaporation, the residue was purified via column chromatography (petroleum ether/ethyl acetate = 10:1). The major band (R_f_ = 0.2) was collected, yielding **KZTPA** as a white powder (220.1 mg, 25.1%).

^1^H NMR (600 MHz, CDCl_3_): δ 8.32 (s, 2H, Ar-H), 7.60 (d, *J* = 8.5 Hz, 2H, Ar-H), 7.53 (d, *J* = 8.8 Hz, 4H, Ar-H), 7.49 (d, *J* = 8.9 Hz, 2H, Ar-H), 7.36 (d, *J* = 8.5 Hz, 2H, Ar-H), 7.13 (d, *J* = 8.9 Hz, 2H, Ar-H), 7.10 (d, *J* = 8.9 Hz, 8H, Ar-H), 7.04 (d, *J* = 8.6 Hz, 4H, Ar-H), 6.85 (d, *J* = 9.2 Hz, 8H, Ar-H), 3.93 (s, 3H, -OCH_3_), 3.81 (s, 12H, -OCH_3_). ^13^C NMR (151 MHz, CDCl_3_): δ158.95, 155.82, 147.59, 141.29, 140.98, 134.42, 133.17, 130.46, 128.49, 127.80, 126.47, 125.22, 123.91, 121.50, 118.23, 115.21, 114.80, 110.10, 55.72, 55.62. MALDI-TOF-MS: *m*/*z* calcd for C_59_H_49_N_3_O_5_ 879.37; found: 879.45 [M]^+^.

**S2.4. Synthesis of TKZTPA**

**Figure S4.** Synthetic route of TKZTPA.

**Step one:** A mixture of N,N-bis(4-methoxyphenyl)aniline (1.987 g, 6.5 mmol) and N-iodosuccinimide (NIS) (1.620 g, 7.2 mmol) in N,N-dimethylformamide (DMF, 20 mL) was stirred at 25 ^o^C for 24 h under ambient conditions. The reaction mixture was diluted with ethyl acetate (EA, 100 mL) and washed sequentially with saturated brine (2 × 50 mL) and deionized water (1 × 50 mL). The organic phase was dried over anhydrous Na_2_SO_4_, filtered, and concentrated in vacuo. Recrystallization from DCM/MeOH (3:1 v/v) afforded **TPAI** as off-white crystals (1.612 g, 56.6%).

^1^H NMR (600 MHz, DMSO-*d*_6_): δ 7.45 (d, *J* = 13.4 Hz, 2H, Ar-H), 7.03 (d, *J* = 13.4 Hz, 4H, Ar-H), 6.91 (d, *J* = 13.6 Hz, 4H, Ar-H), 6.55 (d, *J* = 13.4 Hz, 2H, Ar-H), 3.73 (s, 6H, -OCH_3_).

**Step two:**In a 50 mL Schlenk tube, 3,6-dibromocarbazole (325.0 mg, 1.0 mmol), TPABpin (905.8 mg, 2.7 mmol), K_2_CO_3_ (276.4 mg, 2.0 mmol), and Pd(PPh_3_)_4_ (173.5 mg, 0.15 mmol) were dissolved in degassed 1,4-dioxane/H_2_O (5:1 v/v, 18 mL). The mixture was argon-purged for 30 min and heated at 90 ^o^C under argon for 12 h. After solvent removal by rotary evaporation, purification via column chromatography (silica gel, PE/EA = 12:1) yielded **HKZTPA** (R_f_ = 0.15) as a white powder (317.6 mg, 41.0%).

^1^H NMR (600 MHz, DMSO-*d*_6_): δ 11.27 (s, 1H, -NH-), 8.44 (s, 2H, Ar-H), 7.64 (d, *J* = 6.3 Hz, 2H, Ar-H), 7.60 (d, *J* = 6.6 Hz, 4H, Ar-H), 7.50 (d, *J* = 6.2 Hz, 2H, Ar-H), 7.04 (d, *J* = 6.6 Hz, 8H, Ar-H), 6.92 (d, *J* = 6.7 Hz, 8H, Ar-H), 6.90 (d, *J* = 6.4 Hz, 4H, Ar-H), 3.75 (s, 12H, -OCH_3_).

**Step three:** In a 50 mL Schlenk tube, HKZTPA (386.5 mg, 0.5 mmol), TPAI (259.2 mg, 0.6 mmol), CuI (66.7 mg, 0.35 mmol), 1,10-phenanthroline (63.1 mg, 0.35 mmol), and KOH (196.4 mg, 0.35 mmol) were dissolved in anhydrous m-xylene (15 mL). The mixture was argon-purged for 30 min and heated at 140 ^o^C under static argon for 24 h. After solvent removal by rotary evaporation, the residue was purified by column chromatography (silica gel, PE/EA = 15:1 v/v). The target fraction (R_f_ = 0.2) was collected and recrystallized from DCM/MeOH (1:3 v/v), yielding **TKZTPA** as white needle-like crystals (298.6 mg, 55.4%).

^1^H NMR (600 MHz, DMSO-*d*_6_): δ 8.54 (d, *J* = 14.8 Hz, 2H, Ar-H), 7.67 (d, *J* = 6.5 Hz, 2H, Ar-H), 7.62 (d, *J* = 6.5 Hz, 4H, Ar-H), 7.42 (d, *J* = 6.6 Hz, 2H, Ar-H), 7.38 (d, *J* = 6.3 Hz, 2H, Ar-H), 7.18 (d, *J* = 6.6 Hz, 4H, Ar-H), 7.04 (d, *J* = 6.6 Hz, 8H, Ar-H), 6.98 (d, *J* = 6.7 Hz, 4H, Ar-H), 6.92 (d, *J* = 6.6 Hz, 8H, Ar-H), 6.90 (d, *J* = 6.6 Hz, 4H, Ar-H), 6.86 (d, *J* = 6.4 Hz, 2H, Ar-H), 3.74 (s, 18H, Ar-CH_3_). ^13^C NMR (151 MHz, CDCl_3_): δ 156.36, 155.84, 148.23, 147.56, 141.27, 140z`.87, 140.72, 134.47, 133.07, 129.54, 127.78, 127.65, 127.10, 126.47, 125.13, 123.89, 121.52, 120.72, 118.20, 115.00, 114.79, 110.27, 55.61. MALDI-TOF-MS: *m*/*z* calcd for C_72_H_60_N_4_O_6_ 1076.45; found: 1077.37 [M]^+^.

**S2.5. Preparation of BBTD single crystals**

In a 20 mL glass vial, BBTD (10 mg) was dissolved in dichloromethane (3 mL). MeOH (12 mL) was slowly layered atop the solution to establish a diffusion gradient. The vial was sealed and stored in darkness for 7 days, yielding purple-black rod-shaped crystals at the liquid interface.

**S2.6. NMR and MS Spectra**


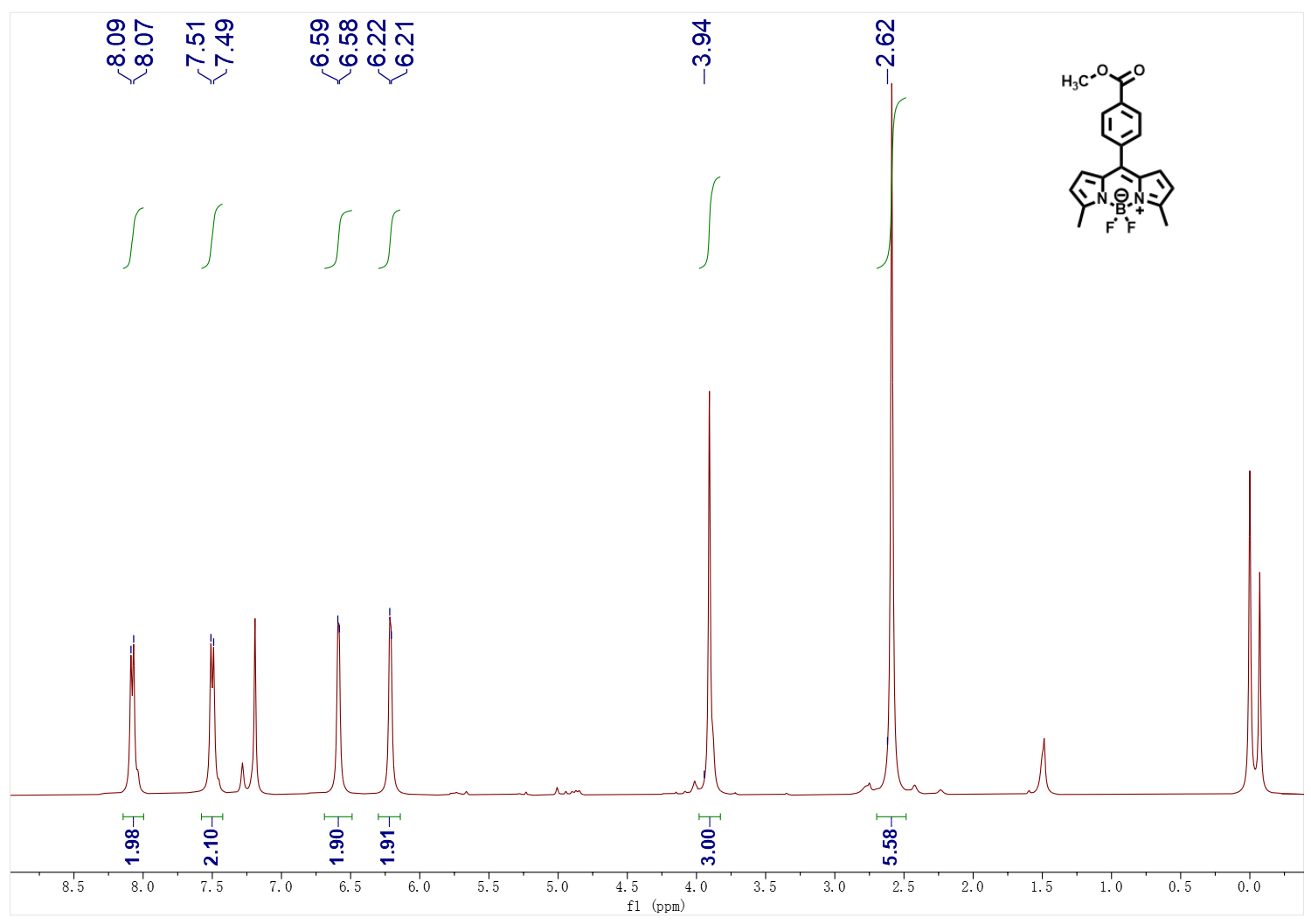


**Figure S5.** The ^1^H NMR spectrum of BOD (CDCl_3_, 400 MHz)


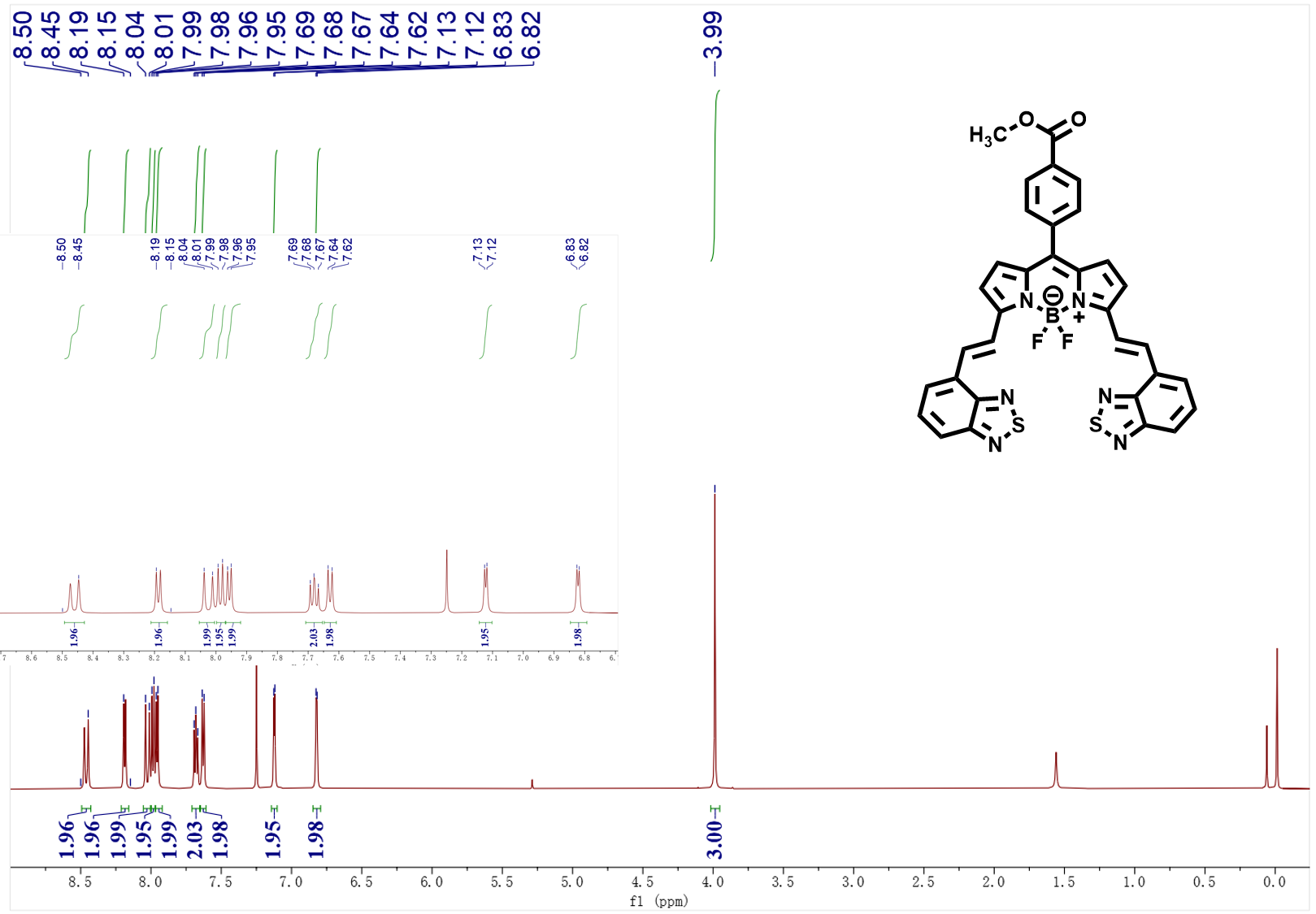


**Figure S6.** The ^1^H NMR spectrum of BBTD (CDCl_3_, 600 MHz).


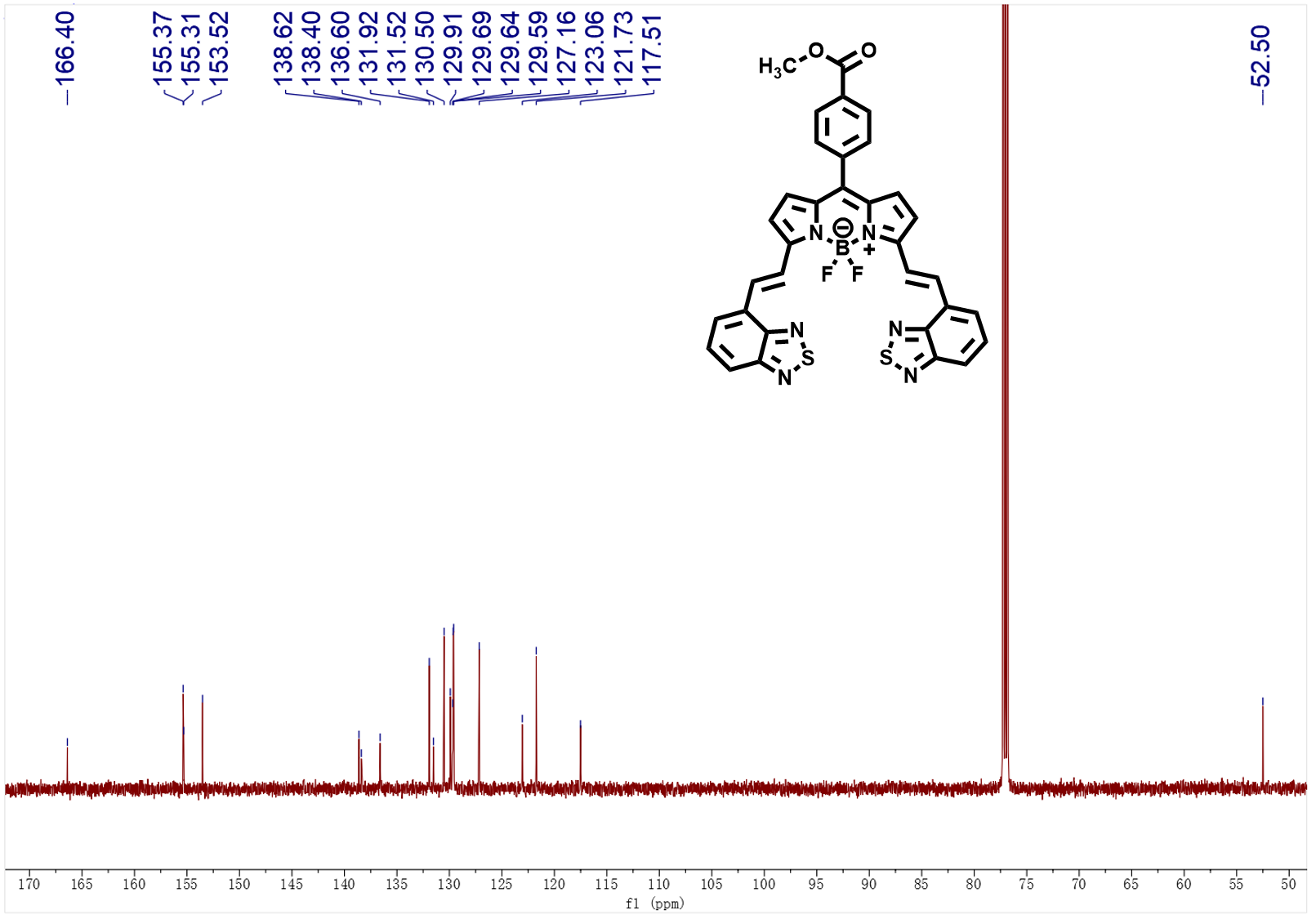


**Figure S7.** The ^13^C NMR spectrum of BBTD (CDCl_3_, 151 MHz).


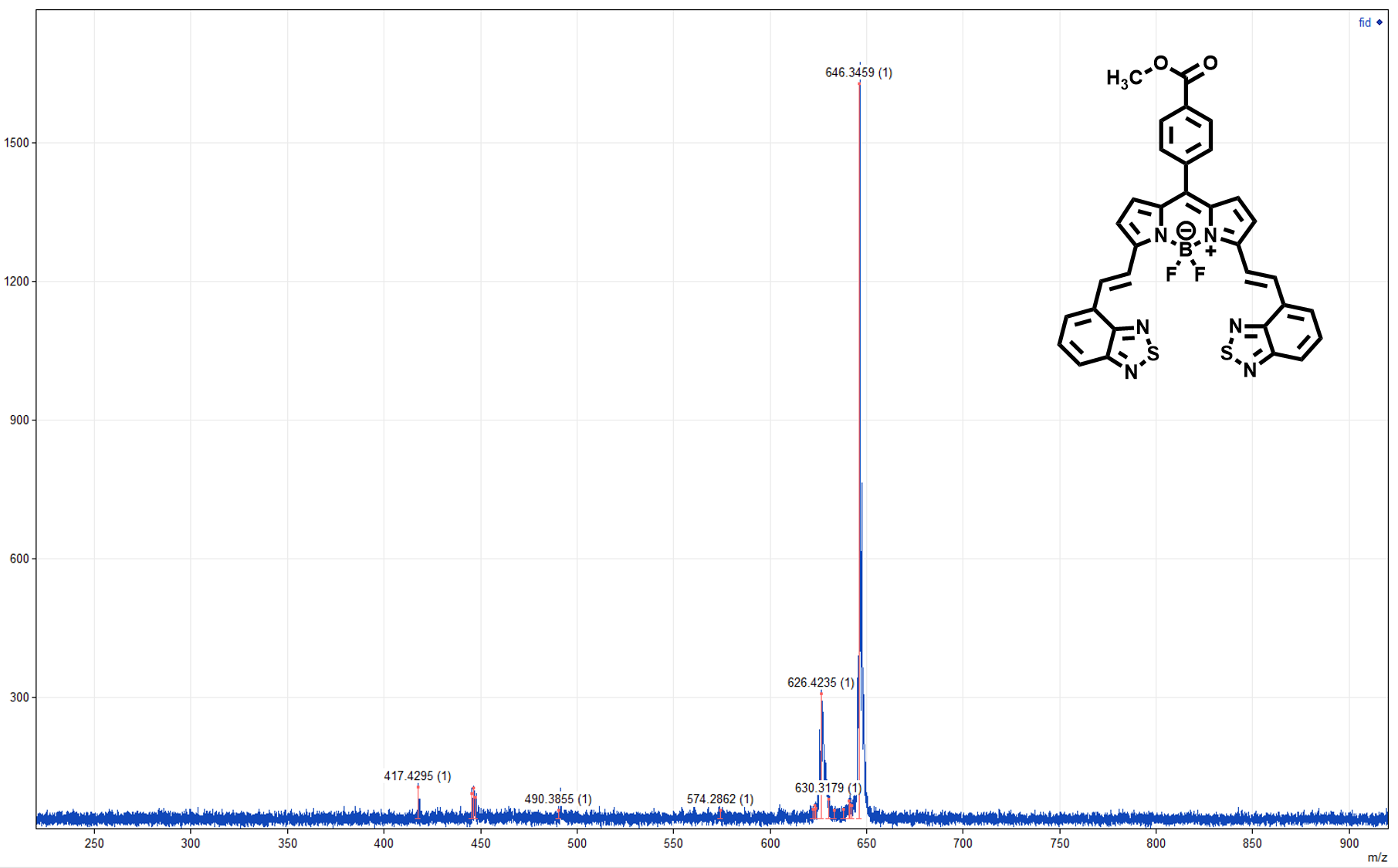


**Figure S8.** ESI-Q-MS spectra of BBTD.


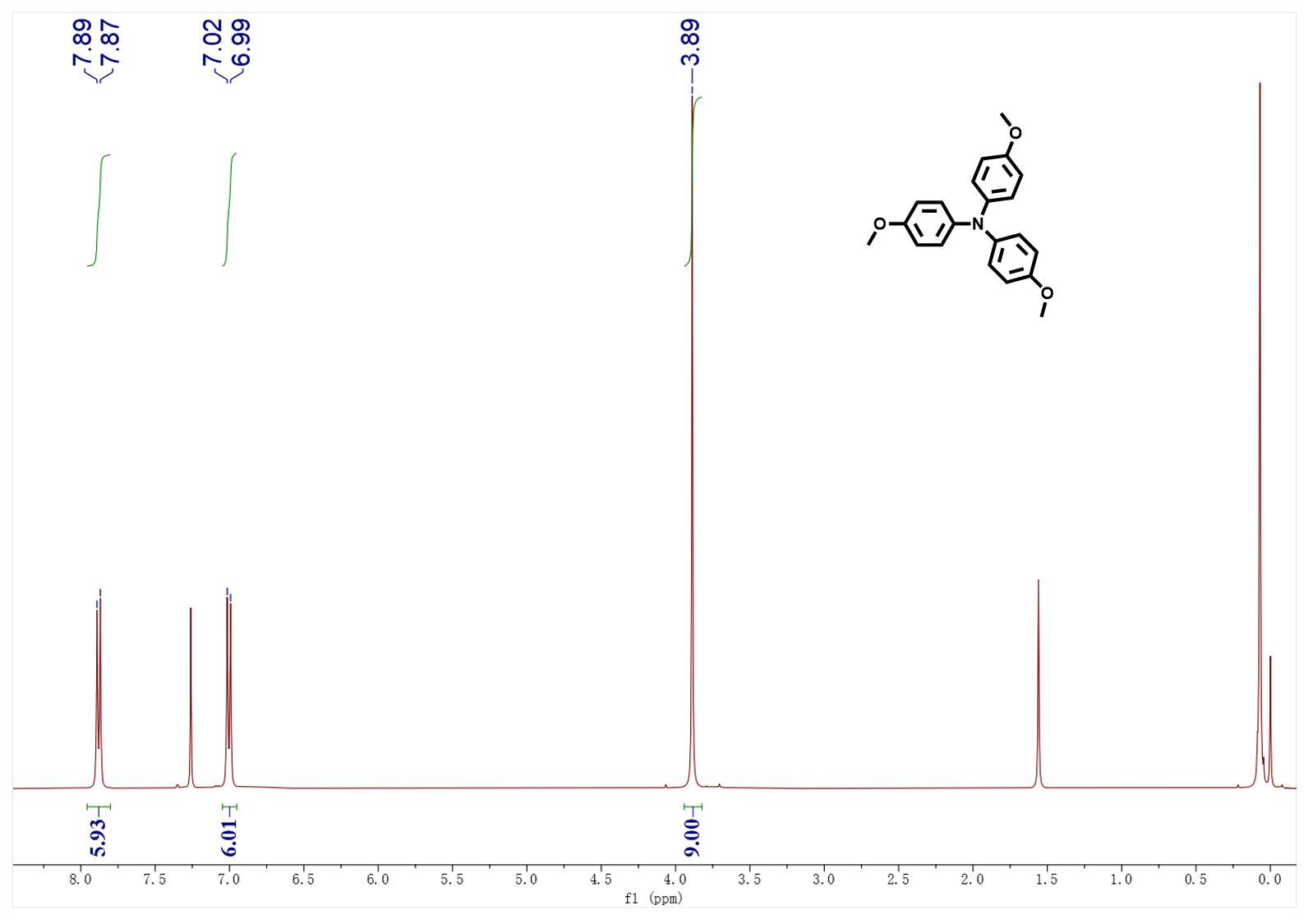


**Figure S9.** The ^1^H NMR spectrum of TPA3OMe (CDCl_3_, 600 MHz).


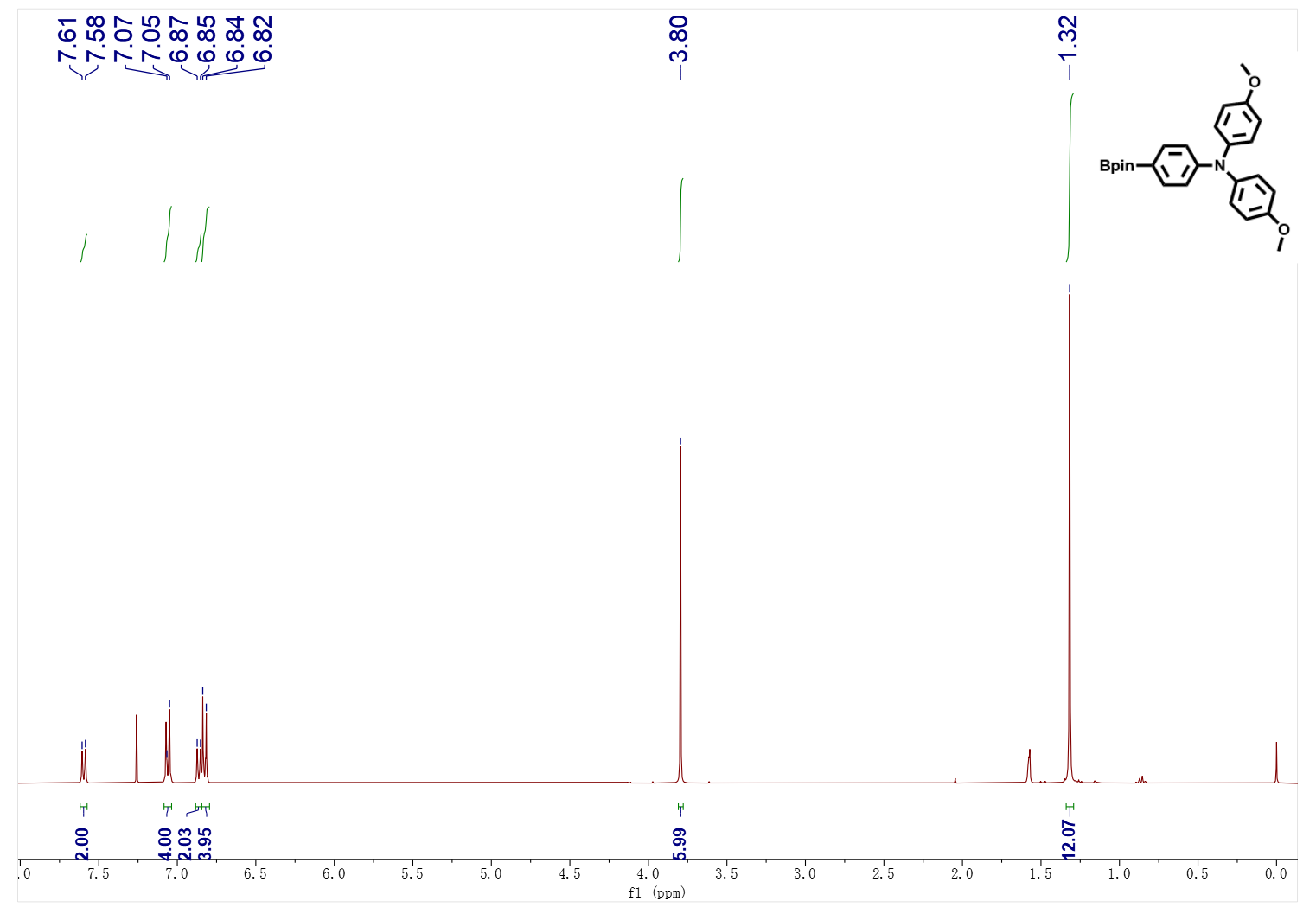


**Figure S10.** The ^1^H NMR spectrum of TPABpin (CDCl_3_, 600 MHz).


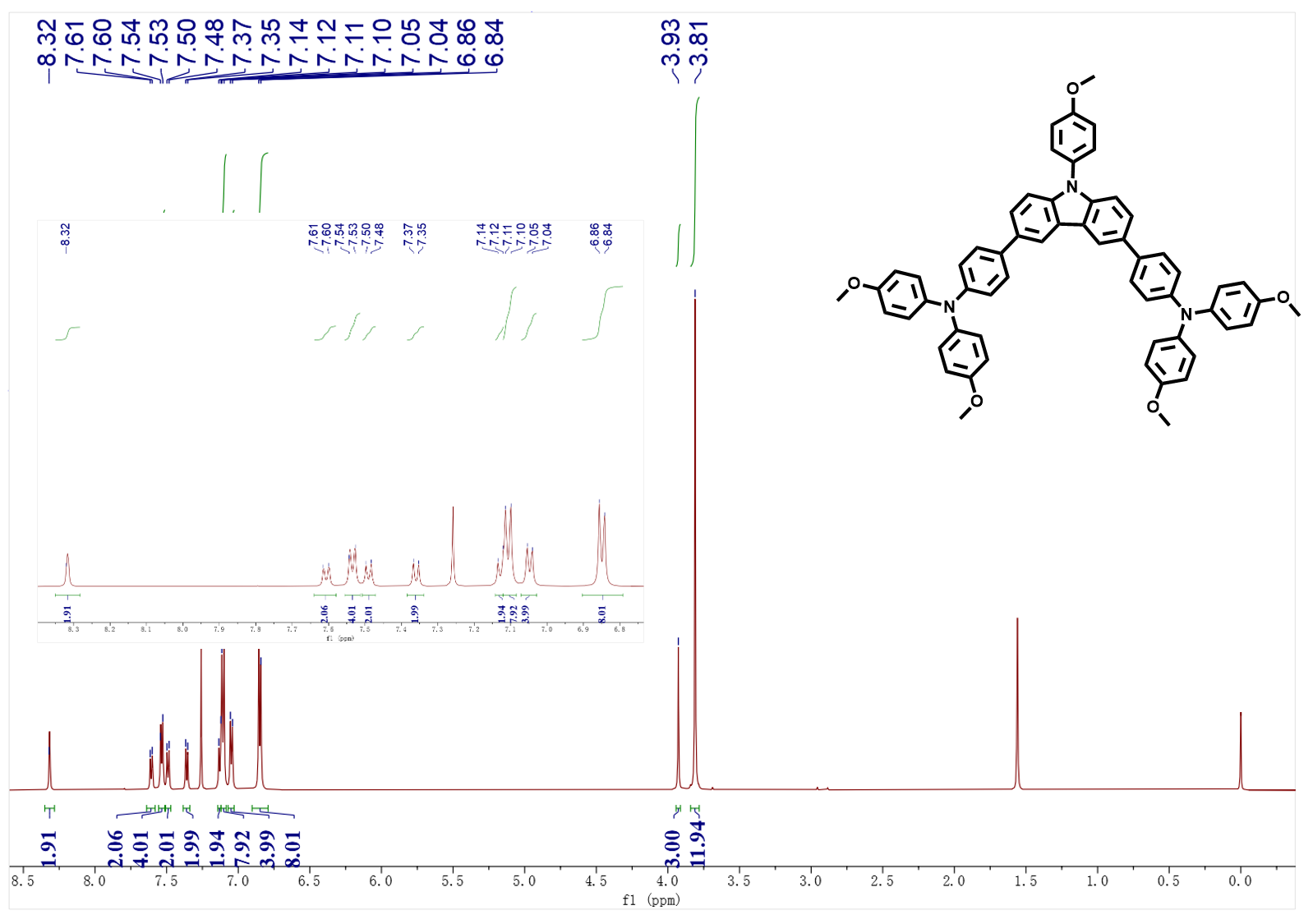


**Figure S11.** The ^1^H NMR spectrum of KZTPA (CDCl_3_, 600 MHz).


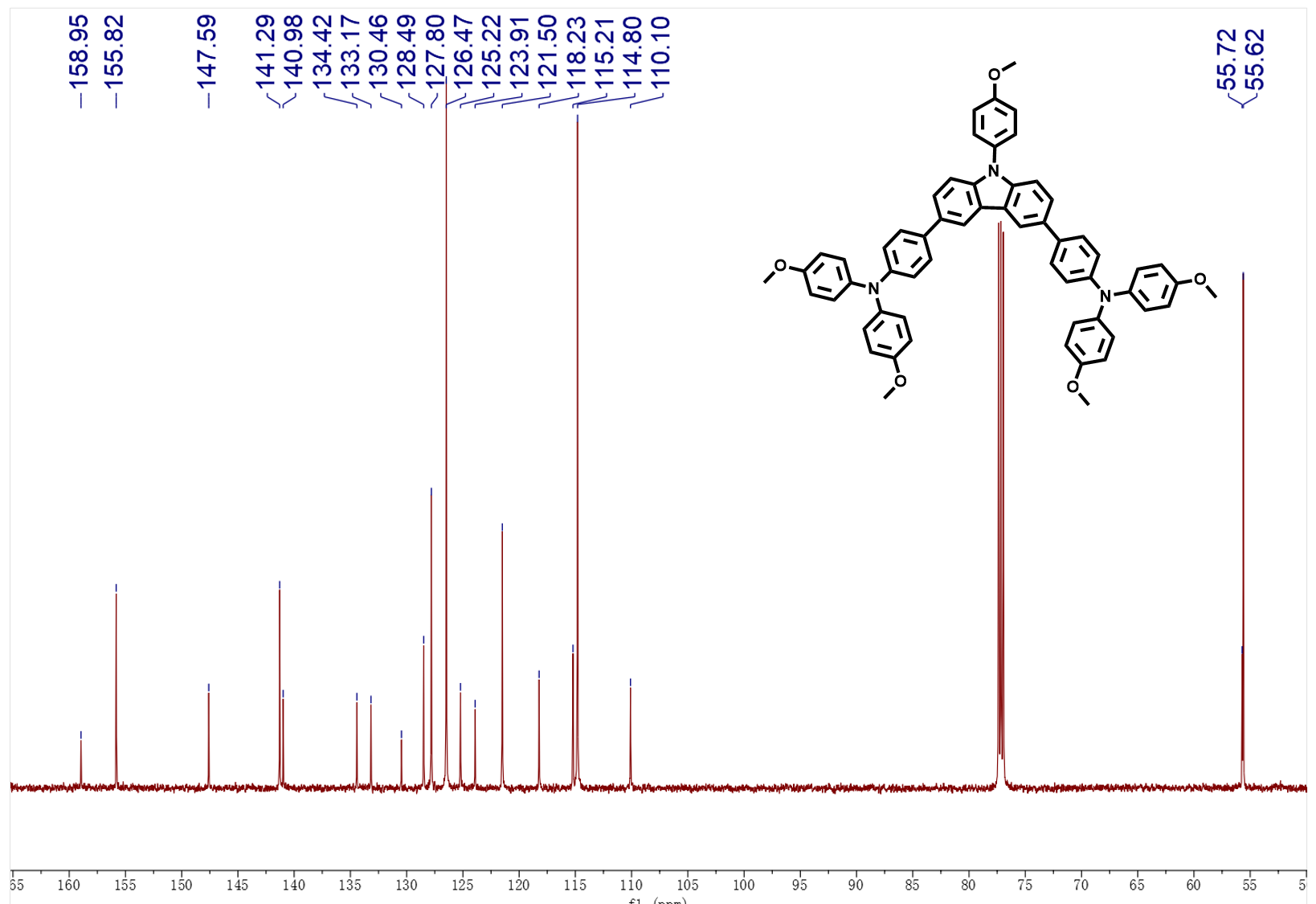


**Figure S12** The ^13^C NMR spectrum of KZTPA (CDCl_3_, 151 MHz).


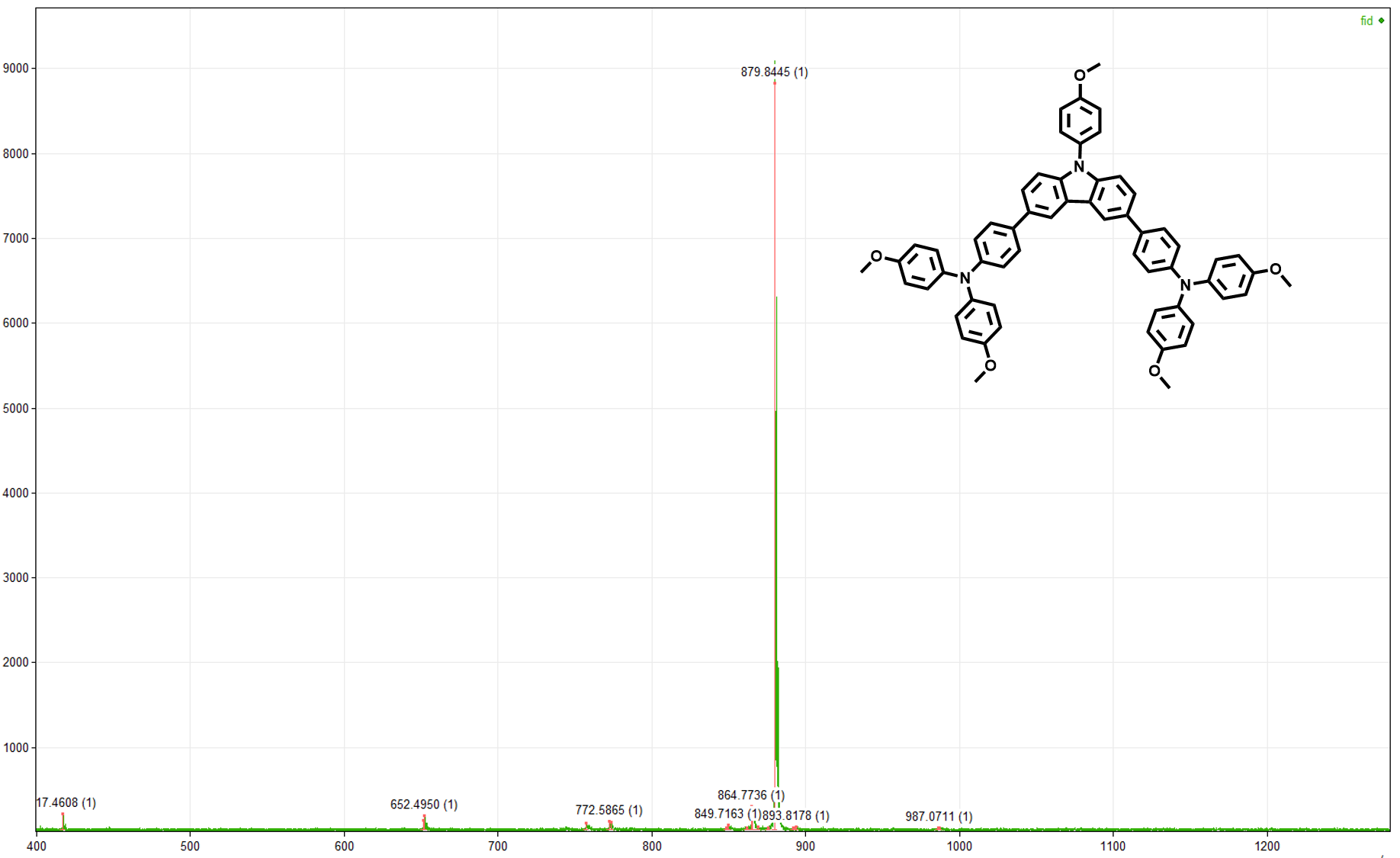


**Figure S13.** ESI-Q-MS spectra of KZTPA.


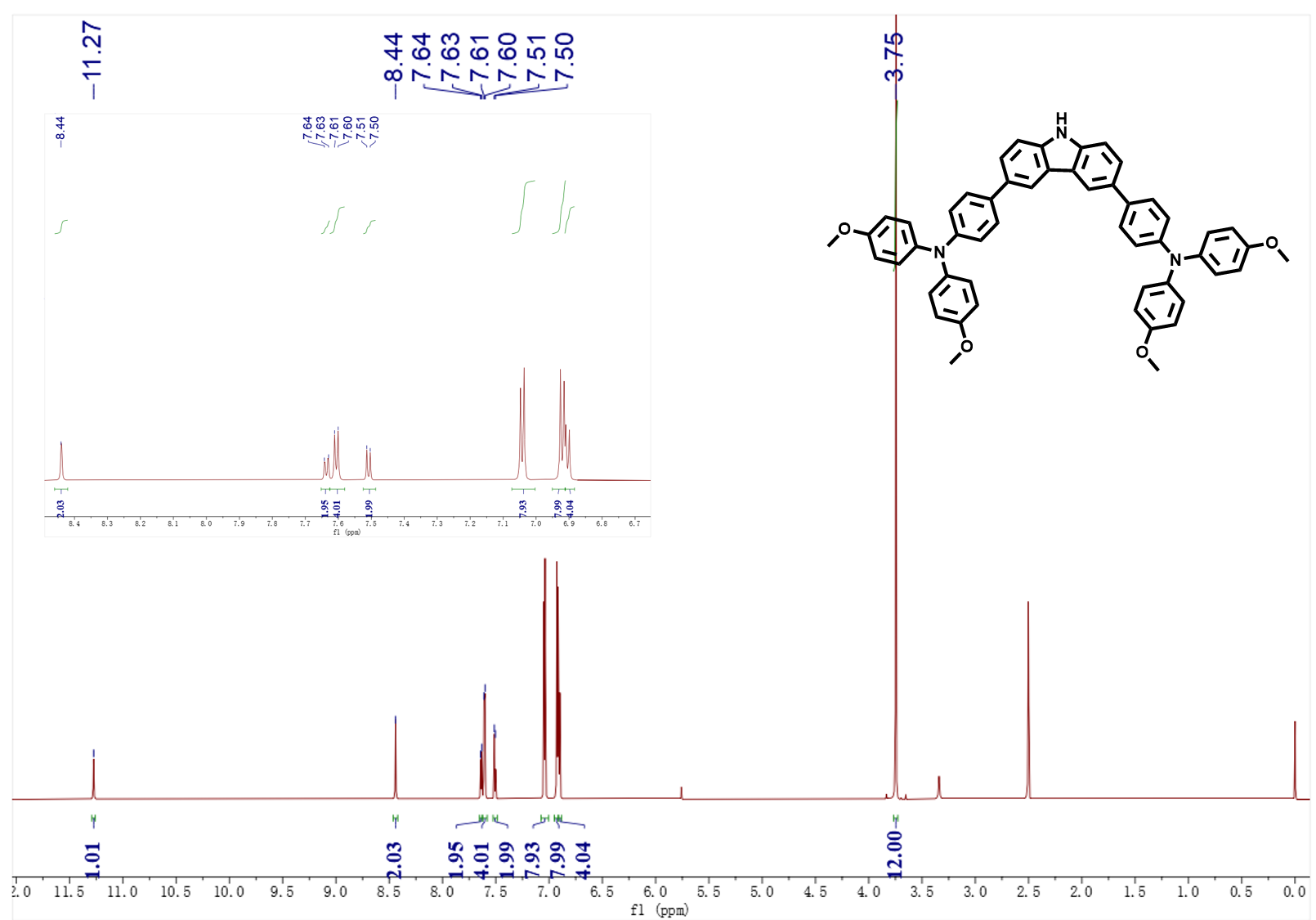


**Figure S14.** The ^1^H NMR spectrum of HKZTPA (DMSO-*d*_6_, 600 MHz).


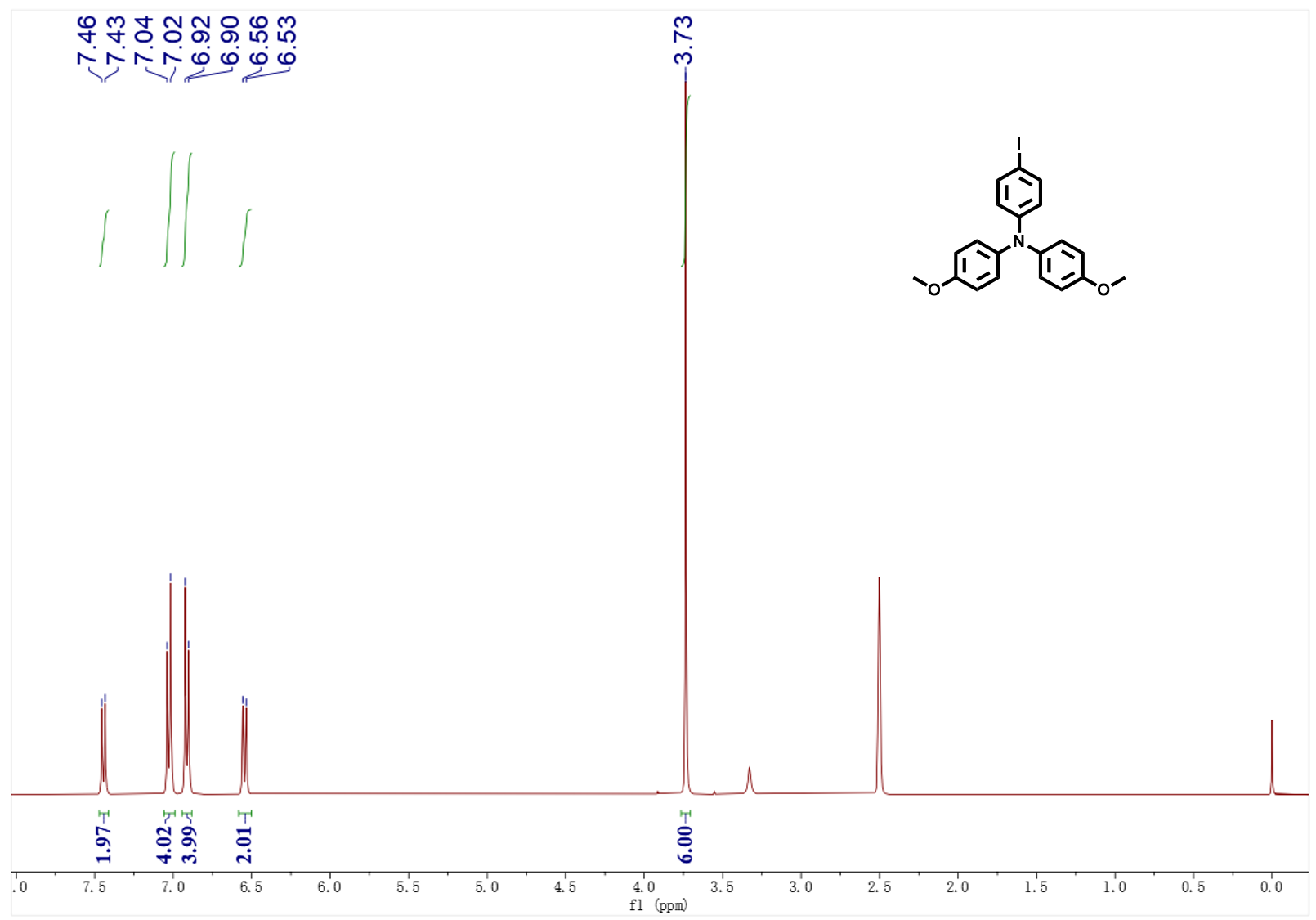


**Figure S15.** The ^1^H NMR spectrum of TPAI (DMSO-*d*_6_, 600 MHz).


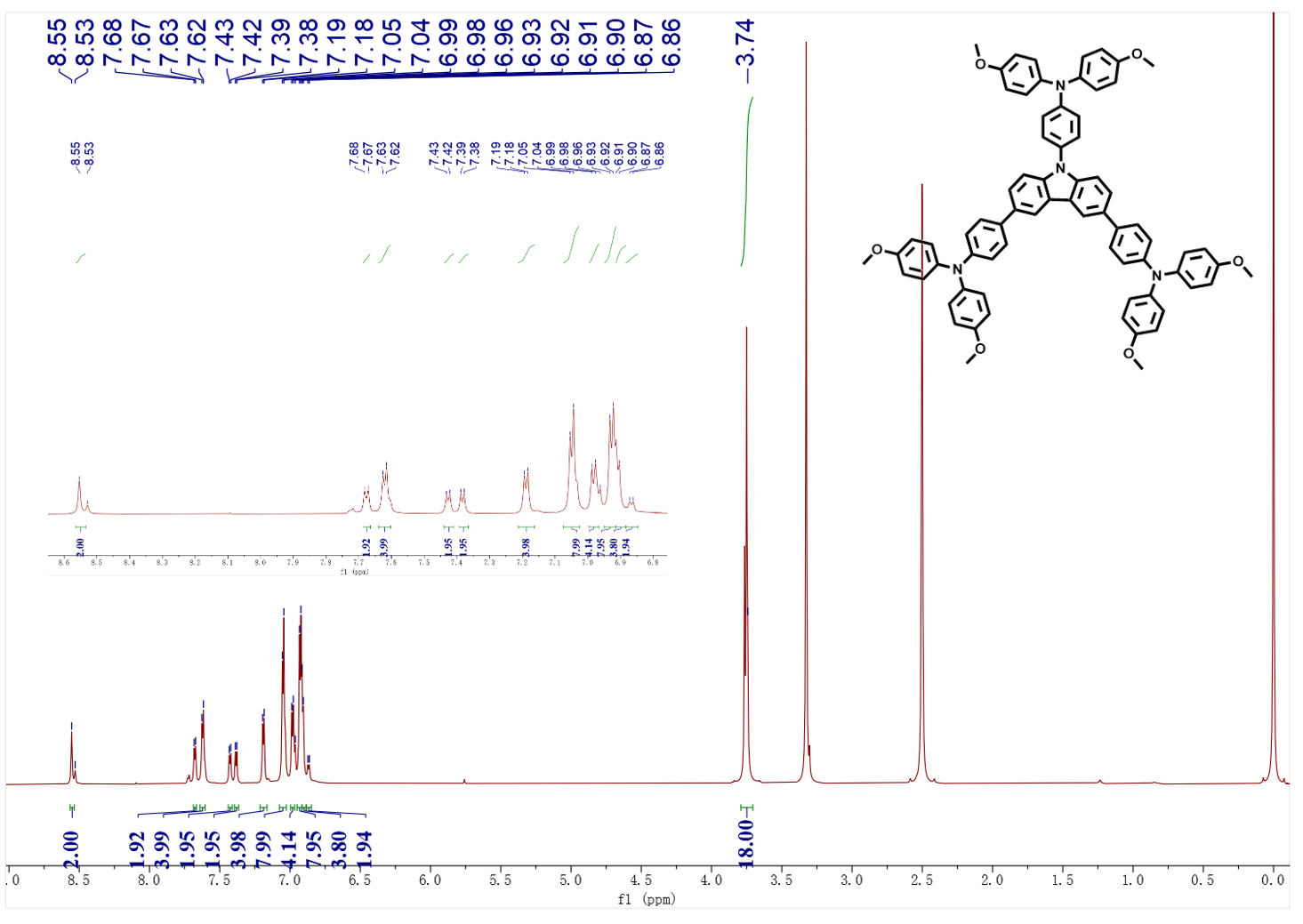


**Figure S16.** The ^1^H NMR spectrum of TKZTPA (DMSO-*d*_6_, 600 MHz).


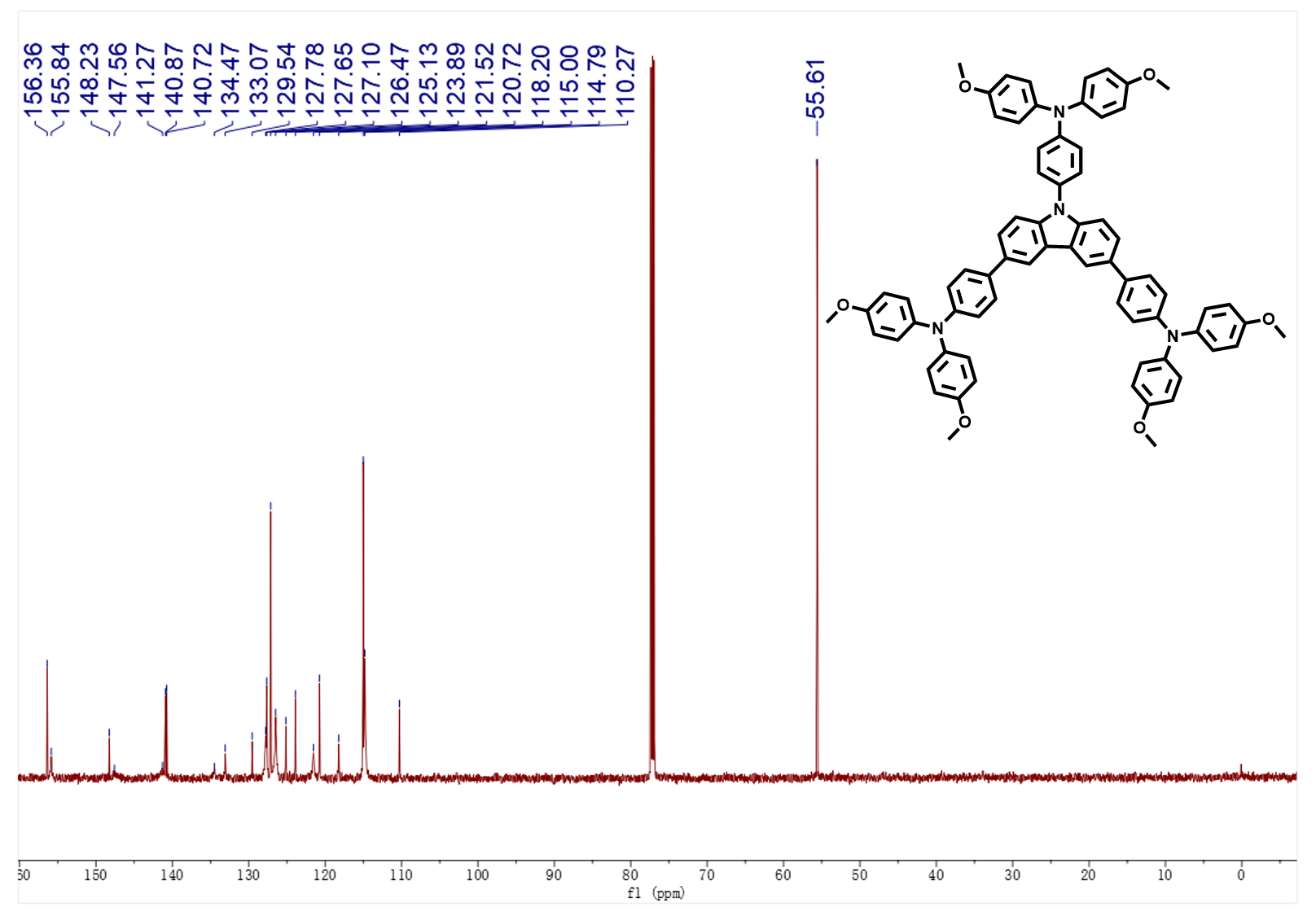


**Figure S17.** The ^13^C NMR spectrum of TKZTPA (CDCl_3_, 151 MHz).


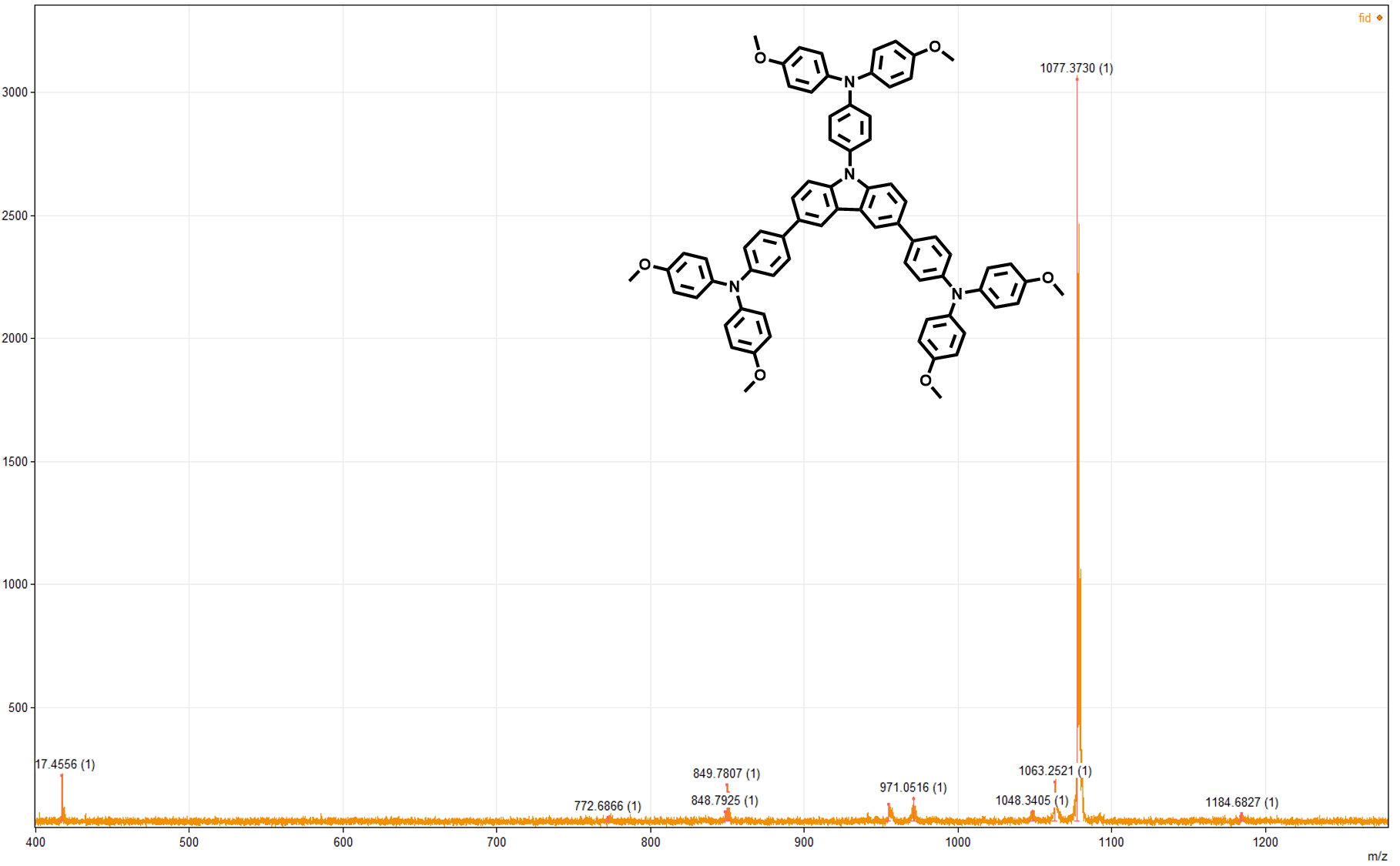


**Figure S18.** ESI-Q-MS spectra of TKZTPA.

**S3. X-ray Crystallographic Structure**

Single-crystal analysis reveals coexisting slipped J-aggregate stacking and face-centered H-aggregate motifs in the solid state. Although there is a difference between the molecular conformation and the crystal structure under solution conditions, it can be stated that the intermolecular conformation tends to converge more towards the H-aggregate as the intermolecular distance continues to decrease.


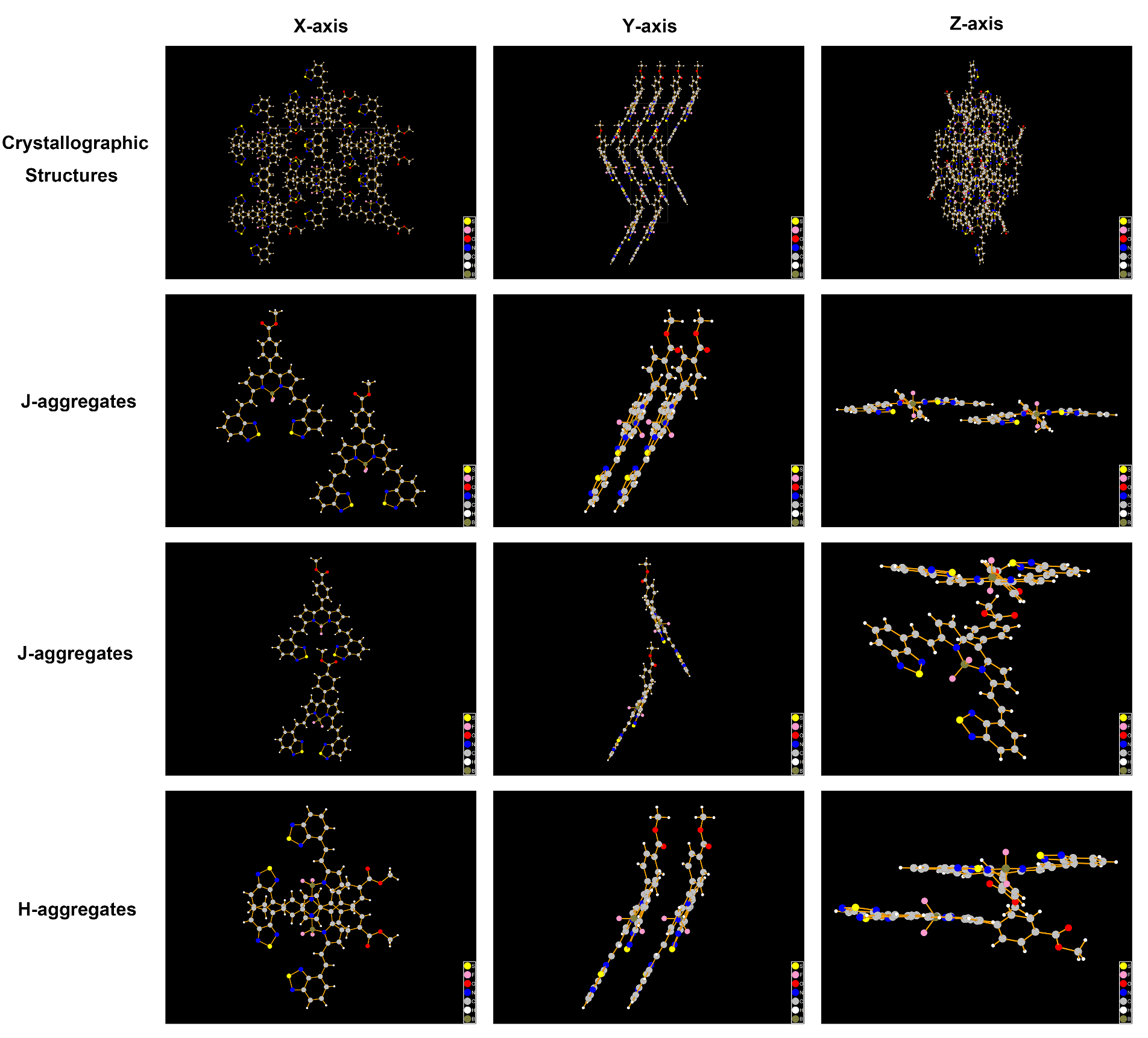


**Figure S19.** X-ray crystal structure of BBTD and different intermolecular stacking modes present in it.

**Table S1.** Crystallographic data for BBTD

| Compound | BBTD |
| --- | --- |
| Empirical formula | C_33_H_21_BF_2_N_6_O_2_S_2_ |
| Formula weight | 646.49 |
| Temperature/K | 193.00 |
| Crystal system | Orthorhombic |
| Space group | *Pna2_1_* |
| a, b, c/Å | 7.7219(3), 15.6633(6), 24.5051(8) |
| α, β, γ/° | 90, 90, 90 |
| Volume/Å^3^ | 2963.90(19) |
| Z | 4 |
| ρ_calc_/g cm^−3^ | 1.449 |
| μ/mm^‑1^ | 2.106 |
| F(000) | 1328.0 |
| Crystal size/mm^3^ | 0.12 × 0.1 × 0.08 |
| Radiation | CuKα (λ = 1.54178) |
| 2θ range for data collection/° | 6.698 to 149.178 |
| Index ranges | -9 ≤ h ≤ 9, -18 ≤ k ≤19, -30 ≤ l ≤ 26 |
| Reflections collected | 26698 |
| Independent reflections | 5780 [R_int_ = 0.0798, R_sigma_ = 0.0620] |
| Data/restraints/parameters | 5780/362/508 |
| Goodness-of-fit on F^2^ | 1.055 |
| Final R indexes [I>=2σ (I)] | R_1_ = 0.0414, wR_2_ = 0.0993 |
| Final R indexes [all data] | R_1_ = 0.0520, wR_2_ = 0.1056 |
| Largest diff. peak/hole / e Å^-3^ | 0.17/-0.32 |
| Flack parameter | 0.045(12) |

**S4. Characterizations**

**S4.1. The ACQ effect of BBTD and AIE effect of KZTPA and TKZTPA**


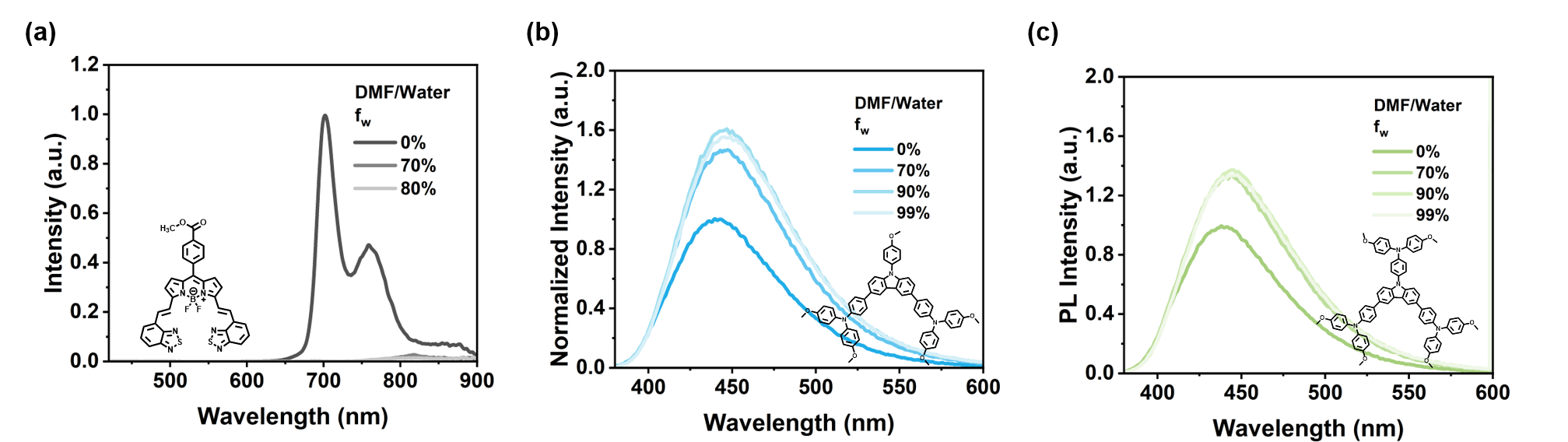


**Figure S20.** Change of fluorescence emission intensity with the increase of water fraction in DMF (Concentration: 10 μM).

**S4.2. Measurement of UV-Vis Absorbance of BBTD and BBTD@AIEs Bimolecular Systems**

2 mL BBTD (25 μM), BBTD@TPA3OMe (25 μM@25 μM), BBTD@KZTPA (25 μM@25 μM), BBTD@TKZTPA (25 μM@25 μM) DMF/Water solutions with different water fraction (f_w_) were prepared in DMF/water mixtures with systematically varied water fractions (f_w_ = 0%-90%). Absorbance spectra (400-1000 nm) were recorded using a 1 cm quartz cuvette.

As for the evaluation of the flexible adjustment, 1 mL BBTD (50 μM), BBTD@KZTPA (50 μM, with different molar ration of molecules) and BBTD@TKZTPA (50 μM, with different molar ration of molecules) solutions (DMF/Water, f_w_=70%) were added in a quartz cuvette, and the absorbance in the range of 400-1000 nm were measured. Absorbance profiles were presented in Figure S21.


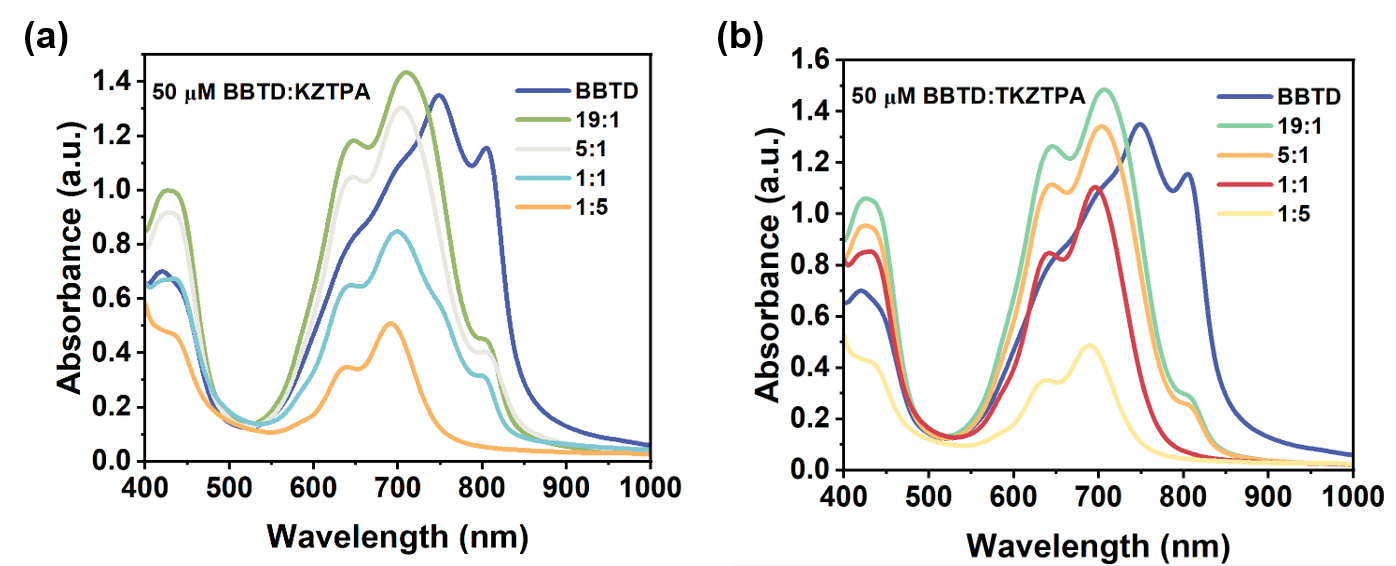


**Figure S21.** UV-Vis absorbance of (a) BBTD@KZTPA and (b) BBTD@TKZTPA with different molar ration of molecules.

**S4.3. Measurement of Fluorescence Emission of BBTD and BBTD@AIEs Bimolecular Systems**

3 mL BBTD (25 μM), BBTD@TPA3OMe (25 μM@25 μM), BBTD@KZTPA (25 μM@25 μM), BBTD@TKZTPA (25 μM @25 μM) (DMF/Water, f_w_=0%, 40%, 60% and 70%) were added in a quartz cuvette, and the emission in the different range were measured.

F_w_=0%, E_x_=400 nm, Range: 420-850 nm.

F_w_=40%, E_x_=808 nm, Range: 850-1200 nm.

F_w_=60%, E_x_=808 nm, Range: 850-1200 nm.

F_w_=70%, E_x_=450 nm, Range: 470-850 nm.

F_w_=70%, E_x_=808 nm, Range: 850-1200 nm.


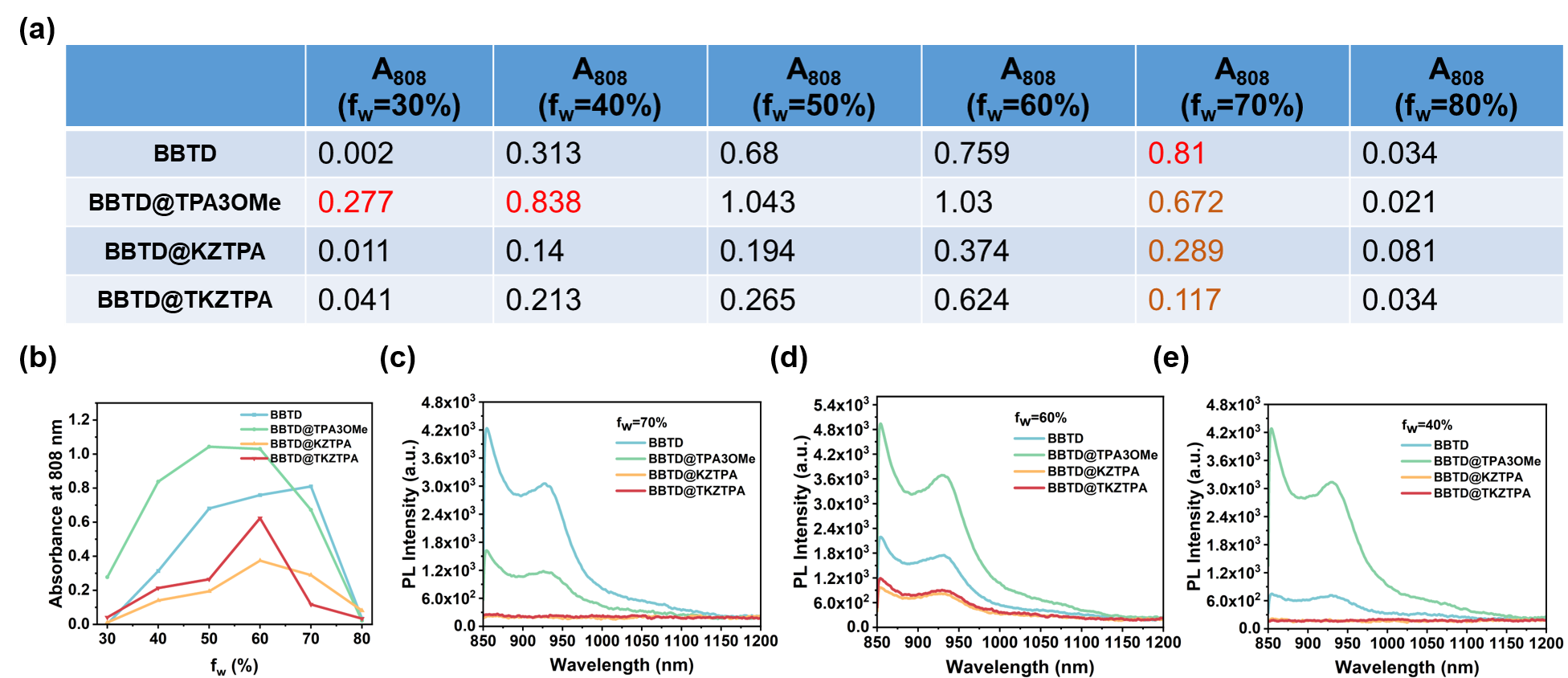


**Figure S22.** Comparison of a) an b) absorbance value at 808 nm (A_808_) and NIR fluorescence emission of BBTD, BBTD@TPA3OMe, BBTD@KZTPA and BBTD@TKZTPA bimolecular systems with different f_w_.

**S4.4. EPR Tests of BBTD and BBTD@AIEs Bimolecular Systems**

Detailed testing conditions are provided in Table S2, and the results are depicted in Figure S23.

**Table S2.** Detailed testing conditions for the detection of ROS generated during photodynamic effect using EPR

| Test item | PDT | | | | |
| --- | --- | --- | --- | --- | --- |
| Excitation source | laser (808 nm, 0.6 W cm^−2^) | | | | |
| ROS | ^1^O_2_ | ^•^O_2_^−^ | | ^•^OH | |
| Trapping agent | TEMP | DMPO | | DMPO | |
| trapping agent dosage | 20 μL | | 10 μL | | 10 μL |
| Solvent | DMF/H_2_O (f_w_=70%) | DMF/MeOH  (f_w_=70%) | | DMF/H_2_O (f_w_=70%) | |
| Amount | 100 μL | | | | |
| Container | 96-well plate | | | | |
| Duration | 3 min | | | | |


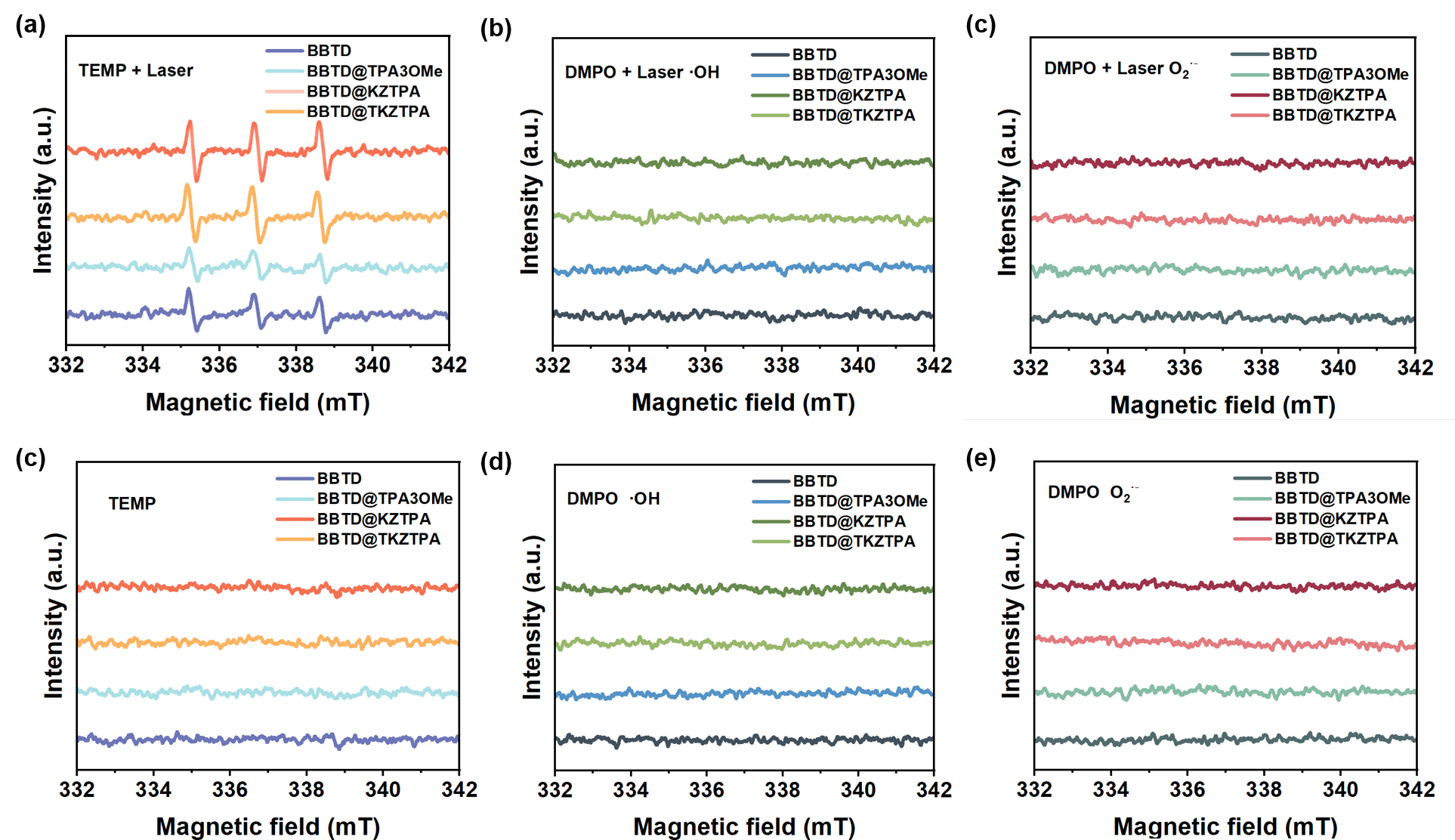


**Figure S23.** EPR spectra of BBTD, BBTD@TPA3OMe, BBTD@KZTPA and BBTD@TKZTPA in the presence or absence of laser irradiation.

**S4.5. Measurement of Photodynamic effect of BBTD and BBTD@AIEs Bimolecular Systems (9,10-diphenylanthracene as probe)**

The 9,10-diphenylanthracene (DPA) was used to specifically evaluate the generation ability of ^1^O_2_. 50 μL DPA (1.0 mg mL^−1^, DMF) was added to 2 mL BBTD (25 μM), BBTD@TPA3OMe (25 μM@25 μM), BBTD@KZTPA (25 μM@25 μM) and BBTD@TKZTPA (25 μM@25 μM) solutions respectively, and 808 nm laser (0.6 W cm^-2^) was used to illuminate. The absorption data of DPA at 375 nm were obtained by UV-Vis absorbance spectra. Blank control as DMF/H_2_O (f_w_=70%) solution were set.


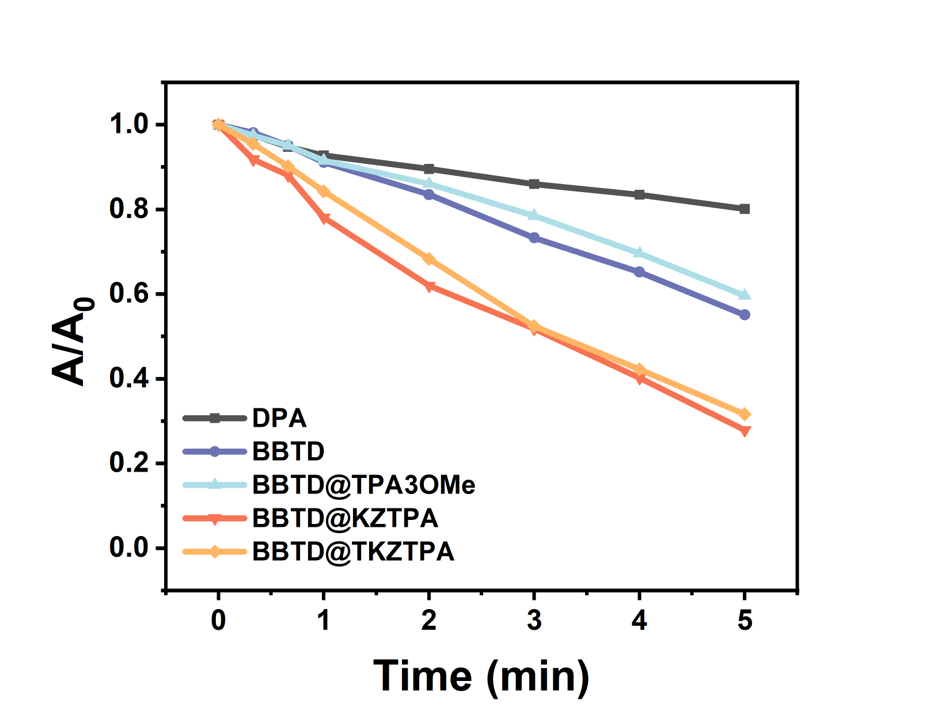


**Figure S24.** Photodynamic effect of BBTD, BBTD@TPA3OMe, BBTD@KZTPA and BBTD@TKZTPA.

**S4.6. Standard Curves of BBTD**

The absorbance at 674 nm (A_674_) of BBTD in DMF solution with different concentrations was measured, and the standard curves were plotted. Since the absorbance values of BBTD and KZTPA at 350-400 nm overlap, the encapsulation rate was calculated by reference to A_674_.


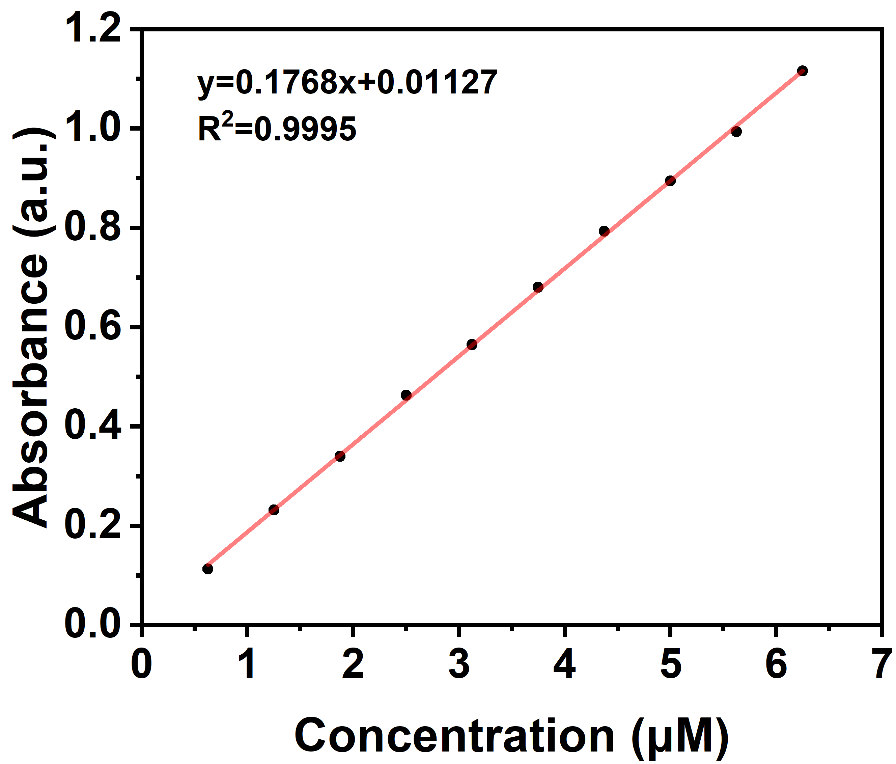


**Figure S25.** Standard cruve of BBTD in DMF.

**S4.7. Measurement of UV-Vis Absorbance of BBTD NPs and BBTD@KZTPA NPs**

2 mL BBTD NPs (20 μM), BBTD@KZTPA NPs A (20 μM) and BBTD@KZTPA NPs B (20 μM) were added in a quartz cuvette, and the absorbance in the range of 400-1000 nm were measured.

**S4.8. Measurement of Fluorescence Emission and Lifetime of BBTD, BBTD@KZTPA NPs A and BBTD@KZTPA NPs B**

3 mL BBTD (20 μM), BBTD@KZTPA NPs A (20 μM) and BBTD@KZTPA NPs B (20 μM) were added in a quartz cuvette, and the emission (E_x_=430 nm, E_m_=760 nm) and life time (E_x_=375 nm, E_m_=820 nm) were measured.


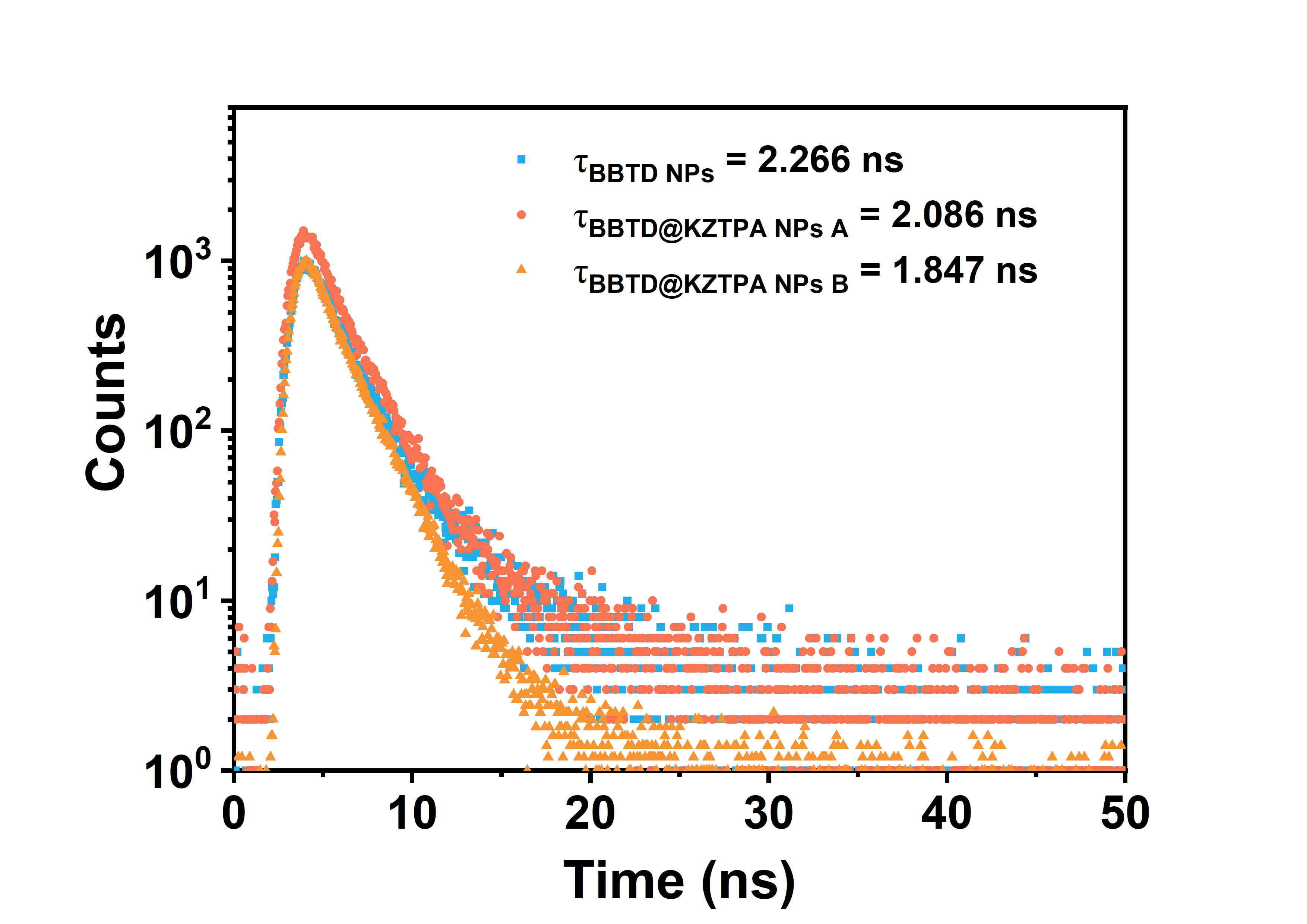


**Figure S26.** The fluorescence lifetime of BBTD NPs, BBTD@KZTPA NPs A and BBTD@KZTPA NPs B.

**S4.9. Measurement of Photothermal conversion efficiency BBTD NPs and BBTD@KZTPA NPs A**

To evaluate the photothermal conversion performance, BBTD NPs and BBTD@KZTPA NPs A were respectively irradiated with 808 nm NIR laser (0.8 W cm^−2^) for 10 min and laser was shut off for 10 min to cool down to room temperature. The temperature was recorded by thermal imaging system every 30 s. According to the reported method,^[1]^ the photothermal conversion efficiency (PCE) were be calculated.

$$\eta=\frac{hs(T_{Max}-T_{Surr}) - Q_{Dis}}{I(1 - {10}^{{-A}_{808}})}$$

Where $\eta$ is the photothermal conversion efficiency, *s* is the surface area of the container, and the value of *hs* is obtained according to the cooling curve. $T_{Max}$ is the maximum temperature of photothermal agents, and $T_{Surr}$ is the surrounding temperature. $Q_{Dis}$ is the energy absorbed by the solvent. *I* is the power of laser power. $A_{808}$ is the absorbance of photothermal agents at 808 nm.

$$hs=\frac{mC_{water}}{\tau_{s}}$$

where $m$ is the mass of the solution, $C$ is the heat capacity of the solution ($C_{Water}$ = 4.2 J/g ∙ ^o^C), and $\tau_{s}$ is a time-dependent constant calculated from the linear time-dependent data recorded in the cooling period.

$$t=-\tau_{s}\ln\left( \theta\right)$$

where *θ* is a dimensionless parameter derived from temperature.

$$\theta=\frac{T-T_{Surr}}{T_{Max}-T_{Surr}}$$

*T_Max_* and *T_Surr_* represent the maximum steady-state temperature and surrounding temperature respectively.


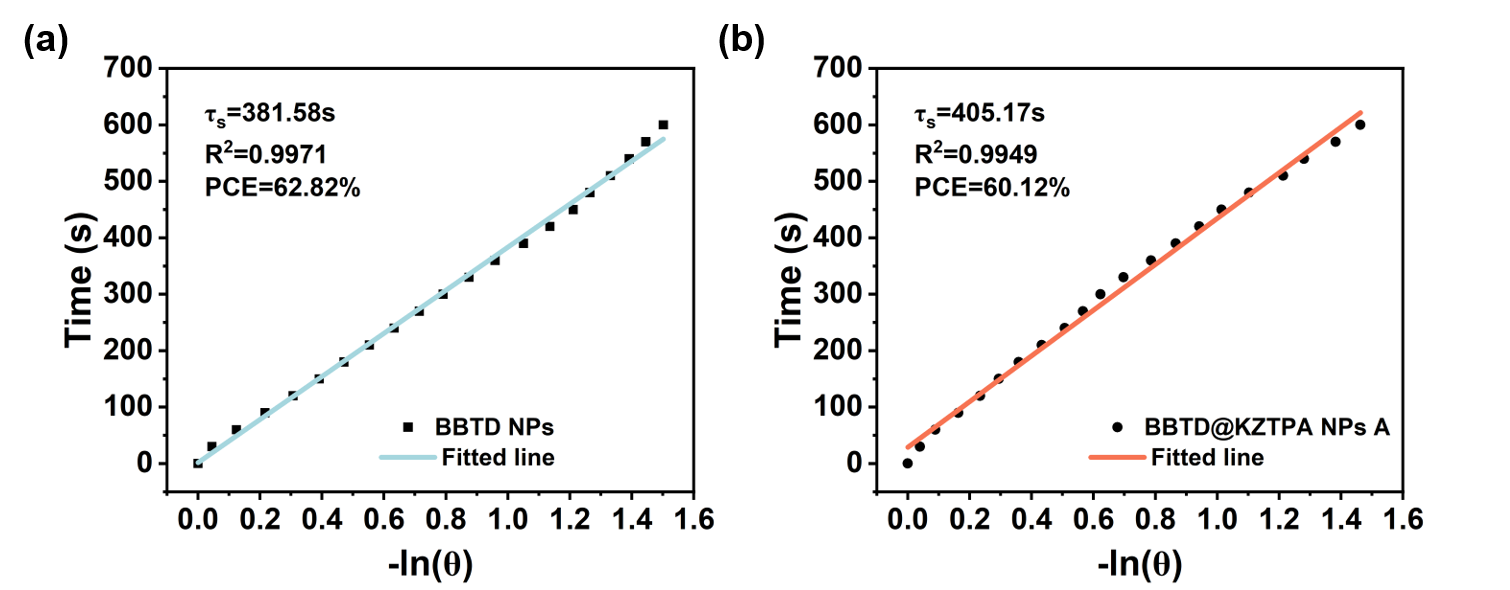


**Figure S27.** PCE of (a) BBTD NPs and (b) BBTD@KZTPA NPs A.

**S4.10. Stability of BBTD@KZTPA NPs A**

The aqueous solution of BBTD@KZTPA NPs A (20 μM) was diluted with ultrapure water into 5 μM. The hydrodynamic sizes, particles distribution index (PDI) zeta potentials and A_808_ of samples were characterized once every two days. Data were presented as means ± SD (*n* = 3).


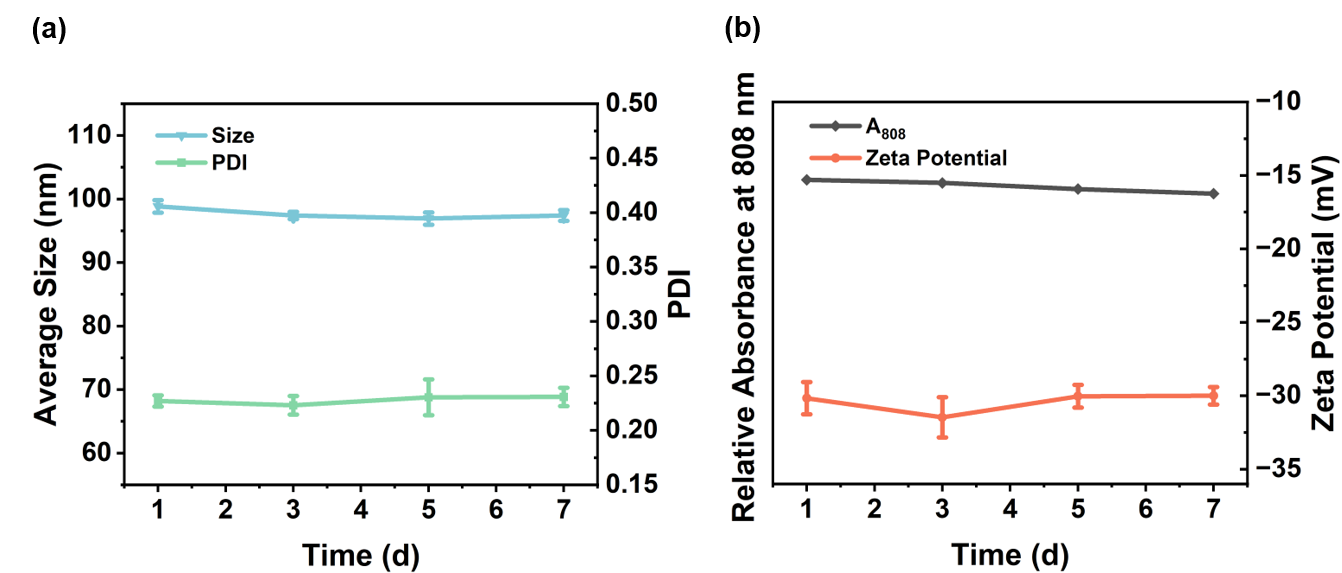


**Figure S28.** The stability of BBTD@KZTPA NPs A. The variations of a) Size and PDI; b) Zeta potentials and relative A_808_ in continuous 7 days.

**S5. Theoretical Calculations**

**S5.1. Conformation Search**

Molecular dynamics simulations of BBTD@AIE systems were performed using xtb at the GFN0-xTB level within the NVT ensemble for 200 ps at 430 K (BBTD@TPA3OMe) or 450 K (BBTD@KZTPA and BBTD@TKZTPA) with a timestep of 1.0 fs, employing the SHAKE algorithm to constrain hydrogen-related bonds. Each trajectory frame was optimized at the GFN0-xTB level, followed by removal of duplicate structures using xtb combined with Molclus. The remaining unique structures were subsequently optimized at the GFN2-xTB level under the GBSA solvent model, retaining only the lowest energy conformation for further analysis. Final geometry optimizations were conducted using Gaussian 09 at the B3LYP/6-31G(d) level of theory incorporating Grimme's D3(BJ)^[2]^ dispersion correction. Intermolecular interactions were quantitatively analyzed using Multiwfn, with molecular visualizations generated in VMD.

**S5.2. Density Functional Theory (DFT) Calculation**

The geometric optimization and time-dependent density functional theory (TD-DFT) calculations for BBTD, BBTD@AIEs and AIEs were conducted using the Gaussian 09 program at the B3LYP/6-31G(d) level of theory with Grimme' s D3(BJ) dispersion correction. Following this, the molecular surface electrostatic potential (ESP) map was constructed using the Multiwfn and VMD software programs. In addition, the geometric structure of S_0_ state was calculated using B3LYP-D3(BJ)/6-31G(d) level and the excitation energy of the S_1_ and T_1_ states were calculated using TD-DFT/B3LYP/6-31G(d) level. The atomic coordinates were summarized in “**S9. Atomic Coordinates**”.

**S5.3. Energy Decomposition Analysis based on sobEDAw for BBTD-dimer and BBTD@AIEs**

Energy decomposition analysis was carried out by utilizing the sobEDAw method at the B3LYP/6-31+G(d) level of theory with Grimme's D3(BJ) dispersion correction to assess the intermolecular interaction.

**S5.4. Molecular dynamics (MD) Simulation**

Classical molecular dynamics (CMD) simulations of BBTD and BBTD@AIEs systems were performed using GROMACS 2019.6 with the AMBER force field^[3]^ describing molecular potential functions for all components (BBTD, KZTPA, TKZTPA, TPA, DMF, and water). Atomic charges were assigned as 1.2×CM5^[5]^, with molecular topologies designated as sobtop designation^[4]^. The simulation system was constructed using packmol^[6]^ in a 100*100*100 nm^3^ boxes containing: (1) For BBTD@AIE systems: 15,600 water molecules, 1,560 DMF molecules, 10 BBTD molecules, and 10 AIE molecules (TPA/KZTPA/TKZTPA); (2) For pure BBTD systems: 15,600 water molecules, 1,560 DMF molecules, and 10 BBTD molecules.

To enhance computational efficiency while maintaining simulation accuracy, we implemented several optimization approaches: (1) A Verlet cutoff scheme with 1.2 nm neighbor list radius for efficient atomic neighbor list updates; (2) The particle-mesh Ewald (PME) method for accurate calculation of long-range electrostatic interactions; (3) A truncated potential approach (1.2 nm cutoff) for van der Waals interactions, with energy-pressure correction (EnerPres) applied to compensate for potential artifacts introduced by the neglect of long-range dispersion effects.

Energy minimization was performed using a conjugate gradient (CG) algorithm with a step size of 0.001 nm. Once the system energy converges to 100 kJ mol^-1^, the algorithm terminates and the output eliminates the relaxation structure of the unreasonable contact. For pre-equilibration, the system was maintained at 300 K using a V-rescale thermostat and 1 atm pressure via Berendsen barostat for 0.2 ns. Production simulations employed the Parrinello-Rahman barostat for NPT ensemble conditions, running for 20 ns with a 0.001 ps timestep. Trajectory data were saved every 1000 steps (1 ps intervals), yielding 20,000 frames for analysis..

Especially, in order to perform the CMD of BBTD and BBTD@TPA3OMe under DMF/Water (f_w_=60%), the settings of the amounts of DMF and Water molecules were changed: 13320 water molecules, 2072 DMF molecules.

As depict in Figure S29a,c, the conformation of J-aggregation in BBTD@TPA3OMe is more than that of BBTD, which explained why the A_808_ of BBTD@TPA3OMe was better than that of BBTD.


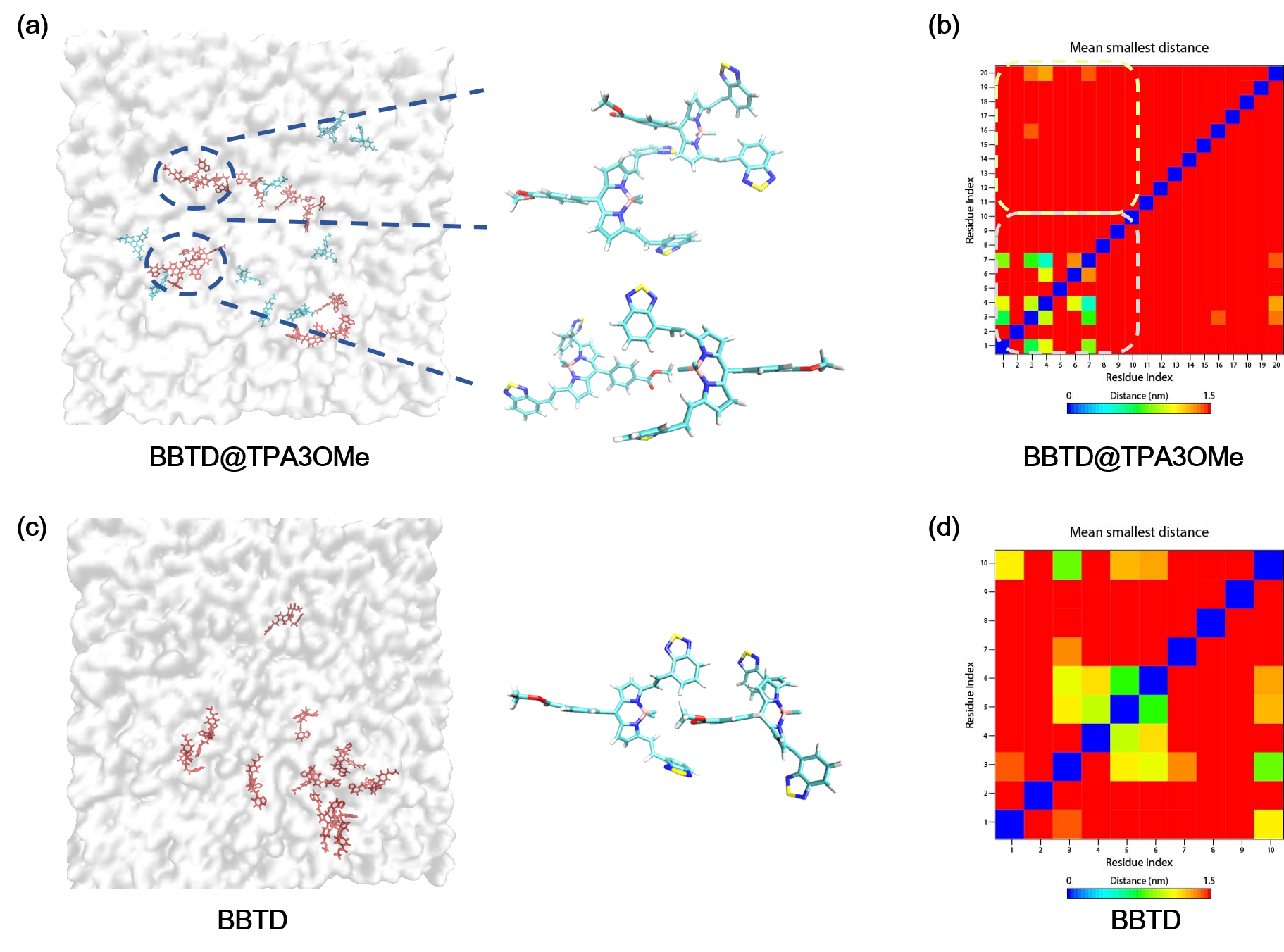


**Figure S29.** Results of MD simulation of a) BBTD@TPA3OMe and c) BBTD under DMF/Water (f_w_=60%) (BBTD: red color; TPA3OMe: cyan color). Color-filled map of the mean smallest intermolecular distances in b) BBTD@TPA3Me and d) BBTD. Residue index 1-10 for BBTD and 11-20 for TPA3OMe).

**S6. In Vitro Study**

CT26.WT cells were purchased from Procell Life Science & Technology Co., Ltd., Wuhan.

**S6.1.** **Cell Uptake Assay**


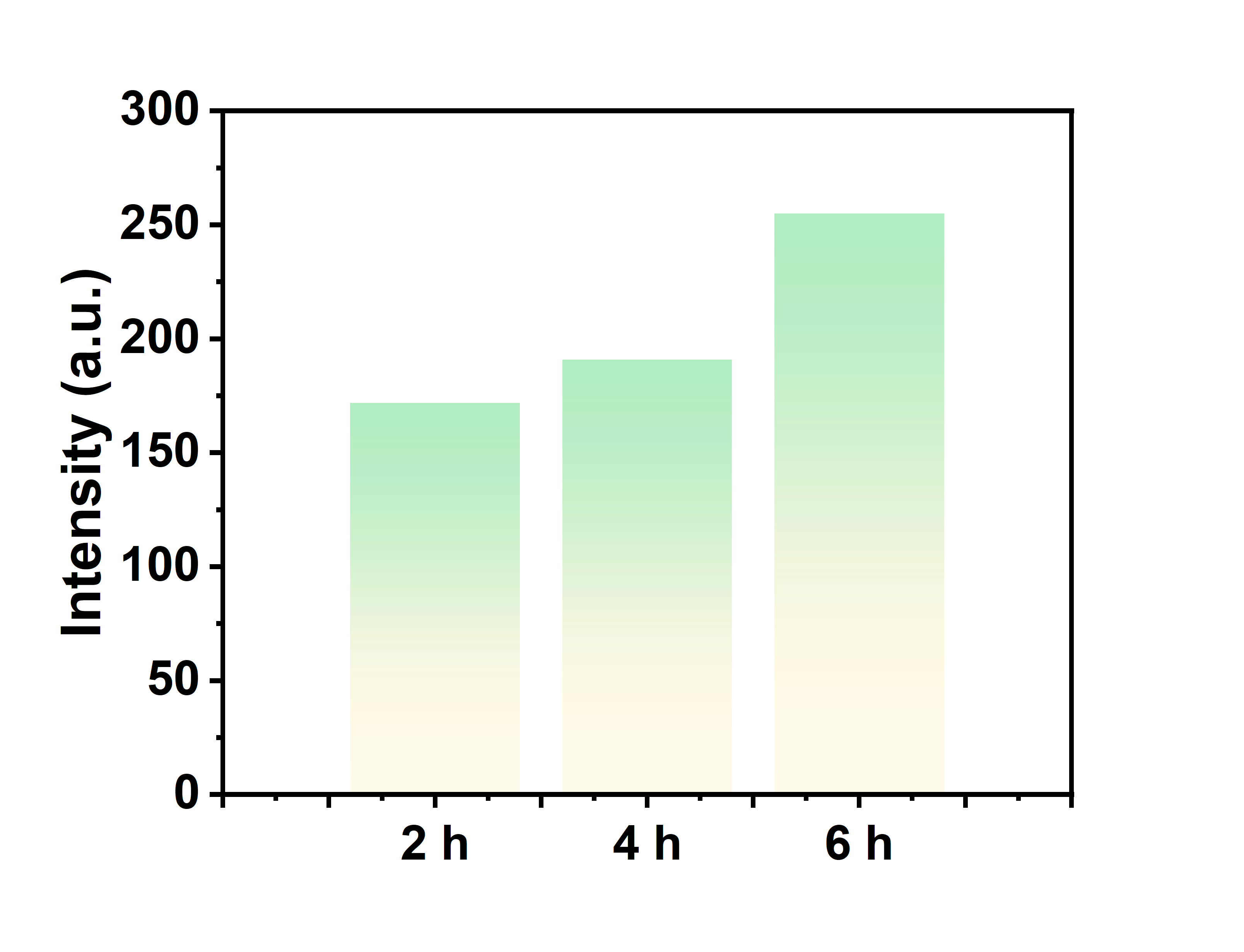


**Figure S30.** Quantitative mean fluorescence intensity after cellular uptake of BBTD@KZTPA NPs A at different time periods.

**S7. In Vivo Study**

**S7.1. Animal purchase and keep**

4-week-old female BALB/c mice were acquired from Beijing Vital River Laboratory Animal Technology Co., Ltd. and acclimatized over a period of 2-3 days until their conditions were deemed stable.  The animal study protocol received approval from the Ethics Committee of Tianjin University (Approval number: TJUE-2024-076). Mice were housed were housed in an SPF condition under 12 h light / 12 h dark and fed ad libitum.

**S7.2. In vivo fluorescence imaging**

CT26 tumor-bearing mice received intravenous injections of BBTD@KZTPA NPs A (100 μM, 0.3 mL) via the tail vein. Whole-body fluorescence imaging was performed at predetermined time intervals (1, 3, 6, 12, 24, 36, and 50 h post-injection) using an IVIS spectrum imaging system. Saline-injected mice served as background controls and were imaged under identical conditions

After 3 h and 50 h administration, the mice were sacrificed to collect the major organs and tumor tissues for ex vivo fluorescence imaging. The image parameters were calculated and analyzed by the instrument's own software.

At 3 h (peak circulation time) and 50 h (prolonged retention phase) post-injection, mice were euthanized for organ harvesting. Major organs (heart, liver, spleen, lungs, kidneys) and tumor tissues were excised for ex vivo fluorescence imaging. Quantitative analysis of fluorescence signals was performed using the instrument’s own software.

In addition, the fluorescence signal intensity at the tumor site at the emission wavelengths of 760 nm and 820 nm were compared.

Comparative analysis of tumor-associated fluorescence signals at 760 nm and 820 nm emission wavelengths revealed significantly higher intensity at 760 nm, consistent with the characteristic emission profile of BBTD@KZTPA NPs A (Figure S31,32). it also suggested that the interaction between BBTD and KZTPA and in vivo Structural integrity of the nanoparticle system during circulation remain stable during circulation.


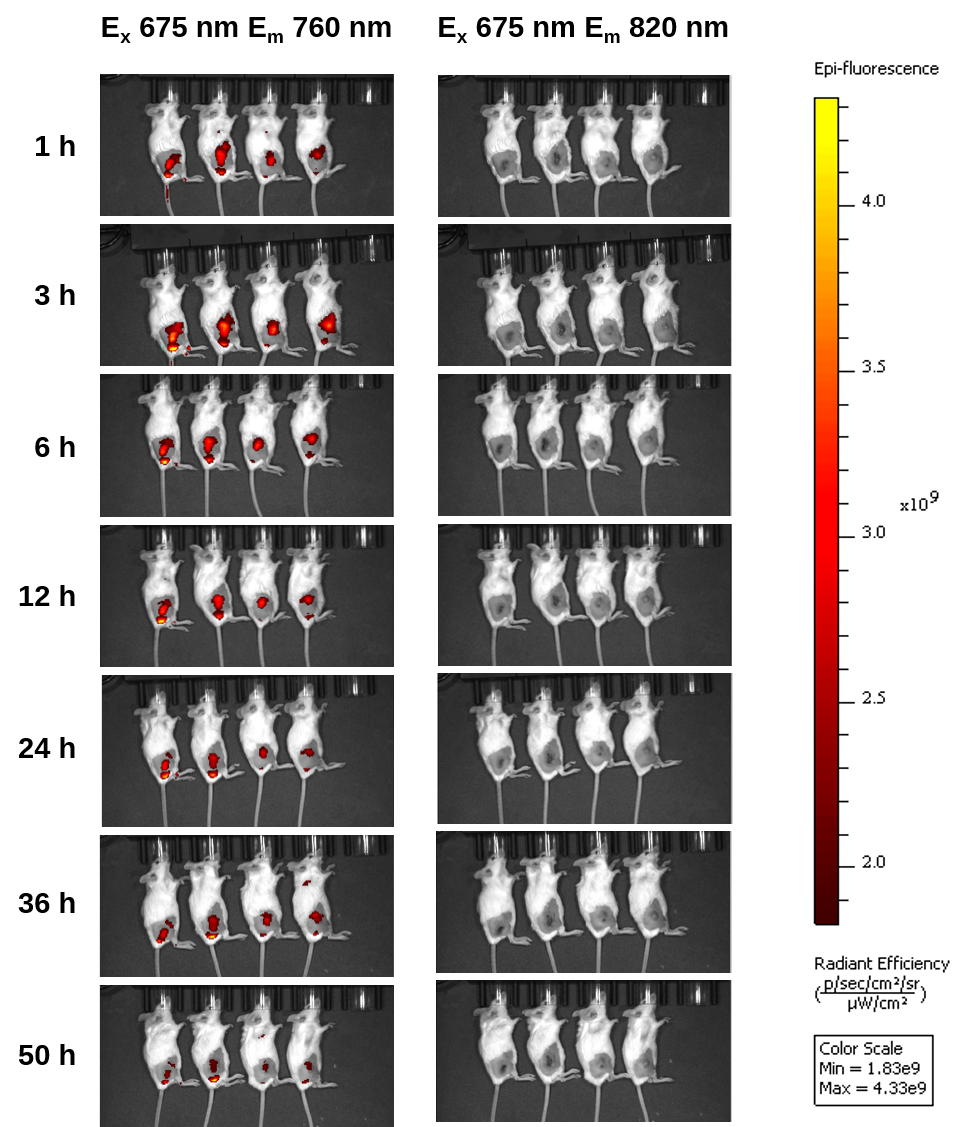


**Figure S31.** In vivo fluorescence images at different time periods and at different emission wavelengths after injection of BBTD@KZTPA NPs A.


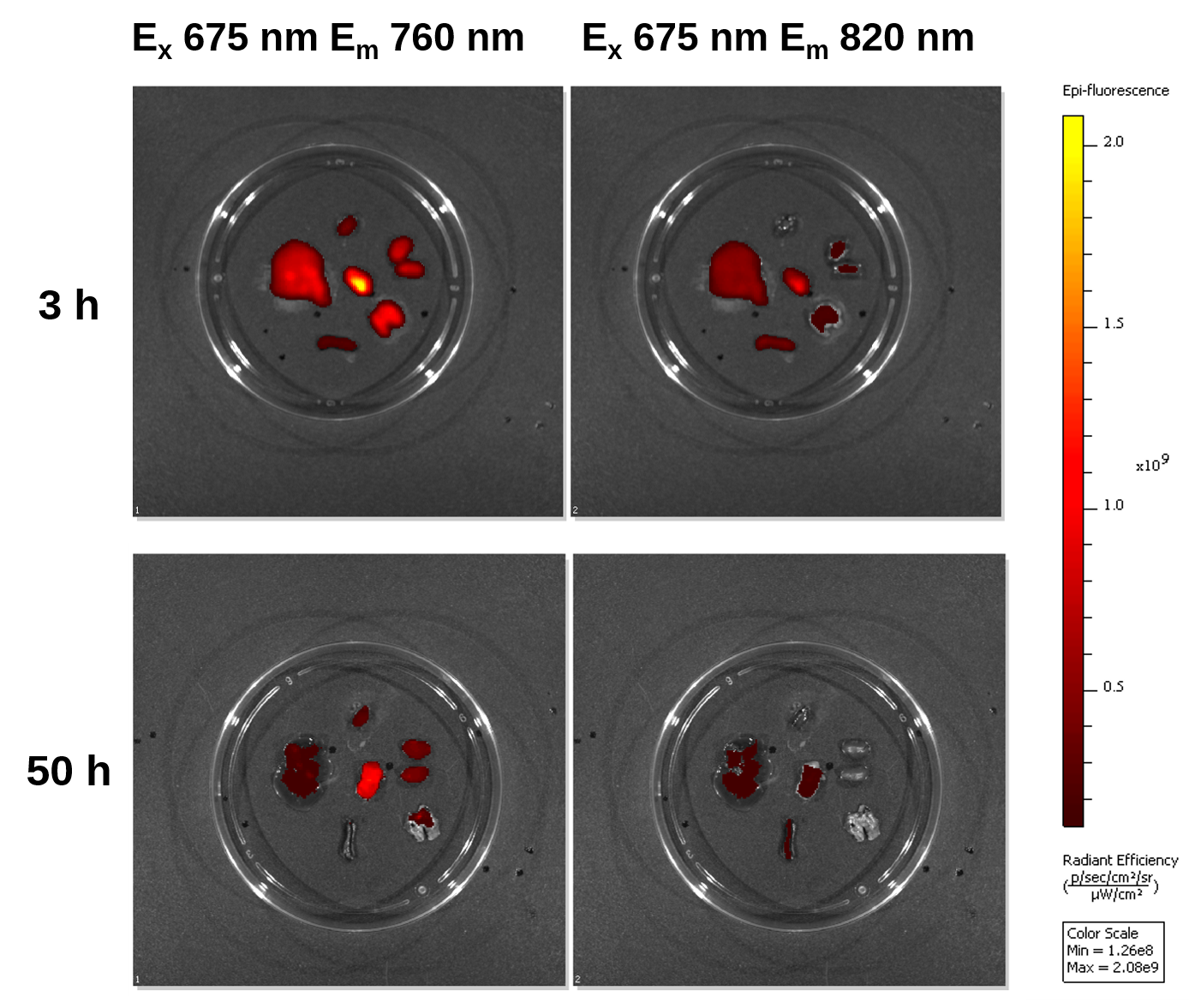


**Figure S32.** Ex vivo images at different time periods and at different emission wavelengths after the tail vein injection of BBTD@KZTPA NPs A.

**S7.3. In vivo photothermal imaging**

Following intravenous administration of either BBTD@KZTPA NPs A (100 μM, 0.3 mL) or saline control, tumor-bearing mice received localized 808 nm laser irradiation (0.8 W cm^-2^) at two treatment time points: 6 h and 24 h post-injection. During laser irradiation, real-time monitoring of the tumor temperature was conducted using an infrared thermal camera.


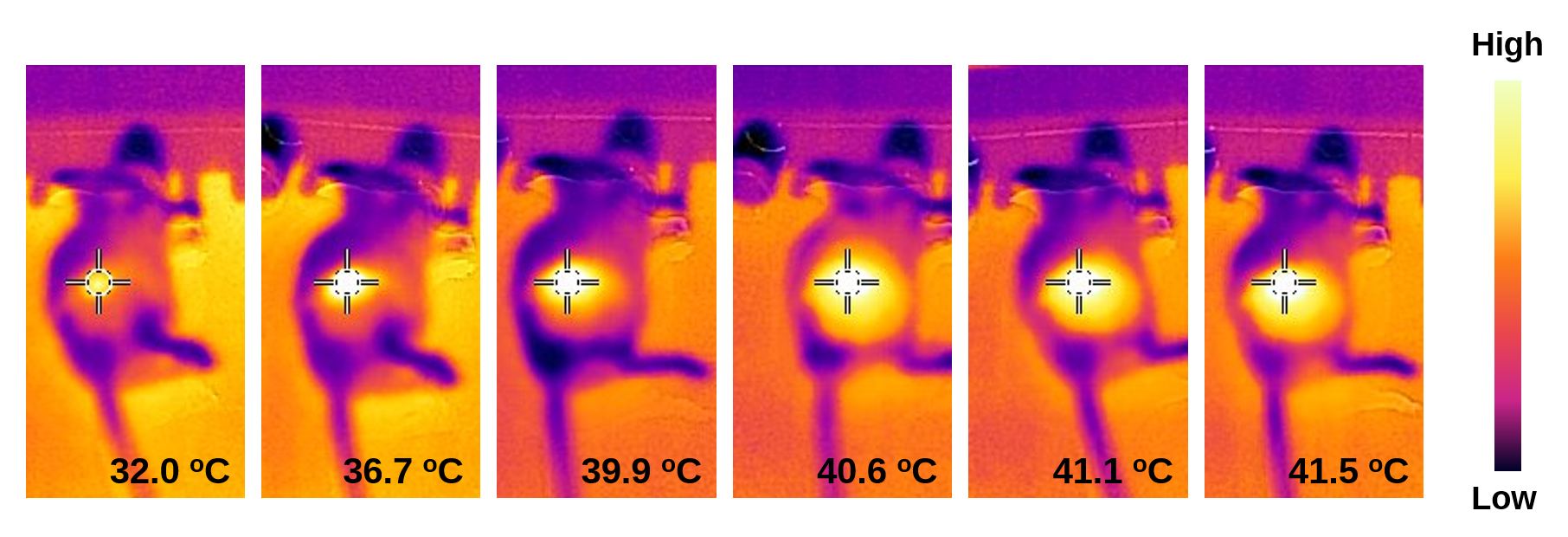


**Figure S33.** Photothermal image performed by laser irradiation after 24 h of the tail vein injection of BBTD@KZTPA NPs A.

Mice were injected BBTD NPs with the same concentration of BBTD@KZTPA NPs A and treated 6 h later. It is very clear that although the high temperature could efficiently inhibit the growth of tumor, it would also cause damage to the skin tissue near the tumor.


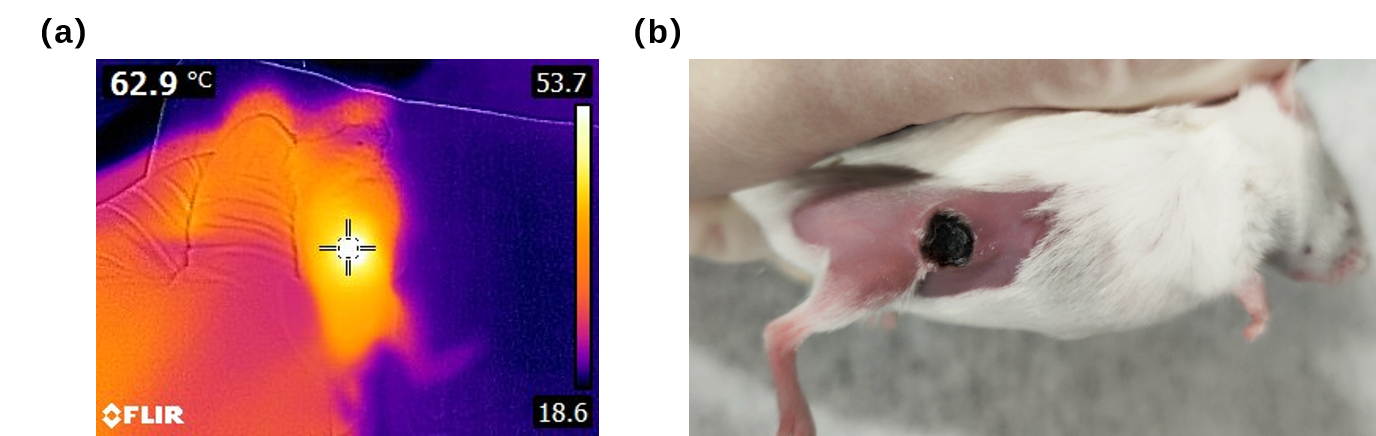


**Figure S34.** a) Photothermal image performed by laser irradiation at 6 h post-injection of BBTD NPs. b) Image of tumor site based on laser irradiation at 6 h post-injection of BBTD NPs. (Laser: 808 nm, 0.8 W cm^-2^; Drug: 100 μM, 0.3 mL)

**S7.4.** **In Vivo evaluation of therapeutical outcomes and biosafety**


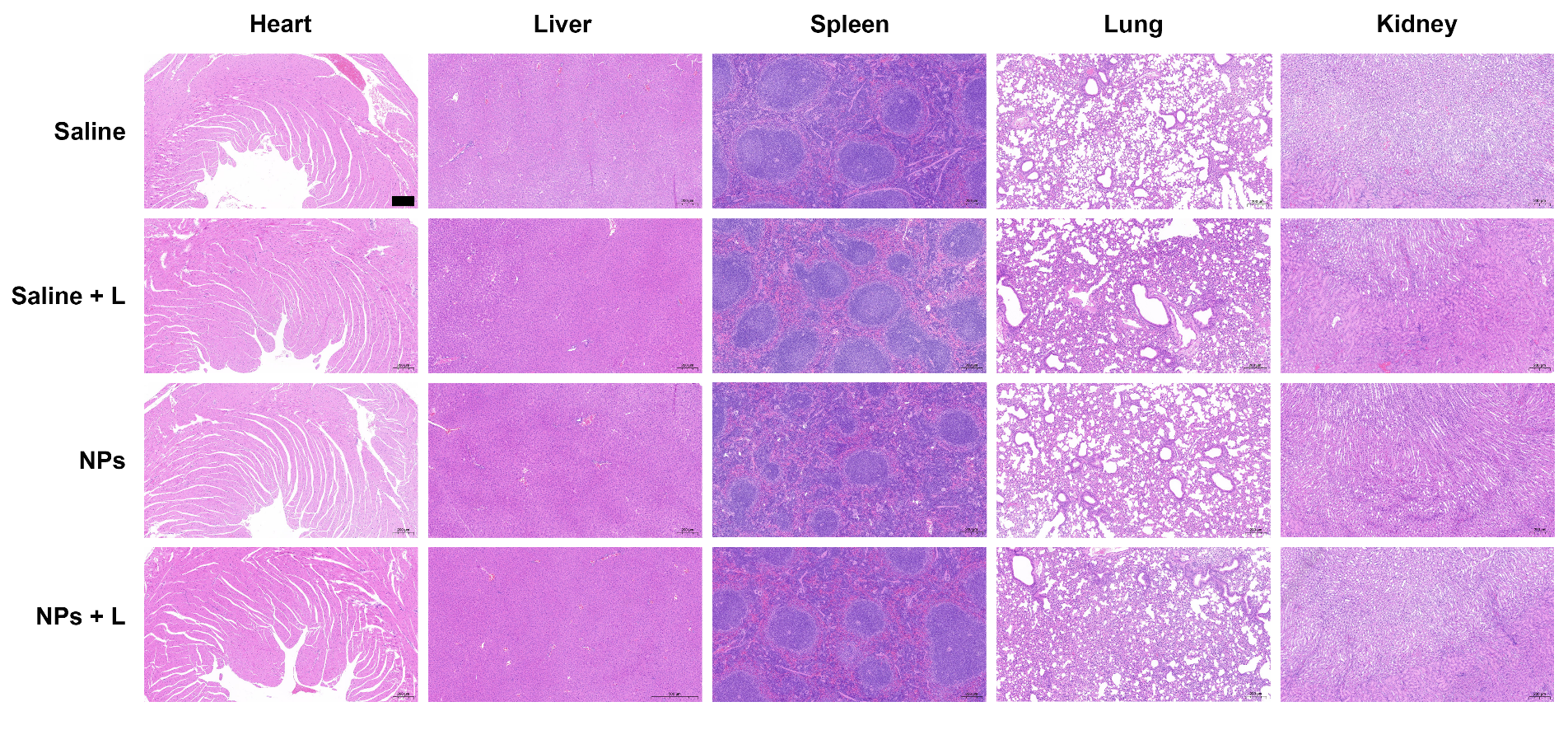


**Figure S35.** H&E staining images of the major organs of mice. (Scale bar: 200 μm)

**S8. Reference**

[1] W. G. Zhang, M. M. Kang, X. Li, H. Yang, Z. J. Zhang, Z. R. Li, Y. B. Zhang, M. Z. Fan, C. R. Liao, C. B. Liu, G. X. Xu, D. Wang, Z. R. Xu, B. Z. Tang, *Adv. Mater.* **2024**, *36*, 2406474.

[2] S. Grimme, S. Ehrlich, L. Goerigk, *J. Comput. Chem.* **2011**, *32*, 1456.

[3] D. A. Case, T. E. Cheatham III, T. Darden, H. Gohlke, R. Luo, K. M. Jr., A, Onufriev, C. Simmerling, B. Wang, R. J. Woods. The Amber Biomolecular Simulation Programs, *J. Comput. Chem.* **2005**, *26*, 1668.

[4] Tian Lu, Sobtop, Version 1.0, http://sobereva.com/soft/Sobtop (accessed October 2024).

[5] A. V. Marenich, S. V. Jerome, C. J. Cramer, D. G. Truhlar. Charge Model 5: An Extension of Hirshfeld Population Analysis for the Accurate Description of Molecular Interactions in Gaseous and Condensed Phases Charge Model 5: An Extension of Hirshfeld Population Analysis for the Accurate Description of Molecular Interactions in Gaseous and Condensed Phases, *J. Chem. Theory Comput.* **2012**, *8*, 527.

[6] L. Martínez, R. Andrade, E.G. Birgin, J.M. Martínez. PACKMOL: A package for building initial configurations for molecular dynamics simulations, *J. Comput. Chem.* **2009**, 30, 2157.

**S9. Atomic Coordinates**

**BBTD-dimer**

S -7.19867362 2.70668757 -0.45042782

N -5.68461935 3.29866887 -0.20056194

N -7.96895407 4.08861004 -0.90442824

C -5.73109688 4.60771206 -0.46051864

C -7.05129470 5.06267150 -0.86909601

C -4.63010357 5.53834313 -0.37229286

C -7.28191949 6.42882614 -1.19234214

C -3.28490946 5.18240820 0.02898939

C -4.92537385 6.85253748 -0.70597887

C -6.21812074 7.29089418 -1.10584022

H -8.27203498 6.75022340 -1.49446183

C -2.85352576 3.95526427 0.41476793

H -2.57817511 6.01076762 0.01061276

H -4.12635642 7.58741297 -0.65595135

H -6.35487891 8.34112980 -1.34601222

C -1.49223077 3.66772212 0.77382939

H -3.55154596 3.12973639 0.44389607

N -1.11424570 2.43931659 1.23116302

C -0.33259120 4.48954865 0.68493635

C 0.25492037 2.44181572 1.44167646

B -2.06127862 1.23251419 1.52246233

C 0.74931401 3.73027324 1.08877924

H -0.31027666 5.50968607 0.32815810

C 0.94108838 1.27268697 1.83147611

F -2.81821846 1.47531229 2.65308846

F -2.88836148 1.00035766 0.41519733

N -1.13180644 0.00180311 1.74548343

H 1.78700808 4.02661455 1.08933166

C 2.40159584 1.31919950 2.05300987

C 0.23702463 0.06870414 1.97314276

C -1.54582500 -1.27872681 1.94560779

C 2.98199019 2.36217837 2.79828916

C 3.23884544 0.33154796 1.50597054

C 0.68096358 -1.23836480 2.33943146

C -0.41452466 -2.06964217 2.30841476

C -2.91360491 -1.67688971 1.77824583

C 4.36008367 2.43689550 2.96281244

H 2.34154581 3.10668051 3.25802111

H 2.81357423 -0.47002022 0.91731096

C 4.61552893 0.40403067 1.67398092

H 1.69706261 -1.51254416 2.57489420

H -0.42632927 -3.13419121 2.48906466

H -3.59704014 -0.94787229 1.36499894

C -3.35152517 -2.92350462 2.08581926

C 5.18856888 1.46291290 2.38750786

H 4.79952654 3.24617660 3.53418029

H 5.26406908 -0.34097520 1.23002737

H -2.64681807 -3.63756438 2.50743904

C -4.67783916 -3.45838546 1.87405679

C 6.67162174 1.51648298 2.47873127

C -4.93999623 -4.78337875 2.18988053

C -5.77064610 -2.72308094 1.28549385

O 7.10550964 2.58091804 3.19253528

O 7.42317821 0.71028548 1.96596713

H -4.13899253 -5.37849012 2.61839427

C -6.18963190 -5.41520380 1.94514715

N -5.74847157 -1.44636733 0.89872206

C -7.04107258 -3.38044179 1.02060953

C 8.53102182 2.69222439 3.31127636

H -6.30017998 -6.46056306 2.21705457

C -7.23814833 -4.74678688 1.36480224

S -7.22085514 -1.11990448 0.24145559

N -7.94389208 -2.58840774 0.43072424

H 8.70552250 3.58724165 3.90866276

H 8.99102594 2.79055893 2.32444852

H 8.94387027 1.81017605 3.80754472

H -8.19217303 -5.21983398 1.16181206

S 2.42455243 -6.68523244 -0.60196258

N 3.36930177 -5.35903794 -0.83184242

N 3.59198137 -7.83431970 -0.43602623

C 4.63850162 -5.77199138 -0.79072438

C 4.76589027 -7.20295561 -0.56115396

C 5.81418601 -4.94676761 -0.94629987

C 6.04776330 -7.81549252 -0.48541377

C 5.78775604 -3.51674899 -1.17508431

C 7.03006278 -5.60960446 -0.85918544

C 7.14786102 -7.00932179 -0.63500982

H 6.12434656 -8.88314517 -0.31447776

C 4.67990277 -2.74239508 -1.28955967

H 6.77050575 -3.05417860 -1.25295974

H 7.94143813 -5.02794848 -0.96856561

H 8.14304808 -7.44093243 -0.58270651

C 4.71062892 -1.31988185 -1.48464122

H 3.70411494 -3.20174377 -1.20890491

N 3.55682422 -0.59836443 -1.59573923

C 5.81070831 -0.41643803 -1.53584191

C 3.87799676 0.74468223 -1.68729321

B 2.11230867 -1.19036481 -1.64437697

C 5.29500459 0.86045388 -1.66318226

H 6.85335437 -0.68984462 -1.45928037

C 2.87653748 1.73085406 -1.74775555

F 1.88119553 -1.92247242 -0.47010758

F 1.95045232 -1.98914959 -2.75801469

N 1.14375034 0.02738812 -1.68869487

H 5.84643873 1.78718810 -1.72565681

C 3.23555211 3.16043361 -1.63929644

C 1.52844676 1.35939869 -1.78251578

C -0.21587155 -0.03244335 -1.64831073

C 4.03646859 3.58334474 -0.56412525

C 2.73645255 4.11928602 -2.53484944

C 0.34213278 2.15396950 -1.81143301

C -0.73207123 1.29692245 -1.74510543

C -0.92372186 -1.27012279 -1.48682548

C 4.31210329 4.93265275 -0.37493558

H 4.40626330 2.85032354 0.14234479

H 2.13737040 3.79736174 -3.38007576

C 3.01465149 5.46773443 -2.34826927

H 0.31301590 3.23169449 -1.82393080

H -1.77421247 1.56351758 -1.67093834

H -0.34340063 -2.14606134 -1.22925529

C -2.26860275 -1.35966375 -1.64253747

C 3.79688486 5.88485699 -1.26394410

H 4.91486033 5.25226624 0.46715326

H 2.63496826 6.21777524 -3.03347530

H -2.82648093 -0.46471800 -1.90416348

C -3.07754803 -2.55284522 -1.52682672

C 4.04299946 7.34321726 -1.10304881

C -4.40789549 -2.51838531 -1.91606417

C -2.60500679 -3.81532050 -1.01304013

O 4.82028124 7.61825855 -0.03142916

O 3.59940209 8.20136225 -1.84064935

H -4.81032494 -1.57968535 -2.28488429

C -5.27442500 -3.64445236 -1.85213046

N -1.38514823 -4.06718999 -0.53443554

C -3.50194090 -4.95882528 -0.93130431

C 5.09198641 9.01149964 0.18052763

H -6.30382569 -3.52295233 -2.17500604

C -4.85035637 -4.85790028 -1.37360424

S -1.38864140 -5.63045320 -0.01705434

N -2.94014844 -6.04500501 -0.38863521

H 5.72097579 9.05419742 1.06980925

H 5.61269517 9.43515961 -0.68205694

H 4.16161073 9.56312799 0.33843170

H -5.50676596 -5.71609264 -1.29738597

**BBTD@TPA3OMe**

S -4.63100000 1.93400000 -0.16100000

N -3.17800000 2.38500000 -0.78400000

N -5.41100000 3.37900000 -0.19600000

C -3.24700000 3.69800000 -1.02400000

C -4.54100000 4.27000000 -0.69100000

C -2.18900000 4.54300000 -1.52200000

C -4.80100000 5.65400000 -0.88700000

C -0.83000000 4.10700000 -1.75800000

C -2.50800000 5.88400000 -1.68300000

C -3.78400000 6.43000000 -1.38300000

H -5.77800000 6.05600000 -0.64300000

C -0.33900000 2.85100000 -1.62100000

H -0.14100000 4.91500000 -2.00000000

H -1.73300000 6.55600000 -2.04200000

H -3.94400000 7.49200000 -1.54500000

C 1.06900000 2.56300000 -1.61700000

H -1.00800000 2.02900000 -1.40800000

N 1.55100000 1.29100000 -1.55900000

C 2.17000000 3.46600000 -1.51200000

C 2.92900000 1.34200000 -1.40000000

B 0.69900000 -0.01400000 -1.65100000

C 3.31600000 2.71200000 -1.36200000

H 2.09400000 4.54400000 -1.49700000

C 3.69200000 0.17600000 -1.24000000

F -0.26200000 -0.02300000 -0.64000000

F 0.09500000 -0.11200000 -2.89300000

N 1.69700000 -1.19500000 -1.44900000

H 4.32400000 3.07000000 -1.21400000

C 5.14700000 0.26600000 -0.99100000

C 3.06700000 -1.07900000 -1.27900000

C 1.36500000 -2.51200000 -1.42600000

C 5.70200000 -0.33700000 0.15100000

C 5.98700000 0.96000000 -1.87800000

C 3.61200000 -2.39100000 -1.19200000

C 2.55600000 -3.27900000 -1.27400000

C -0.00600000 -2.93600000 -1.50900000

C 7.06800000 -0.24600000 0.39700000

H 5.06100000 -0.84600000 0.86300000

H 5.56200000 1.41100000 -2.76900000

C 7.35200000 1.04300000 -1.63300000

H 4.66400000 -2.61900000 -1.10000000

H 2.60200000 -4.35900000 -1.26400000

H -0.71000000 -2.24600000 -1.95600000

C -0.44600000 -4.10300000 -0.98100000

C 7.90100000 0.44100000 -0.49500000

H 7.49000000 -0.70000000 1.28600000

H 8.01300000 1.56700000 -2.31500000

H 0.26700000 -4.75300000 -0.47600000

C -1.82300000 -4.54800000 -0.92000000

C 9.36900000 0.56700000 -0.28100000

C -2.17300000 -5.61900000 -0.11300000

C -2.90500000 -3.91800000 -1.63400000

O 9.78100000 -0.05700000 0.84500000

O 10.12400000 1.16200000 -1.02600000

H -1.38700000 -6.12200000 0.44500000

C -3.51000000 -6.07600000 0.04500000

N -2.80300000 -2.88700000 -2.47500000

C -4.26800000 -4.39800000 -1.47700000

C 11.18800000 0.03500000 1.10900000

H -3.69500000 -6.90900000 0.71700000

C -4.56000000 -5.48900000 -0.61300000

S -4.32000000 -2.55200000 -3.01300000

N -5.16300000 -3.71300000 -2.20200000

H 11.49000000 1.08000000 1.21700000

H 11.76100000 -0.41700000 0.29500000

H 11.34600000 -0.51000000 2.04100000

H -5.58400000 -5.82000000 -0.48900000

C -2.14300000 -1.02600000 1.97800000

C -2.50300000 -2.01000000 2.90900000

C -3.63600000 -2.78900000 2.70800000

C -4.45000000 -2.57400000 1.58900000

C -4.06900000 -1.62700000 0.63100000

C -2.90700000 -0.87700000 0.81700000

H -1.90000000 -2.13900000 3.80200000

H -3.94100000 -3.53600000 3.43500000

H -4.66800000 -1.47200000 -0.26000000

H -2.57800000 -0.16800000 0.06700000

N -1.05600000 -0.14100000 2.24100000

C -1.33900000 1.24800000 2.21400000

C -2.53400000 1.72000000 2.77000000

C -0.47500000 2.17700000 1.61200000

C -2.88600000 3.06800000 2.69800000

H -3.21200000 1.01700000 3.24200000

C -0.80000000 3.52500000 1.57700000

H 0.44200000 1.83300000 1.15300000

C -2.01800000 3.98200000 2.09400000

H -3.83200000 3.38700000 3.11900000

H -0.13700000 4.23800000 1.10200000

C 0.25200000 -0.64600000 2.35300000

C 0.57100000 -1.91100000 1.84600000

C 1.27300000 0.08500000 2.99000000

C 1.86000000 -2.43100000 1.95300000

H -0.19100000 -2.48900000 1.34000000

C 2.56600000 -0.41400000 3.06500000

H 1.05200000 1.05500000 3.42100000

C 2.87300000 -1.67500000 2.54400000

H 2.06500000 -3.40700000 1.53500000

H 3.35400000 0.15900000 3.54400000

O 4.19000000 -2.07100000 2.64500000

O -2.27200000 5.31900000 1.94200000

O -5.58900000 -3.32900000 1.51900000

C 4.49300000 -3.41100000 2.29900000

H 5.55600000 -3.54600000 2.50900000

H 3.90900000 -4.12000000 2.90200000

H 4.30400000 -3.60600000 1.23600000

C -3.49300000 5.81400000 2.46000000

H -4.35600000 5.31800000 2.00000000

H -3.54600000 5.69300000 3.55100000

H -3.51700000 6.87700000 2.21100000

C -6.65700000 -2.83500000 0.71700000

H -7.52900000 -3.43700000 0.98100000

H -6.85900000 -1.78000000 0.94300000

H -6.45300000 -2.94900000 -0.35200000

**BBTD@KZTPA**

S -0.85493733 -5.68929320 1.19618435

N -1.62236889 -4.24683491 1.39760402

N -2.11787717 -6.71296609 1.47547901

C -2.89409151 -4.51795588 1.69596419

C -3.18192174 -5.94398122 1.73317222

C -3.93987319 -3.55114243 1.93349806

C -4.49867700 -6.41164534 1.99708889

C -3.75377905 -2.11666618 1.90834719

C -5.19733079 -4.07668328 2.18851156

C -5.47227255 -5.47181185 2.21956601

H -4.70046556 -7.47633534 2.00799290

C -2.58570686 -1.45062241 1.71784073

H -4.66436504 -1.54758218 2.08012766

H -6.01991180 -3.38675848 2.35277923

H -6.49003374 -5.79198057 2.42303646

C -2.48196086 -0.02113592 1.80297911

H -1.67418750 -2.00415237 1.54034448

N -1.30441456 0.64610214 1.61911012

C -3.48528538 0.92940722 2.14602450

C -1.52185281 2.00134458 1.82012543

B 0.07324160 0.03480466 1.22795363

C -2.89433441 2.17464779 2.16366803

H -4.51457092 0.69534697 2.37259143

C -0.46854181 2.93557879 1.76872049

F 0.31352786 -1.12523298 1.94815632

F 0.10642892 -0.22702331 -0.15140673

N 1.13835815 1.12172457 1.57011017

H -3.36079340 3.11227302 2.42364206

C -0.76325598 4.37098796 1.95651828

C 0.85294177 2.48152151 1.60991766

C 2.49365882 0.95804893 1.58779147

C 0.03539024 5.18785042 2.77919935

C -1.87705672 4.94859840 1.32356626

C 2.09167831 3.18598345 1.57411964

C 3.10169820 2.24590736 1.58186833

C 3.11439532 -0.33722632 1.61743320

C -0.24701718 6.54118094 2.92192862

H 0.86398661 4.74949836 3.32324741

H -2.48812980 4.34206853 0.67137812

C -2.16807487 6.29675340 1.47610628

H 2.20063078 4.25803178 1.52086314

H 4.16490213 2.43206033 1.53710111

H 2.48092570 -1.19300239 1.42967906

C 4.42982142 -0.52353969 1.88884548

C -1.34579693 7.10764874 2.26537135

H 0.37631205 7.17910814 3.53920866

H -3.00545176 6.73567753 0.94755938

H 5.04283883 0.34006419 2.14187973

C 5.12945848 -1.79088933 1.93404787

C -1.54938239 8.57658218 2.36909172

C 6.40326963 -1.86062410 2.47829680

C 4.58966271 -3.03595066 1.44463131

O -2.69813055 8.97788065 1.78545523

O -0.76461067 9.34158319 2.89725381

H 6.86444349 -0.94308316 2.83086459

C 7.13335209 -3.07361146 2.61302065

N 3.42830050 -3.19580320 0.80310981

C 5.33682118 -4.27415877 1.59333155

C -2.90690817 10.39879840 1.75863626

H 8.12494911 -3.03111216 3.05337572

C 6.62167726 -4.27820716 2.20226463

S 3.30503630 -4.79295439 0.42857792

N 4.71455884 -5.34054161 1.07524116

H -2.85207832 10.81360946 2.76775706

H -3.90429522 10.53517329 1.33901625

H -2.15672500 10.87358985 1.12260191

H 7.17563377 -5.20476171 2.28012633

C 2.55566686 2.66443063 -1.86196514

C 2.80120875 4.05876161 -1.84596222

C 1.77971863 5.00594068 -1.83407373

C 0.46054336 4.54660273 -1.81134837

C 0.19128069 3.15845219 -1.80855036

C 1.22483913 2.23165225 -1.84884476

H 3.82512343 4.41694446 -1.82647507

H 2.01161793 6.06503210 -1.84373302

H 0.97978523 1.17912640 -1.86428024

C -1.80413485 4.30528262 -1.81116310

C -3.18915643 4.48068548 -1.73434175

C -3.98955166 3.34161255 -1.63236426

C -3.44711724 2.03725754 -1.61593499

C -2.05914949 1.88099674 -1.68821449

C -1.24364618 3.00389092 -1.78740003

H -3.63442709 5.46955687 -1.73170343

H -5.06855268 3.45963886 -1.58665071

H -1.61613392 0.89506432 -1.59707094

N -0.75757145 5.24316912 -1.82373224

C -0.89914866 6.64682243 -1.71985326

C -1.80162095 7.33406165 -2.53176483

C -0.14818779 7.36403472 -0.77627875

C -1.98053952 8.71251055 -2.39303723

H -2.36530824 6.79132909 -3.28303355

C -0.29823968 8.73645042 -0.65334011

H 0.52569874 6.83255940 -0.11467278

C -1.22574161 9.41883645 -1.45213927

H -2.69637987 9.21715860 -3.03066432

H 0.26198276 9.29410679 0.08978955

C -4.29260216 0.82707759 -1.53668230

C -5.33247605 0.68726996 -0.60759527

C -4.02910372 -0.26332100 -2.38098979

C -6.03879650 -0.50553017 -0.48442943

H -5.55314390 1.50826319 0.06788948

C -4.75588059 -1.44155313 -2.29836679

H -3.25338743 -0.17086414 -3.13538853

C -5.75501860 -1.59702043 -1.32412740

H -6.81097162 -0.59987537 0.27141946

H -4.54316232 -2.25614004 -2.98088355

C 3.65484252 1.67402915 -1.86692472

C 4.97458877 2.01120599 -2.21862741

C 3.41668370 0.33803497 -1.49834076

C 6.00207075 1.07645219 -2.17600402

H 5.20558488 3.01606329 -2.55790882

C 4.43632249 -0.59973593 -1.44446050

H 2.42902778 0.02657374 -1.18074974

C 5.74974794 -0.24217011 -1.76889235

H 7.01203657 1.36374665 -2.45016717

H 4.21470922 -1.60469950 -1.10746372

O -1.32396124 10.76448753 -1.23003061

C -2.25092531 11.49848802 -2.01323463

H -2.00609415 11.44166894 -3.08159791

H -3.27793302 11.14132790 -1.85990954

H -2.17287621 12.53403834 -1.67866947

N 6.81539390 -1.17063756 -1.65181956

N -6.44785895 -2.81976003 -1.19296769

C 8.00382521 -0.75718565 -0.99044342

C 7.95260350 0.08796808 0.12061704

C 9.25829387 -1.19688326 -1.44243580

C 9.11836137 0.49605736 0.77117675

H 6.98974736 0.44060565 0.47172151

C 10.41956046 -0.81608791 -0.78743279

H 9.31044296 -1.85154771 -2.30598838

C 10.36106859 0.03688835 0.32402003

H 9.04121093 1.15956373 1.62464881

H 11.39174615 -1.15611482 -1.12911774

C 6.58574618 -2.54574282 -1.89075009

C 7.18048918 -3.51758058 -1.08310146

C 5.74136123 -2.94751709 -2.94311977

C 6.93265258 -4.87552333 -1.30785504

H 7.82103613 -3.23930557 -0.25729306

C 5.48678857 -4.29769903 -3.13812895

H 5.28590463 -2.20075486 -3.58265733

C 6.06902162 -5.28002164 -2.33024165

H 4.82644031 -4.60148869 -3.94574086

H 5.85282410 -6.32620259 -2.50595851

C -5.80523297 -4.05757184 -1.47267375

C -4.44508841 -4.24785593 -1.20868471

C -6.53453616 -5.13085629 -2.01023803

C -3.82494727 -5.47033682 -1.46141418

H -3.85691286 -3.43402264 -0.80453254

C -5.92971820 -6.35953821 -2.23452460

H -7.58710663 -5.00073083 -2.23702230

C -4.56892245 -6.54523290 -1.95800108

H -2.76795698 -5.57301138 -1.24846627

H -6.49572779 -7.19298147 -2.63805134

C -7.78797129 -2.82356346 -0.72204731

C -8.72638632 -1.91157794 -1.23324004

C -8.20882164 -3.73789536 0.24753326

C -10.03217347 -1.90172261 -0.76689327

H -8.41731614 -1.20353814 -1.99490512

C -9.52754389 -3.75053382 0.70501162

H -7.50061605 -4.46357841 0.62900755

C -10.44688415 -2.82262460 0.20567752

H -10.75947405 -1.19685271 -1.15659192

H -9.81879700 -4.48006048 1.45169579

O -11.75543255 -2.73657926 0.58939057

O -4.06816153 -7.79033973 -2.20934205

O 11.56192758 0.36092233 0.89098191

O 7.56985529 -5.73052606 -0.45352953

C -12.21677141 -3.65134799 1.56746862

H -12.12374629 -4.69042723 1.22394426

H -11.67267040 -3.53762040 2.51483971

H -13.27066154 -3.41677158 1.72635509

C -2.70779390 -8.03089781 -1.87994363

H -2.51973312 -7.87691957 -0.81041902

H -2.51859002 -9.07432383 -2.13862524

H -2.03434864 -7.38779954 -2.46284902

C 7.19769004 -7.09995476 -0.49320652

H 7.77555499 -7.58833019 0.29388615

H 6.12806806 -7.21827508 -0.28815948

H 7.44913520 -7.55781127 -1.45880831

C 11.54831032 1.22018003 2.01633318

H 10.98818423 0.78099262 2.85314504

H 12.59146105 1.35005968 2.30959990

H 11.11517750 2.19950542 1.77178686

**BBTD@TKZTPA**

S 4.75692143 0.38964579 -1.23396685

N 3.22513667 -0.14855979 -1.50670868

N 5.57285797 -1.03728129 -1.34565259

C 3.29839888 -1.47144962 -1.67293841

C 4.65703132 -1.98352176 -1.58349894

C 2.19376812 -2.37717757 -1.88954678

C 4.92142534 -3.37538185 -1.72014530

C 0.80188532 -1.97962863 -1.92736504

C 2.52583510 -3.71581647 -2.02923970

C 3.85558167 -4.20979162 -1.93993778

H 5.93966107 -3.73837904 -1.63759945

C 0.32954224 -0.71611745 -1.79488653

H 0.10135413 -2.80187590 -2.06633987

H 1.72613297 -4.43371573 -2.17959193

H 4.01357338 -5.28044740 -2.02662250

C -1.06253131 -0.36818602 -1.80113556

H 1.02910432 0.09617266 -1.66195435

N -1.47007423 0.93036217 -1.80832217

C -2.22411617 -1.20013560 -1.77409732

C -2.85499049 0.96962864 -1.78622128

B -0.56394457 2.17343920 -2.01575566

C -3.32995343 -0.37444156 -1.76421237

H -2.21560229 -2.28020989 -1.73131355

C -3.53842952 2.18707447 -1.69213992

F 0.51529456 2.12290015 -1.13155673

F -0.10205302 2.19961287 -3.32924992

N -1.43157826 3.42904656 -1.71936674

H -4.36876338 -0.66218961 -1.69978442

C -5.01840746 2.21606413 -1.71214894

C -2.81838557 3.39298620 -1.60549805

C -1.02479270 4.73151999 -1.61851401

C -5.74830631 2.84081152 -0.68996920

C -5.71462651 1.64130982 -2.78985730

C -3.28576278 4.72280004 -1.42213776

C -2.17486510 5.54227518 -1.39958083

C 0.29947954 5.27995631 -1.75518287

C -7.13611286 2.88331421 -0.73841953

H -5.22215487 3.24854893 0.16346680

H -5.15392988 1.17111991 -3.59118049

C -7.10245634 1.69846360 -2.84735936

H -4.32133585 5.01252780 -1.32538433

H -2.14921366 6.61673363 -1.27879961

H 0.37115060 6.32248771 -1.46437825

C 1.40621017 4.66770489 -2.24841789

C -7.82307431 2.31201160 -1.81504328

H -7.70420481 3.32982353 0.06949530

H -7.63493763 1.25515406 -3.68039535

H 1.33672654 3.64737728 -2.59784822

C 2.72494013 5.24605135 -2.39901391

C -9.30894148 2.30817210 -1.77295846

C 3.76487006 4.42604460 -2.81983174

C 3.06977463 6.62613626 -2.15957947

O -9.86167094 1.70783217 -2.84753515

O -9.97068012 2.77936089 -0.86651699

H 3.54618455 3.37737296 -3.00178837

C 5.09944766 4.88124671 -2.99870882

N 2.24263990 7.59614682 -1.75428968

C 4.42717935 7.09340191 -2.38854503

C -11.29313366 1.58183606 -2.80074216

H 5.85365829 4.16229617 -3.30406888

C 5.44866797 6.19284146 -2.79640172

S 3.12820329 8.98183619 -1.68287769

N 4.58625996 8.40489693 -2.17097021

H -11.57477108 1.12086302 -3.74745419

H -11.75963417 2.56363584 -2.69381750

H -11.58379800 0.94537814 -1.96231852

H 6.46099027 6.55494965 -2.92809671

C 2.22840699 -3.58114999 1.38255110

C 3.44976214 -2.87138956 1.43347073

C 3.50529998 -1.48926964 1.56676945

C 2.30004869 -0.78932459 1.64423562

C 1.05925562 -1.47572865 1.63120617

C 1.03218441 -2.86356304 1.50704472

H 4.37915273 -3.41374631 1.30079525

H 4.45952304 -0.97656785 1.55684582

H 0.07944773 -3.38449180 1.49199406

C 0.67362641 0.79295620 1.68809282

C -0.06525831 1.97632115 1.71471829

C -1.45073451 1.87975303 1.73164771

C -2.13018598 0.63695353 1.75697344

C -1.36697106 -0.53619783 1.72794765

C 0.02373545 -0.46638187 1.68170152

H 0.42017708 2.94434810 1.68353296

H -2.02922748 2.79713006 1.69753599

H -1.84997152 -1.50643679 1.77566715

N 2.05759947 0.58764483 1.68009060

C 3.06695400 1.57930748 1.61788478

C 3.00453972 2.61546037 0.67744153

C 4.17203166 1.49231843 2.47164767

C 4.04596257 3.53222670 0.58280720

H 2.17024000 2.66790445 -0.00955715

C 5.21983923 2.39792724 2.36167229

H 4.20372157 0.71496210 3.22757020

C 5.17617022 3.42471579 1.40646710

H 4.00209297 4.31771327 -0.16045731

H 6.07885690 2.31792552 3.01879454

N 6.26689622 4.31232674 1.27161429

C 6.06941654 5.69691542 1.05589544

C 4.95055273 6.36156290 1.58698812

C 6.99988481 6.44541187 0.32541310

C 4.77241479 7.72281095 1.38449418

H 4.21775769 5.80386256 2.15937223

C 6.84033476 7.81862658 0.14647408

H 7.86470892 5.94937925 -0.10104733

C 5.71971814 8.46768306 0.67147102

H 3.90534321 8.23515641 1.78859207

H 7.57869742 8.36227096 -0.43001757

C 7.58347536 3.79001874 1.39228907

C 7.95154928 2.63794889 0.67847715

C 8.53062721 4.39886194 2.21869271

C 9.22649561 2.10708082 0.80038076

H 7.22214707 2.15789266 0.03393886

C 9.82409724 3.88562296 2.32784158

H 8.25539347 5.28914693 2.77467785

C 10.17661017 2.72993642 1.62248422

H 9.51547278 1.21421209 0.25566007

H 10.53603146 4.38506914 2.97413664

O 5.45276703 9.80131732 0.53124493

O 11.40677203 2.13742703 1.66489581

C 6.34820324 10.56696832 -0.26068944

H 7.35674737 10.57932197 0.17424088

H 6.39129689 10.18090550 -1.28543705

H 5.94834826 11.58276060 -0.26569635

C 12.39478287 2.72610745 2.49196055

H 13.28640604 2.10622473 2.38370731

H 12.62558716 3.75264296 2.17680133

H 12.08504174 2.73634653 3.54556955

C -3.60609093 0.57622582 1.82708777

C -4.31707120 -0.55057861 1.37915284

C -4.36987870 1.62572963 2.36398800

C -5.70153757 -0.62088623 1.43908959

H -3.77660770 -1.38946737 0.95585875

C -5.75903834 1.58386475 2.39565020

H -3.86997519 2.49835227 2.77284223

C -6.45703107 0.46513581 1.91138021

H -6.20738347 -1.51592539 1.09551440

H -6.31196767 2.42563432 2.79659904

C 2.22520659 -5.03300657 1.10822892

C 3.27978478 -5.86882462 1.51239398

C 1.18892828 -5.62774545 0.36770245

C 3.31787961 -7.21531735 1.17220024

H 4.09010720 -5.45633733 2.10569505

C 1.20379925 -6.97703833 0.03869098

H 0.36223679 -5.01420563 0.02300198

C 2.27971000 -7.79285665 0.42361039

H 4.15183259 -7.83233004 1.48917998

H 0.38992372 -7.40609738 -0.53595758

N -7.86704825 0.44034162 1.90288510

N 2.31881135 -9.15550908 0.05957005

C -8.60757577 -0.21835204 0.89074679

C -8.08497126 -0.44168130 -0.38776339

C -9.92962279 -0.62850629 1.14263669

C -8.85207384 -1.03087310 -1.39170614

H -7.07698942 -0.12665939 -0.62002887

C -10.70511199 -1.19228019 0.13995669

H -10.35626620 -0.47687366 2.12744936

C -10.17747823 -1.39045690 -1.14214603

H -8.41016314 -1.16002708 -2.37187594

H -11.73122751 -1.48872314 0.33277110

C -8.57169967 1.25551876 2.84009832

C -9.45971719 2.24971017 2.40114057

C -8.36980772 1.07834828 4.20899679

C -10.12950536 3.04146410 3.32301061

H -9.63186986 2.39037067 1.33875902

C -9.02399929 1.88600002 5.14093460

H -7.68279356 0.30899829 4.54730070

C -9.91352242 2.87123885 4.69824871

H -10.82043791 3.81228849 2.99739493

H -8.84200942 1.72888597 6.19735079

C 1.80252292 -9.57273875 -1.19438949

C 2.08766002 -8.84597058 -2.36270475

C 1.01285189 -10.72094397 -1.29778747

C 1.58341031 -9.25050952 -3.58907906

H 2.70594214 -7.95658614 -2.29639010

C 0.52236577 -11.14859657 -2.53268570

H 0.78688756 -11.29145548 -0.40298749

C 0.79950082 -10.40914715 -3.68682214

H 1.79883719 -8.69262148 -4.49468124

H -0.08220942 -12.04672714 -2.57588095

C 2.85145755 -10.12345951 0.95044402

C 3.69350578 -11.14209336 0.47478754

C 2.54646899 -10.08871839 2.31392369

C 4.20453740 -12.09799193 1.33954991

H 3.93742704 -11.17893182 -0.58179143

C 3.07767511 -11.03257855 3.19418810

H 1.89365962 -9.30914547 2.69261732

C 3.90660374 -12.04906134 2.70874291

H 4.85437856 -12.88859554 0.97854453

H 2.82475816 -10.97141614 4.24598941

O -11.03129222 -1.91907109 -2.07543221

O -10.61310760 3.70971031 5.51844813

C -10.42857393 3.57286170 6.91644352

H -9.38442966 3.75337760 7.20512556

H -11.06686828 4.32725461 7.37956068

H -10.72989728 2.57685805 7.26756397

C -10.53117922 -2.08785736 -3.38954946

H -9.69419190 -2.79889856 -3.41519980

H -11.35899817 -2.48531784 -3.97970159

H -10.19955191 -1.13305790 -3.81923080

O 4.46997906 -13.02896530 3.47681846

O 0.36196324 -10.72813094 -4.94142671

C -0.43349665 -11.89138193 -5.08595169

H -1.36588173 -11.81665804 -4.51032474

H -0.67025038 -11.96428006 -6.14878497

H 0.10953503 -12.79292829 -4.77193619

C 4.19392341 -13.01752099 4.86619660

H 3.12020410 -13.13485450 5.06532408

H 4.73408184 -13.86698335 5.28770060

H 4.54643638 -12.09145939 5.33993374
